# Supplementary material for: Phylogenetic Analysis of Spliceosome SF3a2 in Different Plant Species
Source: Int J Mol Sci. 2023 Mar 9;24(6):5232. doi: 10.3390/ijms24065232 (PMC10049718; doi:10.3390/ijms24065232)
Supplement: Supplementary file 1 [file ijms-24-05232-s001.zip › Supplementary Figures.pdf]

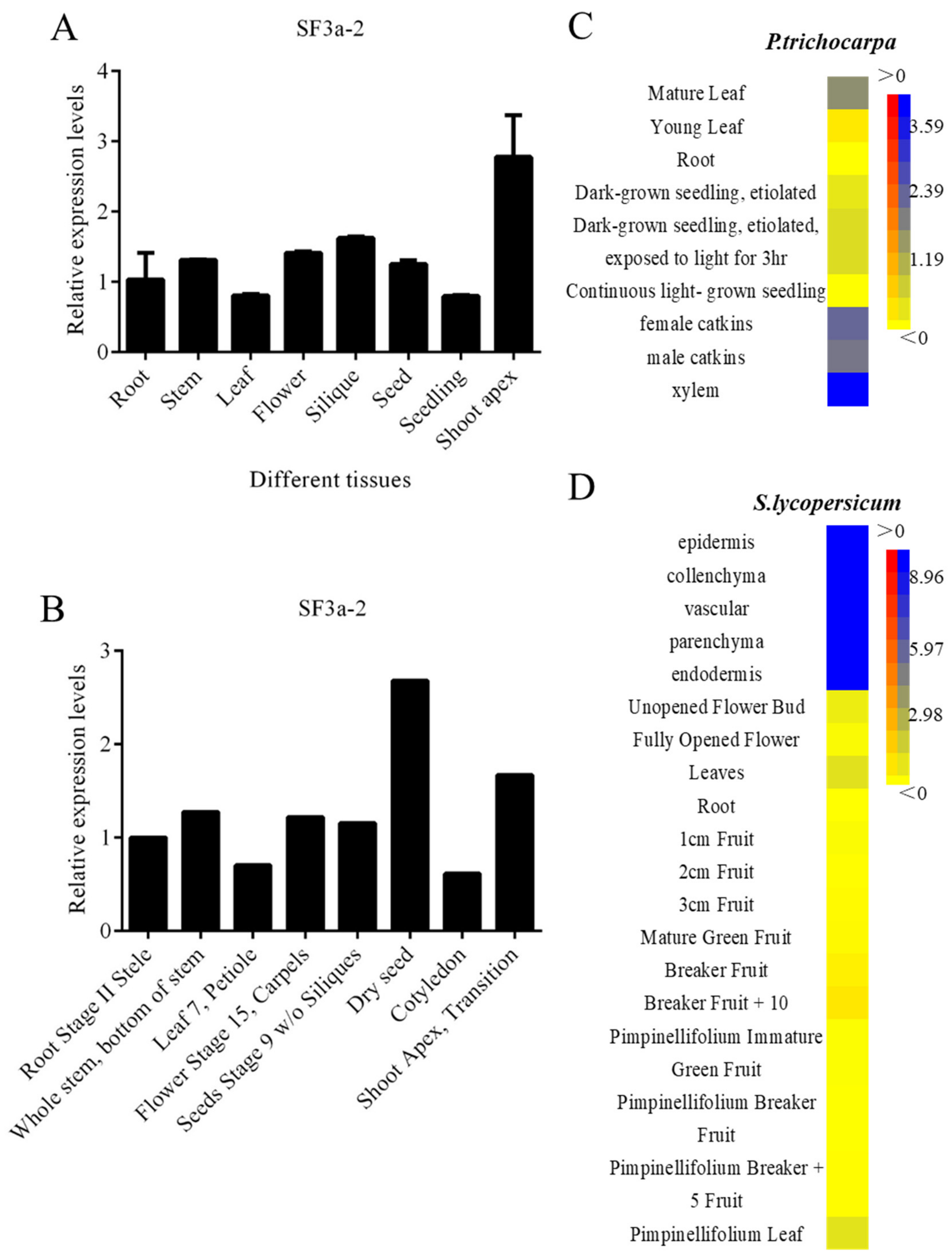

Supplementary Figure S1 Spatial and temporal expressions of several dicot *SF3a2s*. (A) Expression of *ATSF3a2* with different tissues. *GAPDH* as internal reference. (B)Spatiotemporal expression patterns of *ATSF3a2* with different tissues. Data from BAR. (C)Spatiotemporal expression patterns of *PtSF3a2* with different tissues. (D)Spatiotemporal expression patterns of *SlSF3a2* with different tissues. Expression data was extracted using the plant eGFP browser and presented in heatmap format. Red and blue represented high and low expression in different tissues and stages, respectively. The bigger the difference, the darker the color.

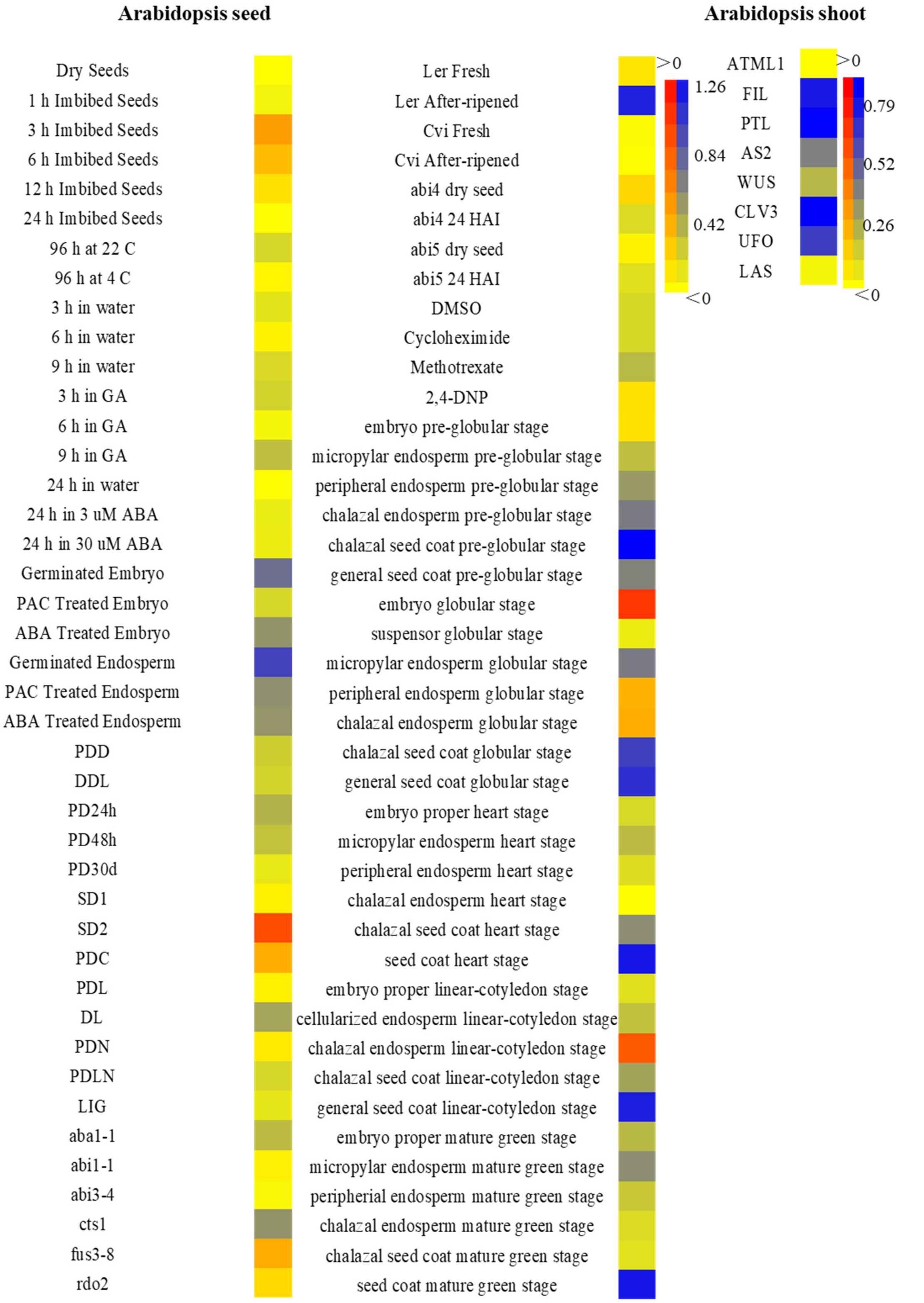

Supplementary Figure S2 Spatial and temporal expressions of Arabidopsis *SF3a2* in different tissues. Expression data was extracted using the plant eGFP browser and presented in heatmap format. Red and blue represented high and low expression in seed and shoot, respectively. The bigger the difference, the darker the color.

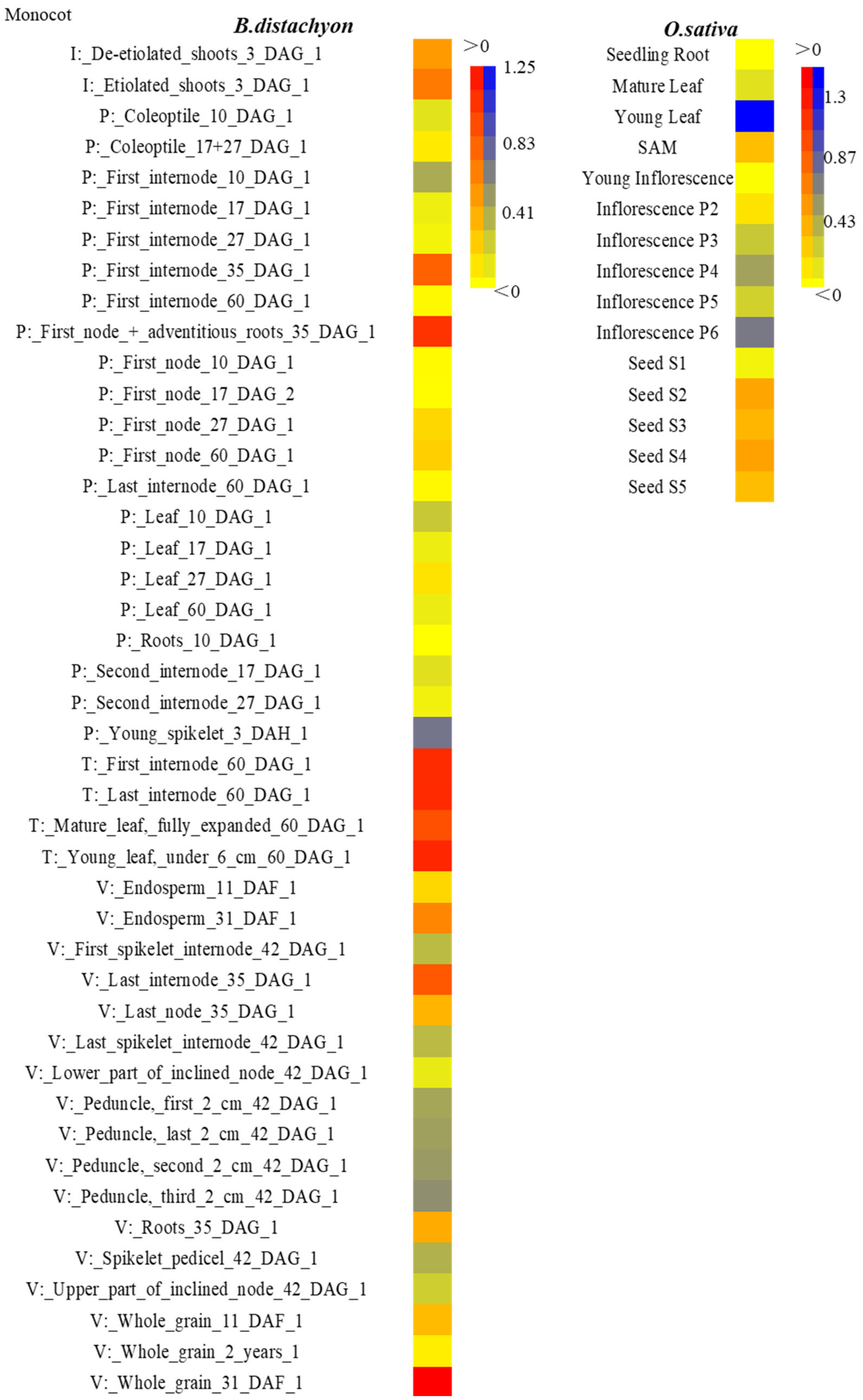

Supplementary Figure S3 Spatial and temporal expressions of several monocot *SF3a2s*. Expression data was extracted using the plant eGFP browser and presented in heatmap format. Red and blue represented high and low expression in different tissues and stages, respectively. The bigger the difference, the darker the color.

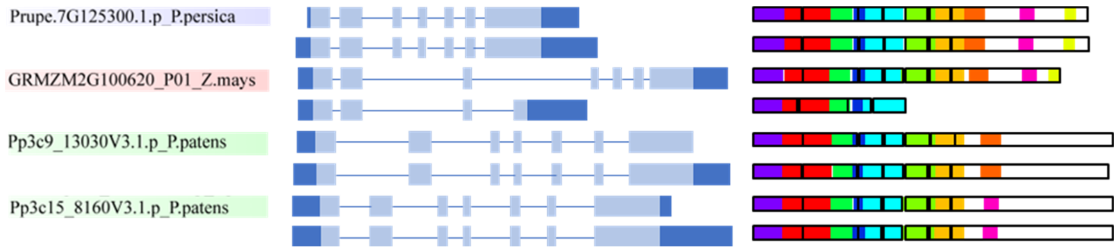

Supplementary Figure S4 Gene structure of *SF3a2s* with multiple transcripts. Alternative splicing events in plant *SF3a2s* genes. The protein encoded by each primary transcript is listed to the right of the corresponding transcript with conserved protein motifs illustrated (the same as in Fig. 2).

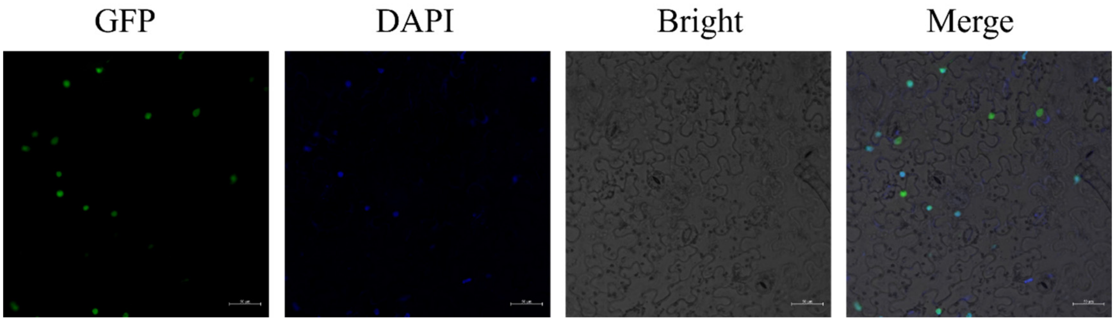

Supplementary Figure S5 Nuclear localization of ATSF3a2 in tobacco cells.
Nuclei were labeled with 4',6 -diamidino-2-phenylindole (DAPI).
