# Supplementary material for: Phylogenetic Analysis of Spliceosome SF3a2 in Different Plant Species
Source: Int J Mol Sci. 2023 Mar 9;24(6):5232. doi: 10.3390/ijms24065232 (PMC10049718; doi:10.3390/ijms24065232)
Supplement: Supplementary file 1 [file ijms-24-05232-s001.zip › Supplementary Table S1.pdf]

Supplementary Table S1 Sequence summary on phylogenetic analysis.

Plant species	Common name	No. of	Sequence initial *e.g.*
Amaranthus hypochondriacus	Prince-of-Wales feather	2	*AHYPO_001149-RA* **(AhySYF3A2a)**<br>*AHYPO_000598-RA* **(AhySYF3A2b)**
Ananas comosus	Pineapple	1	*Aco024234.1* **(AncoSYF3A2)**
Aquilegia coerulea	Colorado blue columbine	1	*Aqcoe2G212900.1.p* **(AqcoSYF3A2)**
Arabidopsis halleri		2	*Araha.1971s0014.1.p* **(AhaSYF3A2a)**<br>*Araha.1905s0002.1.p* **(AhaSYF3A2b)**
Arabidopsis lyrata	Arabidopsis	1	*AL4G28000.t1* **(AlySYF3A2)**
Arabidopsis thaliana	Arabidopsis	1	*AT2G32600.1* **(AthSYF3A2)**
Brachypodium distachyon	Purple false brome	1	*Bradi1g67320.1.p* **(BdiSYF3A2)**
Brachypodium stacei		1	*Brast02G124800.1.p* **(BstSYF3A2)**
Brassica oleracea capitata	Cabbage	1	*Bol013469* **(BolSYF3A2)**
Boechera stricta		1	*Bostr.23794s0853***(BstSYF3A2)**
Brassica rapa		1	*Brara.E01157.1.p* **(BraSYF3A2)**
Capsella grandiflora		2	*Cagra.0804s0006.1.p* **(CgrSYF3A2a)**<br>*Cagra.4714s0005.1.p* **(CgrSYF3A2b)**
Capsella rubella	Pink shepherd's-purse	2	*Carubv10007569m* **(CruSYF3A2a)**<br>*Carubv10023821m* **(CruSYF3A2b)**
Chenopodium quinoa	Quinoa	1	*AUR62026953-RA* **(CquSYF3A2)**
Chlamydomonas reinhardtii		1	*Cre06.g252700.t1.2* **(CreSYF3A2)**
Coccomyxa subellipsoidea		1	*53193* **(CsuSYF3A2)**
Cucumis sativus	Cucumber	1	*Cucsa.306500.1* **(CsaSYF3A2)**
Daucus carota		1	*DCAR_020804* **(DcaSYF3A2)**
Dunaliella salina		1	*Dusal.0694s00005.1.p* **(DsaSYF3A2)**
Eucalyptus grandis	Flooded gum	1	*Eucgr.K02436.1.p* **(EgrSYF3A2)**
Eutrema		1	*Thhalv10017051m* **(EsaSYF3A2)**

salsugineum			
Fragaria vesca	Wild strawberry	1	*mrna09805.1-v1.0-hybrid* **(FveSYF3A2)**
Glycine max	Soybean	2	*Glyma.15G005900.1.p* **(GmaSYF3A2a)**<br>*Glyma.13G367400.1.p* **(GmaSYF3A2b)**
Gossypium hirsutum	Upland cotton	2	*Gohir.D09G140400.1.p* **(GhiSYF3A2a)**<br>*Gohir.A09G144600.1.p* **(GhiSYF3A2b)**
Gossypium raimondii	Cotton	1	*Gorai.006G161000.1* **(GraSYF3A2)**
Kalanchoe fedtschenkoi		2	*Kaladp0098s0047.1.p* **(KfeSYF3A2a)**<br>*Kaladp0011s0266.1.p* **(KfeSYF3A2b)**
Kalanchoe laxiflora		4	*Kalax.1083s0001.1.p* **(KlaSYF3A2a)**<br>*Kalax.0420s0030.1.p* **(KlaSYF3A2b)**<br>*Kalax.0178s0076.1.p* **(KlaSYF3A2c)**<br>*Kalax.0186s0022.1.p* **(KlaSYF3A2d)**
Linum usitatissimum	Flax	1	*Lus10022607* **(LusSYF3A2)**
Malus domestica	Apple	2	*MDP0000286077* **(MdoSYF3A2a)**<br>*MDP0000534942* **(MdoSYF3A2b)**
Manihot esculenta	Cassava	1	*Manes.09G061900.1.p* **(MesSYF3A2)**
Marchantia polymorpha	Common liverwort	1	*Mapoly0069s0004.1.p* **(MpoSYF3A2)**
Medicago truncatula	Barrel medic	1	*Medtr8g009970.1* **(MtrSYF3A2)**
Micromonas pusillaCCMP1545		1	*44451* **(MpuSYF3A2)**
Micromonas spRCC299		1	*63373* **(MspSYF3A2)**
Mimulus guttatus	Seep monkeyflower	1	*Migut.J00456.1.p* **(MguSYF3A2)**
Oropetium thomaeum		1	*Oropetium_20150105_00778A* **(OthSYF3A2)**
Oryza sativa	Rice	1	*LOC_Os03g15700.1* **(OsaSYF3A2)**
Ostreococcus lucimarinus	Green algae	1	*13495* **(OluSYF3A2)**
Panicum hallii		1	*Pahal.H02490.1* **(PhaSYF3A2)**

Panicum virgatum	Switchgrass	3	*Pavir.9NG665400.1.p* **(PviSYF3A2a)**<br>*Pavir.9KG512300.1.p* **(PviSYF3A2b)**<br>*Pavir.9KG504400.1.p* **(PviSYF3A2c)**
Phaseolus vulgaris	Common bean	1	*Phvul.005G180000.1.p* **(PvuSYF3A2)**
Physcomitrella patens	Moss	2	*Pp3c9_13030V3.1.p* **(PpaSYF3A2a)**<br>*Pp3c15_8160V3.1.p* **(PpaSYF3A2b)**
Populus deltoides	Necklace poplar	1	*Podel.06G130900.1.p* **(PdeSYF3A2)**
Populus trichocarpa	Cottonwood	2	*Potri.006G121300.1* **(PtrSYF3A2a)**<br>*Potri.016G097600.1* **(PtrSYF3A2b)**
Prunus persica	Peach	2	*Prupe.4G071900.1.p* **(PpeSYF3A2a)**<br>*Prupe.7G125300.1.p* **(PpeSYF3A2b)**
Ricinus communis		1	*30078.m002249* **(RcoSYF3A2)**
Salix purpurea	Purple willow	1	*SapurV1A.1059s0020.1.p* **(SpuSYF3A2)**
Selaginella moellendorffii	Spikemoss	1	*180432* **(SmoSYF3A2)**
Setaria italica	Foxtail millet	1	*Seita.9G455700.1.p* **(SitSYF3A2)**
Setaria viridis	Green foxtail	1	*Sevir.9G459400.1.p* **(SviSYF3A2)**
Solanum lycopersicum	Tomato	1	*Solyc09g008470.2.1* **(SlySYF3A2)**
Solanum tuberosum	Potato	1	*PGSC0003DMP400006880* **(StuSYF3A2)**
Sorghum bicolor	Sorghum	1	*Sobic.001G428400.1.p* **(SbiSYF3A2)**
Sphagnum fallax		2	*Sphfalx0014s0238.1.p* **(SfaSYF3A2a)**<br>*Sphfalx0006s0301.1.p* **(SfaSYF3A2b)**
Theobroma cacao	Cacao tree	1	*Thecc1EG022302t1* **(TcaSYF3A2)**
Trifolium pratense	Red clover	1	*Tp57577_TGAC_v2_mRNA17966* **(TprSYF3A2)**
Triticum aestivum	Common wheat	3	*Traes_4DL_87F981684.1* **(TaeSYF3A2a)**<br>*Traes_4AS_74660DF99.1* **(TaeSYF3A2b)**<br>*Traes_4BL_3EA2CD6B0.1* **(TaeSYF3A2c)**
Vitis vinifera	Grape	1	*GSVIVT01033687001* **(VviSYF3A2)**
Volvox carteri		1	*Vocar.0038s0046.1.p* **(VcaSYF3A2)**
Zea mays	Maize	4	*Zm00008a035638_P01* **(ZemaSYF3A2a)**<br>*Zm00008a001017_P01***(ZemaSYF3A2b)**<br>*GRMZM2G100620_P01***(ZemaSYF3A2c)**

			*GRMZM2G307906_P01***(ZemaSYF3A2d)**
Zostera marina	Common eelgrass	1	*Zosma64g00720.1* **(ZomaSYF3A2)**

The species of plant lineage were listed and the number of 61 chosen was mentioned.
The species with bold character were selected for further analysis in Figure 1.
