# Supplementary material for: Phylogenetic Analysis of Spliceosome SF3a2 in Different Plant Species
Source: Int J Mol Sci. 2023 Mar 9;24(6):5232. doi: 10.3390/ijms24065232 (PMC10049718; doi:10.3390/ijms24065232)
Supplement: Supplementary file 1 [file ijms-24-05232-s001.zip › Supplementary Table S2.pdf]

Supplementary Table S2 List of cis-elements identified among plant SF3a2s.

AL4G28000 (*Arabidopsis lyrata*)

+ ARE

Site Name	Organism	Position	Strand	Matrix score.	sequence	function
ARE	Zea mays	1460	+	6	TGGTTT	cis-acting regulatory element essential for the anaerobic induction

+ CCGTCC-box

Site Name	Organism	Position	Strand	Matrix score.	sequence	function
CCGTCC-box	Arabidopsis thaliana	1450	+	6	CCGTCC	cis-acting regulatory element related to meristem specific activation

+ HSE

Site Name	Organism	Position	Strand	Matrix score.	sequence	function
HSE	Brassica oleracea	238	+	9	AAAAAATTTC	cis-acting element involved in heat stress responsiveness

+ MBS

Site Name	Organism	Position	Strand	Matrix score.	sequence	function
MBS	Arabidopsis thaliana	1270	+	6	TAACTG	MYB binding site involved in drought-inducibility

+ Skn-1_motif

Site Name	Organism	Position	Strand	Matrix score.	sequence	function
Skn-1_motif	Oryza sativa	142	+	5	GTCAT	cis-acting regulatory element required for endosperm expression
Skn-1_motif	Oryza sativa	1439	+	5	GTCAT	cis-acting regulatory element required for endosperm expression
Skn-1_motif	Oryza sativa	489	+	5	GTCAT	cis-acting regulatory element required for endosperm expression

+ TGA-element

Site Name	Organism	Position	Strand	Matrix score.	sequence	function
TGA-element	Brassica oleracea	181	-	6	AACGAC	auxin-responsive element

+ as-2-box

Site Name	Organism	Position	Strand	Matrix score.	sequence	function
as-2-box	Nicotiana	1099	+	9	GATAatGATG	involved in shoot-specific expression and light

	tabacum					responsiveness

Araha.1971s0014 (*Arabidopsis halleri*)

+ MBS

Site Name	Organism	Position	Strand	Matrix score.	sequence	function
MBS	Arabidopsis thaliana	942	+	6	TAACTG	MYB binding site involved in drought-inducibility

+ Skn-1_motif

Site Name	Organism	Position	Strand	Matrix score.	sequence	function
Skn-1_motif	Oryza sativa	152	+	5	GTCAT	cis-acting regulatory element required for endosperm expression

+ TC-rich repeats

Site Name	Organism	Position	Strand	Matrix score.	sequence	function
TC-rich repeats	Nicotiana tabacum	1050	-	9	ATTTTCTTCA	cis-acting element involved in defense and stress responsiveness
TC-rich repeats	Nicotiana tabacum	1063	-	9	ATTTTCTCCA	cis-acting element involved in defense and stress responsiveness

+ as-2-box

Site Name	Organism	Position	Strand	Matrix score.	sequence	function
as-2-box	Nicotiana tabacum	771	+	9	GATAatGATG	involved in shoot-specific expression and light responsiveness

AT2G32600 (*Arabidopsis thaliana*)

+ ARE

Site Name	Organism	Position	Strand	Matrix score.	sequence	function
ARE	Zea mays	1470	-	6	TGGTTT	cis-acting regulatory element essential for the anaerobic induction

+ CGTCA-motif

Site Name	Organism	Position	Strand	Matrix score.	sequence	function
CGTCA-motif	Hordeum vulgare	1375	-	5	CGTCA	cis-acting regulatory element involved in the MeJA-responsiveness

+ GARE-motif

Site Name	Organism	Position	Strand	Matrix score.	sequence	function
GARE-motif	Brassica oleracea	183	+	7	TCTGTTG	gibberellin-responsive element

+ Skn-1_motif

Site Name	Organism	Position	Strand	Matrix score.	sequence	function
Skn-1_motif	Oryza sativa	111	+	5	GTCAT	cis-acting regulatory element required for endosperm expression
Skn-1_motif	Oryza sativa	1374	-	5	GTCAT	cis-acting regulatory element required for endosperm expression
Skn-1_motif	Oryza sativa	458	+	5	GTCAT	cis-acting regulatory element required for endosperm expression

+ TGACG-motif

Site Name	Organism	Position	Strand	Matrix score.	sequence	function
TGACG-motif	Hordeum vulgare	1375	+	5	TGACG	cis-acting regulatory element involved in the MeJA-responsiveness

+ as-2-box

Site Name	Organism	Position	Strand	Matrix score.	sequence	function
as-2-box	Nicotiana tabacum	1041	+	9	GATAatGATG	involved in shoot-specific expression and light responsiveness

Carubv10023821m.g (*Capsella rubella*)

\+ ARE

Site Name	Organism	Position	Strand	Matrix score.	sequence	function
ARE	Zea mays	1206	-	6	TGGTTT	cis-acting regulatory element essential for the anaerobic induction

\+ HSE

Site Name	Organism	Position	Strand	Matrix score.	sequence	function
HSE	Brassica oleracea	931	+	9	AAAAAATTTC	cis-acting element involved in heat stress responsiveness

\+ MBS

Site Name	Organism	Position	Strand	Matrix score.	sequence	function
MBS	Arabidopsis thaliana	688	+	6	TAACTG	MYB binding site involved in drought-inducibility

\+ Skn-1_motif

Site Name	Organism	Position	Strand	Matrix score.	sequence	function
Skn-1_motif	Oryza sativa	1225	+	5	GTCAT	cis-acting regulatory element required for endosperm expression

\+ TC-rich repeats

Site Name	Organism	Position	Strand	Matrix score.	sequence	function
TC-rich repeats	Nicotiana tabacum	974	-	9	ATTCTCTAAC	cis-acting element involved in defense and stress responsiveness
TC-rich repeats	Nicotiana tabacum	1130	+	9	ATTTTCTTCA	cis-acting element involved in defense and stress responsiveness

\+ TCA-element

Site Name	Organism	Position	Strand	Matrix score.	sequence	function
TCA-element	Brassica oleracea	319	+	9	GAGAAGAATA	cis-acting element involved in salicylic acid responsiveness

\+ TGA-element

Site Name	Organism	Position	Strand	Matrix score.	sequence	function
TGA-element	Brassica oleracea	286	-	6	AACGAC	auxin-responsive element

Cagra.0804s0006 (*Capsella grandiflora*)

+ ARE

Site Name	Organism	Position	Strand	Matrix score.	sequence	function
ARE	Zea mays	203	-	6	TGGTTT	cis-acting regulatory element essential for the anaerobic induction
ARE	Zea mays	1218	-	6	TGGTTT	cis-acting regulatory element essential for the anaerobic induction
ARE	Zea mays	529	+	6	TGGTTT	cis-acting regulatory element essential for the anaerobic induction

+ AuxRR-core

Site Name	Organism	Position	Strand	Matrix score.	sequence	function
AuxRR-core	Nicotiana tabacum	726	-	7	GGTCCAT	cis-acting regulatory element involved in auxin responsiveness

+ CCGTCC-box

Site Name	Organism	Position	Strand	Matrix score.	sequence	function
CCGTCC-box	Arabidopsis thaliana	245	-	6	CCGTCC	cis-acting regulatory element related to meristem specific activation

+ HSE

Site Name	Organism	Position	Strand	Matrix score.	sequence	function
HSE	Brassica oleracea	281	+	9	AGAAAATTCG	cis-acting element involved in heat stress responsiveness
HSE	Lycopersicon esculentum	295	+	8	CNNGAANNTTCNNG	cis-acting element involved in heat stress responsiveness

+ MBS

Site Name	Organism	Position	Strand	Matrix score.	sequence	function
MBS	Arabidopsis thaliana	965	+	6	TAACTG	MYB binding site involved in drought-inducibility

+ Skn-1_motif

Site Name	Organism	Position	Strand	Matrix score.	sequence	function
Skn-1_motif	Oryza sativa	17	+	5	GTCAT	cis-acting regulatory element required for endosperm expression
Skn-1_motif	Oryza sativa	1237	+	5	GTCAT	cis-acting regulatory element required for endosperm expression
Skn-1_motif	Oryza sativa	556	+	5	GTCAT	cis-acting regulatory element required for

endosperm expression

+ TCA-element

Site Name	Organism	Position	Strand	Matrix score.	sequence	function
TCA-element	Brassica oleracea	608	+	9	GAGAAGAATA	cis-acting element involved in salicylic acid responsiveness
TCA-element	Brassica oleracea	1242	+	9	GAGAAGAATA	cis-acting element involved in salicylic acid responsiveness
TCA-element	Brassica oleracea	1049	+	9	GAGAAGAATA	cis-acting element involved in salicylic acid responsiveness

+ TGA-element

Site Name	Organism	Position	Strand	Matrix score.	sequence	function
TGA-element	Brassica oleracea	47	-	6	AACGAC	auxin-responsive element
TGA-element	Brassica oleracea	575	-	6	AACGAC	auxin-responsive element

+ WUN-motif

Site Name	Organism	Position	Strand	Matrix score.	sequence	function
WUN-motif	Brassica oleracea	680	-	9	TCATTACGAA	wound-responsive element

Bostr.23794s0853 (*Boechera stricta*)

+ ABRE

Site Name	Organism	Position	Strand	Matrix score.	sequence	function
ABRE	Arabidopsis thaliana	1145	-	6	CACGTG	cis-acting element involved in the abscisic acid responsiveness

+ ARE

Site Name	Organism	Position	Strand	Matrix score.	sequence	function
ARE	Zea mays	177	-	6	TGGTTT	cis-acting regulatory element essential for the anaerobic induction

+ Box-W1

Site Name	Organism	Position	Strand	Matrix score.	sequence	function
Box-W1	Petroselinum crispum	943	+	6	TTGACC	fungal elicitor responsive element

+ GARE-motif

Site Name	Organism	Position	Strand	Matrix score.	sequence	function
GARE-motif	Brassica oleracea	993	-	7	AAACAGA	gibberellin-responsive element

+ GCN4_motif

Site Name	Organism	Position	Strand	Matrix score.	sequence	function
GCN4_motif	Oryza sativa	495	-	7	TGTGTCA	cis-regulatory element involved in endosperm expression

+ HSE

Site Name	Organism	Position	Strand	Matrix score.	sequence	function
HSE	Brassica oleracea	46	+	9	AAAAAATTTC	cis-acting element involved in heat stress responsiveness
HSE	Brassica oleracea	1131	-	9	AAAAAATTTC	cis-acting element involved in heat stress responsiveness

+ Skn-1_motif

Site Name	Organism	Position	Strand	Matrix score.	sequence	function
Skn-1_motif	Oryza sativa	494	-	5	GTCAT	cis-acting regulatory element required for endosperm expression
Skn-1_motif	Oryza sativa	950	-	5	GTCAT	cis-acting regulatory element required for endosperm expression

+ TC-rich repeats

Site Name	Organism	Position	Strand	Matrix score.	sequence	function
TC-rich repeats	Nicotiana tabacum	345	+	9	ATTTTCTTCA	cis-acting element involved in defense and stress responsiveness
TC-rich repeats	Nicotiana tabacum	817	+	9	ATTTTCTCCA	cis-acting element involved in defense and stress responsiveness

+ TCA-element

Site Name	Organism	Position	Strand	Matrix score.	sequence	function
TCA-element	Brassica oleracea	1031	+	9	GAGAAGAATA	cis-acting element involved in salicylic acid responsiveness

Bol013469 (*Brassica oleracea capitata*)

+ ARE

Site Name	Organism	Position	Strand	Matrix score.	sequence	function
ARE	Zea mays	395	+	6	TGGTTT	cis-acting regulatory element essential for the anaerobic induction
ARE	Zea mays	1127	-	6	TGGTTT	cis-acting regulatory element essential for the anaerobic induction

+ AT-rich sequence

Site Name	Organism	Position	Strand	Matrix score.	sequence	function
AT-rich sequence	Pisum sativum	18	+	9	TAAAATACT	element for maximal elicitor-mediated activation (2copies)

+ CCGTCC-box

Site Name	Organism	Position	Strand	Matrix score.	sequence	function
CCGTCC-box	Arabidopsis thaliana	1347	-	6	CCGTCC	cis-acting regulatory element related to meristem specific activation

+ ERE

Site Name	Organism	Position	Strand	Matrix score.	sequence	function
ERE	Dianthus caryophyllus	240	+	8	ATTTCAAA	ethylene-responsive element

+ GARE-motif

Site Name	Organism	Position	Strand	Matrix score.	sequence	function
GARE-motif	Brassica oleracea	704	+	7	AAACAGA	gibberellin-responsive element
GARE-motif	Brassica oleracea	788	-	7	AAACAGA	gibberellin-responsive element

+ HSE

Site Name	Organism	Position	Strand	Matrix score.	sequence	function
HSE	Brassica oleracea	30	-	9	AAAAAATTTC	cis-acting element involved in heat stress responsiveness
HSE	Brassica oleracea	235	+	9	AAAAAATTTC	cis-acting element involved in heat stress responsiveness

+ LTR

Site Name	Organism	Position	Strand	Matrix score.	sequence	function

LTR	Hordeum vulgare	401	-	6	CCGAAA	cis-acting element involved in low-temperature responsiveness
LTR	Hordeum vulgare	484	-	6	CCGAAA	cis-acting element involved in low-temperature responsiveness
LTR	Hordeum vulgare	409	-	6	CCGAAA	cis-acting element involved in low-temperature responsiveness
LTR	Hordeum vulgare	1211	-	6	CCGAAA	cis-acting element involved in low-temperature responsiveness

+ Skn-1_motif

Site Name	Organism	Position	Strand	Matrix score.	sequence	function
Skn-1_motif	Oryza sativa	554	+	5	GTCAT	cis-acting regulatory element required for endosperm expression

+ TCA-element

Site Name	Organism	Position	Strand	Matrix score.	sequence	function
TCA-element	Nicotiana tabacum	1288	+	9	CCATCTTTTT	cis-acting element involved in salicylic acid responsiveness
TCA-element	Brassica oleracea	1440	-	9	GAGAAGAATA	cis-acting element involved in salicylic acid responsiveness

Brara.E01157 (*Brassica rapa*)

+ ABRE

Site Name	Organism	Position	Strand	Matrix score.	sequence	function
ABRE	Hordeum vulgare	318	-	9	CGTACGTGCA	cis-acting element involved in the abscisic acid responsiveness

+ ARE

Site Name	Organism	Position	Strand	Matrix score.	sequence	function
ARE	Zea mays	243	+	6	TGGTTT	cis-acting regulatory element essential for the anaerobic induction

+ AT-rich sequence

Site Name	Organism	Position	Strand	Matrix score.	sequence	function
AT-rich sequence	Pisum sativum	1117	-	9	TAAAATACT	element for maximal elicitor-mediated activation (2copies)

+ Box-W1

Site Name	Organism	Position	Strand	Matrix score.	sequence	function
Box-W1	Petroselinum crispum	132	+	6	TTGACC	fungal elicitor responsive element
Box-W1	Petroselinum crispum	655	+	6	TTGACC	fungal elicitor responsive element

+ CGTCA-motif

Site Name	Organism	Position	Strand	Matrix score.	sequence	function
CGTCA-motif	Hordeum vulgare	858	-	5	CGTCA	cis-acting regulatory element involved in the MeJA-responsiveness

+ ERE

Site Name	Organism	Position	Strand	Matrix score.	sequence	function
ERE	Dianthus caryophyllus	1228	-	8	ATTTCAAA	ethylene-responsive element

+ GARE-motif

Site Name	Organism	Position	Strand	Matrix score.	sequence	function
GARE-motif	Brassica oleracea	353	-	7	AAACAGA	gibberellin-responsive element

+ LTR

Site Name	Organism	Position	Strand	Matrix	sequence	function

				Matrix score.		
LTR	Hordeum vulgare	951	+	6	CCGAAA	cis-acting element involved in low-temperature responsiveness
LTR	Hordeum vulgare	1444	+	6	CCGAAA	cis-acting element involved in low-temperature responsiveness

+ P-box

Site Name	Organism	Position	Strand	Matrix score.	sequence	function
P-box	Oryza sativa	403	+	7	CCTTTTG	gibberellin-responsive element

+ Skn-1_motif

Site Name	Organism	Position	Strand	Matrix score.	sequence	function
Skn-1_motif	Oryza sativa	533	+	5	GTCAT	cis-acting regulatory element required for endosperm expression
Skn-1_motif	Oryza sativa	1161	-	5	GTCAT	cis-acting regulatory element required for endosperm expression
Skn-1_motif	Oryza sativa	551	-	5	GTCAT	cis-acting regulatory element required for endosperm expression

+ TATC-box

Site Name	Organism	Position	Strand	Matrix score.	sequence	function
TATC-box	Oryza sativa	1220	-	7	TATCCCA	cis-acting element involved in gibberellin-responsiveness

+ TC-rich repeats

Site Name	Organism	Position	Strand	Matrix score.	sequence	function
TC-rich repeats	Nicotiana tabacum	393	-	9	GTTTTCTTAC	cis-acting element involved in defense and stress responsiveness
TC-rich repeats	Nicotiana tabacum	1386	+	9	ATTTTCTCCA	cis-acting element involved in defense and stress responsiveness

+ TCA-element

Site Name	Organism	Position	Strand	Matrix score.	sequence	function
TCA-element	Brassica oleracea	304	+	9	GAGAAGAATA	cis-acting element involved in salicylic acid responsiveness

+ TGACG-motif

Site Name	Organism	Position	Strand	Matrix score.	sequence	function
TGACG-motif	Hordeum vulgare	858	+	5	TGACG	cis-acting regulatory element involved in the MeJA-responsiveness

Thhalv10017051m.g (*Eutrema salsugineum*)

+ ABRE

Site Name	Organism	Position	Strand	Matrix score.	sequence	function
ABRE	Arabidopsis thaliana	65	+	6	CACGTG	cis-acting element involved in the abscisic acid responsiveness

+ ARE

Site Name	Organism	Position	Strand	Matrix score.	sequence	function
ARE	Zea mays	303	-	6	TGGTTT	cis-acting regulatory element essential for the anaerobic induction
ARE	Zea mays	1204	+	6	TGGTTT	cis-acting regulatory element essential for the anaerobic induction

+ Box-W1

Site Name	Organism	Position	Strand	Matrix score.	sequence	function
Box-W1	Petroselinum crispum	11	+	6	TTGACC	fungal elicitor responsive element

+ CGTCA-motif

Site Name	Organism	Position	Strand	Matrix score.	sequence	function
CGTCA-motif	Hordeum vulgare	1251	-	5	CGTCA	cis-acting regulatory element involved in the MeJA-responsiveness

+ ERE

Site Name	Organism	Position	Strand	Matrix score.	sequence	function
ERE	Dianthus caryophyllus	587	+	8	ATTTCAAA	ethylene-responsive element

+ GARE-motif

Site Name	Organism	Position	Strand	Matrix score.	sequence	function
GARE-motif	Brassica oleracea	342	+	7	AAACAGA	gibberellin-responsive element

+ HSE

Site Name	Organism	Position	Strand	Matrix score.	sequence	function
HSE	Brassica oleracea	582	+	9	AAAAAATTTC	cis-acting element involved in heat stress responsiveness

+ MBSII

Site Name	Organism	Position	Strand	Matrix	sequence	function

				score.		
MBSII	Petunia hybrida	1258	+	11	AAAAGTTAGTTA	MYB binding site involved in flavonoid biosynthetic genes regulation

+ TATC-box

Site Name	Organism	Position	Strand	Matrix score.	sequence	function
TATC-box	Oryza sativa	504	+	7	TATCCCA	cis-acting element involved in gibberellin-responsiveness

+ TC-rich repeats

Site Name	Organism	Position	Strand	Matrix score.	sequence	function
TC-rich repeats	Nicotiana tabacum	1029	-	10	GTTTTCTTAC	cis-acting element involved in defense and stress responsiveness

+ TCA-element

Site Name	Organism	Position	Strand	Matrix score.	sequence	function
TCA-element	Nicotiana tabacum	342	-	9	CCATCTTTTT	cis-acting element involved in salicylic acid responsiveness
TCA-element	Nicotiana tabacum	1164	-	9	CCATCTTTTT	cis-acting element involved in salicylic acid responsiveness

+ TGACG-motif

Site Name	Organism	Position	Strand	Matrix score.	sequence	function
TGACG-motif	Hordeum vulgare	1251	+	5	TGACG	cis-acting regulatory element involved in the MeJA-responsiveness

+ WUN-motif

Site Name	Organism	Position	Strand	Matrix score.	sequence	function
WUN-motif	Brassica oleracea	753	+	9	TCATTACGAA	wound-responsive element

Carubv10007569m.g (*Capsella rubella*)

+ ABRE

Site Name	Organism	Position	Strand	Matrix score.	sequence	function
ABRE	Arabidopsis thaliana	72	-	6	TACGTG	cis-acting element involved in the abscisic acid responsiveness

+ ARE

Site Name	Organism	Position	Strand	Matrix score.	sequence	function
ARE	Zea mays	917	-	6	TGGTTT	cis-acting regulatory element essential for the anaerobic induction

+ CAT-box

Site Name	Organism	Position	Strand	Matrix score.	sequence	function
CAT-box	Arabidopsis thaliana	627	-	6	GCCACT	cis-acting regulatory element related to meristem expression

+ CGTCA-motif

Site Name	Organism	Position	Strand	Matrix score.	sequence	function
CGTCA-motif	Hordeum vulgare	230	-	5	CGTCA	cis-acting regulatory element involved in the MeJA-responsiveness
CGTCA-motif	Hordeum vulgare	632	+	5	CGTCA	cis-acting regulatory element involved in the MeJA-responsiveness
CGTCA-motif	Hordeum vulgare	623	+	5	CGTCA	cis-acting regulatory element involved in the MeJA-responsiveness

+ GARE-motif

Site Name	Organism	Position	Strand	Matrix score.	sequence	function
GARE-motif	Brassica oleracea	124	-	7	AAACAGA	gibberellin-responsive element
GARE-motif	Brassica oleracea	1012	+	7	TCTGTTG	gibberellin-responsive element
GARE-motif	Brassica oleracea	842	-	7	AAACAGA	gibberellin-responsive element

+ HSE

Site Name	Organism	Position	Strand	Matrix score.	sequence	function
HSE	Brassica oleracea	690	-	9	AAAAAATTTC	cis-acting element involved in heat stress responsiveness

+ MBS

Site Name	Organism	Position	Strand	Matrix score.	sequence	function
MBS	Arabidopsis thaliana	584	-	6	TAACTG	MYB binding site involved in drought-inducibility

+ O2-site

Site Name	Organism	Position	Strand	Matrix score.	sequence	function
O2-site	Zea mays	341	+	9	GATGATGTGG	cis-acting regulatory element involved in zein metabolism regulation
O2-site	Zea mays	761	-	9	GATGACATGA	cis-acting regulatory element involved in zein metabolism regulation

+ Skn-1_motif

Site Name	Organism	Position	Strand	Matrix score.	sequence	function
Skn-1_motif	Oryza sativa	633	+	5	GTCAT	cis-acting regulatory element required for endosperm expression
Skn-1_motif	Oryza sativa	1252	-	5	GTCAT	cis-acting regulatory element required for endosperm expression

+ TGACG-motif

Site Name	Organism	Position	Strand	Matrix score.	sequence	function
TGACG-motif	Hordeum vulgare	230	+	5	TGACG	cis-acting regulatory element involved in the MeJA-responsiveness
TGACG-motif	Hordeum vulgare	632	-	5	TGACG	cis-acting regulatory element involved in the MeJA-responsiveness
TGACG-motif	Hordeum vulgare	623	-	5	TGACG	cis-acting regulatory element involved in the MeJA-responsiveness

+ as-2-box

Site Name	Organism	Position	Strand	Matrix score.	sequence	function
as-2-box	Nicotiana tabacum	338	+	9	GATAatGATG	involved in shoot-specific expression and light responsiveness

Cagra.4714s0005 (*Capsella grandiflora*)

+ ABRE

Site Name	Organism	Position	Strand	Matrix score.	sequence	function
ABRE	Arabidopsis thaliana	152	-	6	TACGTG	cis-acting element involved in the abscisic acid responsiveness

+ ARE

Site Name	Organism	Position	Strand	Matrix score.	sequence	function
ARE	Zea mays	50	-	6	TGGTTT	cis-acting regulatory element essential for the anaerobic induction
ARE	Zea mays	997	-	6	TGGTTT	cis-acting regulatory element essential for the anaerobic induction

+ CAT-box

Site Name	Organism	Position	Strand	Matrix score.	sequence	function
CAT-box	Arabidopsis thaliana	707	-	6	GCCACT	cis-acting regulatory element related to meristem expression

+ CGTCA-motif

Site Name	Organism	Position	Strand	Matrix score.	sequence	function
CGTCA-motif	Hordeum vulgare	310	-	5	CGTCA	cis-acting regulatory element involved in the MeJA-responsiveness
CGTCA-motif	Hordeum vulgare	712	+	5	CGTCA	cis-acting regulatory element involved in the MeJA-responsiveness
CGTCA-motif	Hordeum vulgare	703	+	5	CGTCA	cis-acting regulatory element involved in the MeJA-responsiveness

+ GARE-motif

Site Name	Organism	Position	Strand	Matrix score.	sequence	function
GARE-motif	Brassica oleracea	204	-	7	AAACAGA	gibberellin-responsive element
GARE-motif	Brassica oleracea	922	-	7	AAACAGA	gibberellin-responsive element

+ HSE

Site Name	Organism	Position	Strand	Matrix score.	sequence	function
HSE	Brassica oleracea	770	-	9	AAAAAATTTC	cis-acting element involved in heat stress responsiveness

+ MBS

Site Name	Organism	Position	Strand	Matrix score.	sequence	function
MBS	Arabidopsis thaliana	664	-	6	TAACTG	MYB binding site involved in drought-inducibility

+ O2-site

Site Name	Organism	Position	Strand	Matrix score.	sequence	function
O2-site	Zea mays	421	+	9	GATGATGTGG	cis-acting regulatory element involved in zein metabolism regulation
O2-site	Zea mays	841	-	9	GATGACATGA	cis-acting regulatory element involved in zein metabolism regulation

+ Skn-1_motif

Site Name	Organism	Position	Strand	Matrix score.	sequence	function
Skn-1_motif	Oryza sativa	713	+	5	GTCAT	cis-acting regulatory element required for endosperm expression
Skn-1_motif	Oryza sativa	1245	-	5	GTCAT	cis-acting regulatory element required for endosperm expression

+ TC-rich repeats

Site Name	Organism	Position	Strand	Matrix score.	sequence	function
TC-rich repeats	Nicotiana tabacum	1215	-	9	ATTCTCTAAC	cis-acting element involved in defense and stress responsiveness

+ TGACG-motif

Site Name	Organism	Position	Strand	Matrix score.	sequence	function
TGACG-motif	Hordeum vulgare	310	+	5	TGACG	cis-acting regulatory element involved in the MeJA-responsiveness
TGACG-motif	Hordeum vulgare	712	-	5	TGACG	cis-acting regulatory element involved in the MeJA-responsiveness
TGACG-motif	Hordeum vulgare	703	-	5	TGACG	cis-acting regulatory element involved in the MeJA-responsiveness

+ as-2-box

Site Name	Organism	Position	Strand	Matrix score.	sequence	function
as-2-box	Nicotiana tabacum	418	+	9	GATAatGATG	involved in shoot-specific expression and light responsiveness

Araha.1905s0002 (*Arabidopsis halleri*)

+ ABRE

Site Name	Organism	Position	Strand	Matrix score.	sequence	function
ABRE	Arabidopsis thaliana	662	-	6	TACGTG	cis-acting element involved in the abscisic acid responsiveness
ABRE	Arabidopsis thaliana	979	-	6	CACGTG	cis-acting element involved in the abscisic acid responsiveness
ABRE	Arabidopsis thaliana	744	+	6	TACGTG	cis-acting element involved in the abscisic acid responsiveness

+ Box-W1

Site Name	Organism	Position	Strand	Matrix score.	sequence	function
Box-W1	Petroselinum crispum	453	+	6	TTGACC	fungal elicitor responsive element

+ GCN4_motif

Site Name	Organism	Position	Strand	Matrix score.	sequence	function
GCN4_motif	Oryza sativa	247	-	7	TGTGTCA	cis-regulatory element involved in endosperm expression

+ HSE

Site Name	Organism	Position	Strand	Matrix score.	sequence	function
HSE	Brassica oleracea	74	-	9	AAAAAATTTC	cis-acting element involved in heat stress responsiveness
HSE	Brassica oleracea	729	-	9	AAAAAATTTC	cis-acting element involved in heat stress responsiveness
HSE	Brassica oleracea	582	-	9	AAAAAATTTC	cis-acting element involved in heat stress responsiveness
HSE	Brassica oleracea	879	-	9	AAAAAATTTC	cis-acting element involved in heat stress responsiveness
HSE	Brassica oleracea	581	-	9	AGAAAATTCG	cis-acting element involved in heat stress responsiveness
HSE	Brassica oleracea	647	-	9	AAAAAATTTC	cis-acting element involved in heat stress responsiveness

+ MBS

Site Name	Organism	Position	Strand	Matrix score.	sequence	function
MBS	Arabidopsis thaliana	42	+	6	TAACTG	MYB binding site involved in drought-inducibility

+ O2-site

Site Name	Organism	Position	Strand	Matrix score.	sequence	function
O2-site	Zea mays	1120	+	9	GATGATGTGG	cis-acting regulatory element involved in zein metabolism regulation

+ P-box

Site Name	Organism	Position	Strand	Matrix score.	sequence	function
P-box	Oryza sativa	1152	+	10	GCCTTTTGAGT	gibberellin-responsive element
P-box	Oryza sativa	1153	+	7	CCTTTTG	gibberellin-responsive element

+ Skn-1_motif

Site Name	Organism	Position	Strand	Matrix score.	sequence	function
Skn-1_motif	Oryza sativa	152	-	5	GTCAT	cis-acting regulatory element required for endosperm expression
Skn-1_motif	Oryza sativa	246	-	5	GTCAT	cis-acting regulatory element required for endosperm expression

+ TC-rich repeats

Site Name	Organism	Position	Strand	Matrix score.	sequence	function
TC-rich repeats	Nicotiana tabacum	937	+	9	GTTTTCTTAC	cis-acting element involved in defense and stress responsiveness
TC-rich repeats	Nicotiana tabacum	1445	+	9	GTTTTCTTAC	cis-acting element involved in defense and stress responsiveness

+ TCA-element

Site Name	Organism	Position	Strand	Matrix score.	sequence	function
TCA-element	Nicotiana tabacum	1059	-	9	CCATCTTTTT	cis-acting element involved in salicylic acid responsiveness
TCA-element	Brassica oleracea	1247	+	9	CAGAAAAGGA	cis-acting element involved in salicylic acid responsiveness
TCA-element	Brassica oleracea	1238	+	9	GAGAAGAATA	cis-acting element involved in salicylic acid responsiveness

+ WUN-motif

Site Name	Organism	Position	Strand	Matrix score.	sequence	function
WUN-motif	Brassica oleracea	877	-	9	AAATTTCCT	wound-responsive element

Gohir.D09G140400 (*Gossypium hirsutum*)

+ CCGTCC-box

Site Name	Organism	Position	Strand	Matrix score.	sequence	function
CCGTCC-box	Arabidopsis thaliana	81	+	6	CCGTCC	cis-acting regulatory element related to meristem specific activation

+ ERE

Site Name	Organism	Position	Strand	Matrix score.	sequence	function
ERE	Dianthus caryophyllus	802	-	8	ATTTCAAA	ethylene-responsive element

+ HSE

Site Name	Organism	Position	Strand	Matrix score.	sequence	function
HSE	Brassica oleracea	268	+	9	AGAAAATTCG	cis-acting element involved in heat stress responsiveness
HSE	Brassica oleracea	739	+	9	AAAAAATTTC	cis-acting element involved in heat stress responsiveness
HSE	Brassica oleracea	716	+	9	AAAAAATTTC	cis-acting element involved in heat stress responsiveness

+ O2-site

Site Name	Organism	Position	Strand	Matrix score.	sequence	function
O2-site	Zea mays	955	+	9	GATGACATGA	cis-acting regulatory element involved in zein metabolism regulation

+ P-box

Site Name	Organism	Position	Strand	Matrix score.	sequence	function
P-box	Oryza sativa	85	+	7	CCTTTTG	gibberellin-responsive element
P-box	Oryza sativa	865	-	7	CCTTTTG	gibberellin-responsive element

+ Skn-1_motif

Site Name	Organism	Position	Strand	Matrix score.	sequence	function
Skn-1_motif	Oryza sativa	391	+	5	GTCAT	cis-acting regulatory element required for endosperm expression
Skn-1_motif	Oryza sativa	958	+	5	GTCAT	cis-acting regulatory element required for endosperm expression

+ TATC-box

Site Name	Organism	Position	Strand	Matrix score.	sequence	function

TATC-box	Oryza sativa	1153	-	7	TATCCCA	cis-acting element involved in gibberellin-responsiveness

Gorai.006G161000 (*Gossypium raimondii*)

+ CCGTCC-box

Site Name	Organism	Position	Strand	Matrix score.	sequence	function
CCGTCC-box	Arabidopsis thaliana	134	+	6	CCGTCC	cis-acting regulatory element related to meristem specific activation

+ ERE

Site Name	Organism	Position	Strand	Matrix score.	sequence	function
ERE	Dianthus caryophyllus	857	-	8	ATTTCAAA	ethylene-responsive element

+ HSE

Site Name	Organism	Position	Strand	Matrix score.	sequence	function
HSE	Brassica oleracea	321	+	9	AGAAAATTCG	cis-acting element involved in heat stress responsiveness
HSE	Brassica oleracea	771	+	9	AAAAAATTTC	cis-acting element involved in heat stress responsiveness

+ O2-site

Site Name	Organism	Position	Strand	Matrix score.	sequence	function
O2-site	Zea mays	1010	+	9	GATGACATGA	cis-acting regulatory element involved in zein metabolism regulation

+ P-box

Site Name	Organism	Position	Strand	Matrix score.	sequence	function
P-box	Oryza sativa	138	+	7	CCTTTTG	gibberellin-responsive element
P-box	Oryza sativa	920	-	7	CCTTTTG	gibberellin-responsive element

+ Skn-1_motif

Site Name	Organism	Position	Strand	Matrix score.	sequence	function
Skn-1_motif	Oryza sativa	653	-	5	GTCAT	cis-acting regulatory element required for endosperm expression
Skn-1_motif	Oryza sativa	1013	+	5	GTCAT	cis-acting regulatory element required for endosperm expression

+ TCA-element

Site Name	Organism	Position	Strand	Matrix score.	sequence	function
TCA-element	Nicotiana tabacum	36	+	9	CCATCTTTTT	cis-acting element involved in salicylic acid responsiveness

TCA-element	Brassica oleracea	39	-	9	CAGAAAAGGA	cis-acting element involved in salicylic acid responsiveness

Gohir.A09G144600 (*Gossypium hirsutum*)

+ ARE

Site Name	Organism	Position	Strand	Matrix score.	sequence	function
ARE	Zea mays	1268	-	6	TGGTTT	cis-acting regulatory element essential for the anaerobic induction

+ CAT-box

Site Name	Organism	Position	Strand	Matrix score.	sequence	function
CAT-box	Arabidopsis thaliana	106	-	6	GCCACT	cis-acting regulatory element related to meristem expression

+ CGTCA-motif

Site Name	Organism	Position	Strand	Matrix score.	sequence	function
CGTCA-motif	Hordeum vulgare	387	-	5	CGTCA	cis-acting regulatory element involved in the MeJA-responsiveness

+ ELI-box3

Site Name	Organism	Position	Strand	Matrix score.	sequence	function
ELI-box3	Brassica oleracea	1268	+	9	AAACCAATT	elicitor-responsive element

+ ERE

Site Name	Organism	Position	Strand	Matrix score.	sequence	function
ERE	Dianthus caryophyllus	578	-	8	ATTTCAAA	ethylene-responsive element

+ HSE

Site Name	Organism	Position	Strand	Matrix score.	sequence	function
HSE	Brassica oleracea	546	+	9	AAAAAATTTC	cis-acting element involved in heat stress responsiveness

+ O2-site

Site Name	Organism	Position	Strand	Matrix score.	sequence	function
O2-site	Zea mays	726	+	9	GATGACATGA	cis-acting regulatory element involved in zein metabolism regulation
O2-site	Zea mays	1169	-	9	GATGACATGA	cis-acting regulatory element involved in zein metabolism regulation

+ P-box

Site Name	Organism	Position	Strand	Matrix	sequence	function

				score.		
P-box	Oryza sativa	646	-	7	CCTTTTG	gibberellin-responsive element

+ Skn-1_motif

Site Name	Organism	Position	Strand	Matrix score.	sequence	function
Skn-1_motif	Oryza sativa	386	-	5	GTCAT	cis-acting regulatory element required for endosperm expression
Skn-1_motif	Oryza sativa	729	+	5	GTCAT	cis-acting regulatory element required for endosperm expression

+ TATC-box

Site Name	Organism	Position	Strand	Matrix score.	sequence	function
TATC-box	Oryza sativa	1006	-	7	TATCCCA	cis-acting element involved in gibberellin-responsiveness

+ TGACG-motif

Site Name	Organism	Position	Strand	Matrix score.	sequence	function
TGACG-motif	Hordeum vulgare	387	+	5	TGACG	cis-acting regulatory element involved in the MeJA-responsiveness

Thecc1EG022302 (*Theobroma cacao*)

+ ABRE

Site Name	Organism	Position	Strand	Matrix score.	sequence	function
ABRE	Arabidopsis thaliana	711	+	6	CACGTG	cis-acting element involved in the abscisic acid responsiveness

+ ARE

Site Name	Organism	Position	Strand	Matrix score.	sequence	function
ARE	Zea mays	1021	-	6	TGGTTT	cis-acting regulatory element essential for the anaerobic induction

+ Box-W1

Site Name	Organism	Position	Strand	Matrix score.	sequence	function
Box-W1	Petroselinum crispum	274	+	6	TTGACC	fungal elicitor responsive element

+ CAT-box

Site Name	Organism	Position	Strand	Matrix score.	sequence	function
CAT-box	Arabidopsis thaliana	913	-	6	GCCACT	cis-acting regulatory element related to meristem expression

+ ERE

Site Name	Organism	Position	Strand	Matrix score.	sequence	function
ERE	Dianthus caryophyllus	736	-	8	ATTTCAAA	ethylene-responsive element

+ GCN4_motif

Site Name	Organism	Position	Strand	Matrix score.	sequence	function
GCN4_motif	Oryza sativa	883	+	7	TGAGTCA	cis-regulatory element involved in endosperm expression

+ LTR

Site Name	Organism	Position	Strand	Matrix score.	sequence	function
LTR	Hordeum vulgare	72	+	6	CCGAAA	cis-acting element involved in low-temperature responsiveness
LTR	Hordeum vulgare	1281	+	6	CCGAAA	cis-acting element involved in low-temperature responsiveness

+ Skn-1_motif

Site Name	Organism	Position	Strand	Matrix	sequence	function

				score.		
Skn-1_motif	Oryza sativa	1006	+	5	GTCAT	cis-acting regulatory element required for endosperm expression
Skn-1_motif	Oryza sativa	1202	+	5	GTCAT	cis-acting regulatory element required for endosperm expression

+ TC-rich repeats

Site Name	Organism	Position	Strand	Matrix score.	sequence	function
TC-rich repeats	Nicotiana tabacum	491	-	9	ATTTTCTTCA	cis-acting element involved in defense and stress responsiveness

+ TCA-element

Site Name	Organism	Position	Strand	Matrix score.	sequence	function
TCA-element	Nicotiana tabacum	760	-	9	CCATCTTTTT	cis-acting element involved in salicylic acid responsiveness

Kalax.0186s0022 (*Kalanchoe laxiflora*)

+ ARE

Site Name	Organism	Position	Strand	Matrix score.	sequence	function
ARE	Zea mays	1271	-	6	TGGTTT	cis-acting regulatory element essential for the anaerobic induction

+ Box-W1

Site Name	Organism	Position	Strand	Matrix score.	sequence	function
Box-W1	Petroselinum crispum	467	-	6	TTGACC	fungal elicitor responsive element

+ CGTCA-motif

Site Name	Organism	Position	Strand	Matrix score.	sequence	function
CGTCA-motif	Hordeum vulgare	1405	+	5	CGTCA	cis-acting regulatory element involved in the MeJA-responsiveness
CGTCA-motif	Hordeum vulgare	1436	+	5	CGTCA	cis-acting regulatory element involved in the MeJA-responsiveness

+ HSE

Site Name	Organism	Position	Strand	Matrix score.	sequence	function
HSE	Brassica oleracea	415	-	9	AGAAAATTCG	cis-acting element involved in heat stress responsiveness
HSE	Brassica oleracea	473	-	9	AAAAAATTTC	cis-acting element involved in heat stress responsiveness
HSE	Brassica oleracea	427	-	9	AAAAAATTTC	cis-acting element involved in heat stress responsiveness

+ MBS

Site Name	Organism	Position	Strand	Matrix score.	sequence	function
MBS	Arabidopsis thaliana	1325	+	6	CAACTG	MYB binding site involved in drought-inducibility

+ P-box

Site Name	Organism	Position	Strand	Matrix score.	sequence	function
P-box	Oryza sativa	974	-	10	GCCTTTTGAGT	gibberellin-responsive element

+ Skn-1_motif

Site Name	Organism	Position	Strand	Matrix score.	sequence	function
Skn-1_motif	Oryza sativa	488	+	5	GTCAT	cis-acting regulatory element required for

						endosperm expression
Skn-1_motif	Oryza sativa	1406	+	5	GTCAT	cis-acting regulatory element required for endosperm expression
Skn-1_motif	Oryza sativa	589	-	5	GTCAT	cis-acting regulatory element required for endosperm expression
Skn-1_motif	Oryza sativa	1437	+	5	GTCAT	cis-acting regulatory element required for endosperm expression
Skn-1_motif	Oryza sativa	581	+	5	GTCAT	cis-acting regulatory element required for endosperm expression
Skn-1_motif	Oryza sativa	1127	+	5	GTCAT	cis-acting regulatory element required for endosperm expression

+ TC-rich repeats

Site Name	Organism	Position	Strand	Matrix score.	sequence	function
TC-rich repeats	Nicotiana tabacum	418	+	9	ATTTTCTTCA	cis-acting element involved in defense and stress responsiveness
TC-rich repeats	Nicotiana tabacum	1230	-	9	ATTCTCTAAC	cis-acting element involved in defense and stress responsiveness

+ TGA-element

Site Name	Organism	Position	Strand	Matrix score.	sequence	function
TGA-element	Brassica oleracea	1431	+	6	AACGAC	auxin-responsive element

+ TGACG-motif

Site Name	Organism	Position	Strand	Matrix score.	sequence	function
TGACG-motif	Hordeum vulgare	1405	-	5	TGACG	cis-acting regulatory element involved in the MeJA-responsiveness
TGACG-motif	Hordeum vulgare	1436	-	5	TGACG	cis-acting regulatory element involved in the MeJA-responsiveness

Kalax.1083s0001 (*Kalanchoe laxiflora*)

+ ARE

Site Name	Organism	Position	Strand	Matrix score.	sequence	function
ARE	Zea mays	1303	-	6	TGGTTT	cis-acting regulatory element essential for the anaerobic induction

+ Box-W1

Site Name	Organism	Position	Strand	Matrix score.	sequence	function
Box-W1	Petroselinum crispum	501	-	6	TTGACC	fungal elicitor responsive element

+ CGTCA-motif

Site Name	Organism	Position	Strand	Matrix score.	sequence	function
CGTCA-motif	Hordeum vulgare	1437	+	5	CGTCA	cis-acting regulatory element involved in the MeJA-responsiveness
CGTCA-motif	Hordeum vulgare	1468	+	5	CGTCA	cis-acting regulatory element involved in the MeJA-responsiveness

+ GCN4_motif

Site Name	Organism	Position	Strand	Matrix score.	sequence	function
GCN4_motif	Oryza sativa	891	-	7	TGAGTCA	cis-regulatory element involved in endosperm expression

+ HD-Zip 1

Site Name	Organism	Position	Strand	Matrix score.	sequence	function
HD-Zip 1	Arabidopsis thaliana	560	+	8.5	CAAT(A/T)ATTG	element involved in differentiation of the palisade mesophyll cells

+ HD-Zip 2

Site Name	Organism	Position	Strand	Matrix score.	sequence	function
HD-Zip 2	Arabidopsis thaliana	560	+	8	CAAT(G/C)ATTG	element involved in the control of leaf morphology development

+ HSE

Site Name	Organism	Position	Strand	Matrix score.	sequence	function
HSE	Brassica oleracea	449	-	9	AGAAAATTCG	cis-acting element involved in heat stress responsiveness

+ MBS

Site Name	Organism	Position	Strand	Matrix	sequence	function

				score.		
MBS	Arabidopsis thaliana	940	+	6	CAACTG	MYB binding site involved in drought-inducibility

+ Skn-1_motif

Site Name	Organism	Position	Strand	Matrix score.	sequence	function
Skn-1_motif	Oryza sativa	522	+	5	GTCAT	cis-acting regulatory element required for endosperm expression
Skn-1_motif	Oryza sativa	1469	+	5	GTCAT	cis-acting regulatory element required for endosperm expression
Skn-1_motif	Oryza sativa	623	-	5	GTCAT	cis-acting regulatory element required for endosperm expression
Skn-1_motif	Oryza sativa	615	+	5	GTCAT	cis-acting regulatory element required for endosperm expression
Skn-1_motif	Oryza sativa	1438	+	5	GTCAT	cis-acting regulatory element required for endosperm expression

+ TATC-box

Site Name	Organism	Position	Strand	Matrix score.	sequence	function
TATC-box	Oryza sativa	27	+	7	TATCCCA	cis-acting element involved in gibberellin-responsiveness

+ TC-rich repeats

Site Name	Organism	Position	Strand	Matrix score.	sequence	function
TC-rich repeats	Nicotiana tabacum	452	+	9	ATTTTCTTCA	cis-acting element involved in defense and stress responsiveness
TC-rich repeats	Nicotiana tabacum	1262	-	9	ATTCTCTAAC	cis-acting element involved in defense and stress responsiveness
TC-rich repeats	Nicotiana tabacum	839	-	9	GTTTTCTTAC	cis-acting element involved in defense and stress responsiveness

+ TGA-element

Site Name	Organism	Position	Strand	Matrix score.	sequence	function
TGA-element	Brassica oleracea	1463	+	6	AACGAC	auxin-responsive element

+ TGACG-motif

Site Name	Organism	Position	Strand	Matrix score.	sequence	function
TGACG-motif	Hordeum vulgare	1437	-	5	TGACG	cis-acting regulatory element involved in the MeJA-responsiveness

TGACG-motif	Hordeum vulgare	1468	-	5	TGACG	cis-acting regulatory element involved in the MeJA-responsiveness

Kaladp0098s0047 (*Kalanchoe fedtschenkoi*)

+ ARE

Site Name	Organism	Position	Strand	Matrix score.	sequence	function
ARE	Zea mays	1131	+	6	TGGTTT	cis-acting regulatory element essential for the anaerobic induction
ARE	Zea mays	1350	-	6	TGGTTT	cis-acting regulatory element essential for the anaerobic induction
ARE	Zea mays	1304	-	6	TGGTTT	cis-acting regulatory element essential for the anaerobic induction

+ Box-W1

Site Name	Organism	Position	Strand	Matrix score.	sequence	function
Box-W1	Petroselinum crispum	514	-	6	TTGACC	fungal elicitor responsive element

+ CGTCA-motif

Site Name	Organism	Position	Strand	Matrix score.	sequence	function
CGTCA-motif	Hordeum vulgare	983	+	5	CGTCA	cis-acting regulatory element involved in the MeJA-responsiveness
CGTCA-motif	Hordeum vulgare	1469	+	5	CGTCA	cis-acting regulatory element involved in the MeJA-responsiveness
CGTCA-motif	Hordeum vulgare	1438	+	5	CGTCA	cis-acting regulatory element involved in the MeJA-responsiveness

+ HSE

Site Name	Organism	Position	Strand	Matrix score.	sequence	function
HSE	Brassica oleracea	460	-	9	AGAAAATTCG	cis-acting element involved in heat stress responsiveness
HSE	Brassica oleracea	520	-	9	AAAAAATTTC	cis-acting element involved in heat stress responsiveness

+ MBS

Site Name	Organism	Position	Strand	Matrix score.	sequence	function
MBS	Arabidopsis thaliana	954	+	6	CAACTG	MYB binding site involved in drought-inducibility
MBS	Arabidopsis thaliana	1358	+	6	CAACTG	MYB binding site involved in drought-inducibility

+ Skn-1_motif

Site Name	Organism	Position	Strand	Matrix	sequence	function

				score.		
Skn-1_motif	Oryza sativa	535	+	5	GTCAT	cis-acting regulatory element required for endosperm expression
Skn-1_motif	Oryza sativa	1439	+	5	GTCAT	cis-acting regulatory element required for endosperm expression
Skn-1_motif	Oryza sativa	636	-	5	GTCAT	cis-acting regulatory element required for endosperm expression
Skn-1_motif	Oryza sativa	1470	+	5	GTCAT	cis-acting regulatory element required for endosperm expression
Skn-1_motif	Oryza sativa	628	+	5	GTCAT	cis-acting regulatory element required for endosperm expression
Skn-1_motif	Oryza sativa	984	+	5	GTCAT	cis-acting regulatory element required for endosperm expression

+ TC-rich repeats

Site Name	Organism	Position	Strand	Matrix score.	sequence	function
TC-rich repeats	Nicotiana tabacum	463	+	9	ATTTTCTTCA	cis-acting element involved in defense and stress responsiveness
TC-rich repeats	Nicotiana tabacum	1263	-	9	ATTCTCTAAC	cis-acting element involved in defense and stress responsiveness

+ TGA-element

Site Name	Organism	Position	Strand	Matrix score.	sequence	function
TGA-element	Brassica oleracea	1464	+	6	AACGAC	auxin-responsive element

+ TGACG-motif

Site Name	Organism	Position	Strand	Matrix score.	sequence	function
TGACG-motif	Hordeum vulgare	983	-	5	TGACG	cis-acting regulatory element involved in the MeJA-responsiveness
TGACG-motif	Hordeum vulgare	1469	-	5	TGACG	cis-acting regulatory element involved in the MeJA-responsiveness
TGACG-motif	Hordeum vulgare	1438	-	5	TGACG	cis-acting regulatory element involved in the MeJA-responsiveness

Kaladp0011s0266 (*Kalanchoe fedtschenkoi*)

+ ARE

Site Name	Organism	Position	Strand	Matrix score.	sequence	function
ARE	Zea mays	52	-	6	TGGTTT	cis-acting regulatory element essential for the anaerobic induction
ARE	Zea mays	834	-	6	TGGTTT	cis-acting regulatory element essential for the anaerobic induction

+ Box-W1

Site Name	Organism	Position	Strand	Matrix score.	sequence	function
Box-W1	Petroselinum crispum	1425	+	6	TTGACC	fungal elicitor responsive element

+ CAT-box

Site Name	Organism	Position	Strand	Matrix score.	sequence	function
CAT-box	Arabidopsis thaliana	513	+	6	GCCACT	cis-acting regulatory element related to meristem expression

+ CGTCA-motif

Site Name	Organism	Position	Strand	Matrix score.	sequence	function
CGTCA-motif	Hordeum vulgare	384	+	5	CGTCA	cis-acting regulatory element involved in the MeJA-responsiveness

+ HSE

Site Name	Organism	Position	Strand	Matrix score.	sequence	function
HSE	Brassica oleracea	334	-	9	AAAAAATTTC	cis-acting element involved in heat stress responsiveness
HSE	Brassica oleracea	1074	-	9	AAAAAATTTC	cis-acting element involved in heat stress responsiveness
HSE	Brassica oleracea	778	+	10	AAAAAATTTC	cis-acting element involved in heat stress responsiveness

+ O2-site

Site Name	Organism	Position	Strand	Matrix score.	sequence	function
O2-site	Zea mays	622	+	9	GATGACATGA	cis-acting regulatory element involved in zein metabolism regulation
O2-site	Zea mays	818	-	9	GATGACATGA	cis-acting regulatory element involved in zein metabolism regulation
O2-site	Zea mays	686	+	9	GATGACATGA	cis-acting regulatory element involved in

zein metabolism regulation

+ Skn-1_motif

Site Name	Organism	Position	Strand	Matrix score.	sequence	function
Skn-1_motif	Oryza sativa	190	+	5	GTCAT	cis-acting regulatory element required for endosperm expression
Skn-1_motif	Oryza sativa	822	+	5	GTCAT	cis-acting regulatory element required for endosperm expression
Skn-1_motif	Oryza sativa	385	+	5	GTCAT	cis-acting regulatory element required for endosperm expression
Skn-1_motif	Oryza sativa	327	+	5	GTCAT	cis-acting regulatory element required for endosperm expression
Skn-1_motif	Oryza sativa	437	-	5	GTCAT	cis-acting regulatory element required for endosperm expression

+ TC-rich repeats

Site Name	Organism	Position	Strand	Matrix score.	sequence	function
TC-rich repeats	Nicotiana tabacum	337	+	9	ATTCTCTAAC	cis-acting element involved in defense and stress responsiveness
TC-rich repeats	Nicotiana tabacum	825	+	9	ATTTTCTCCA	cis-acting element involved in defense and stress responsiveness

+ TCA-element

Site Name	Organism	Position	Strand	Matrix score.	sequence	function
TCA-element	Brassica oleracea	19	+	9	GAGAAGAATA	cis-acting element involved in salicylic acid responsiveness

+ TGACG-motif

Site Name	Organism	Position	Strand	Matrix score.	sequence	function
TGACG-motif	Hordeum vulgare	384	-	5	TGACG	cis-acting regulatory element involved in the MeJA-responsiveness

+ as-2-box

Site Name	Organism	Position	Strand	Matrix score.	sequence	function
as-2-box	Nicotiana tabacum	430	+	9	GATAatGATG	involved in shoot-specific expression and light responsiveness

Kalax.0178s0076 (*Kalanchoe laxiflora*)

+ ABRE

Site Name	Organism	Position	Strand	Matrix score.	sequence	function
ABRE	Arabidopsis thaliana	766	-	6	CACGTG	cis-acting element involved in the abscisic acid responsiveness

+ ARE

Site Name	Organism	Position	Strand	Matrix score.	sequence	function
ARE	Zea mays	734	-	6	TGGTTT	cis-acting regulatory element essential for the anaerobic induction
ARE	Zea mays	1117	-	6	TGGTTT	cis-acting regulatory element essential for the anaerobic induction

+ CGTCA-motif

Site Name	Organism	Position	Strand	Matrix score.	sequence	function
CGTCA-motif	Hordeum vulgare	464	+	5	CGTCA	cis-acting regulatory element involved in the MeJA-responsiveness

+ GARE-motif

Site Name	Organism	Position	Strand	Matrix score.	sequence	function
GARE-motif	Brassica oleracea	724	+	7	AAACAGA	gibberellin-responsive element

+ HSE

Site Name	Organism	Position	Strand	Matrix score.	sequence	function
HSE	Brassica oleracea	572	-	9	AAAAAATTTC	cis-acting element involved in heat stress responsiveness
HSE	Brassica oleracea	1400	-	9	AAAAAATTTC	cis-acting element involved in heat stress responsiveness

+ MBS

Site Name	Organism	Position	Strand	Matrix score.	sequence	function
MBS	Arabidopsis thaliana	1023	+	6	CAACTG	MYB binding site involved in drought-inducibility

+ O2-site

Site Name	Organism	Position	Strand	Matrix score.	sequence	function
O2-site	Zea mays	237	+	9	GATGACATGA	cis-acting regulatory element involved in zein metabolism regulation

+ Skn-1_motif

Site Name	Organism	Position	Strand	Matrix score.	sequence	function
Skn-1_motif	Oryza sativa	1256	+	5	GTCAT	cis-acting regulatory element required for endosperm expression
Skn-1_motif	Oryza sativa	1393	+	5	GTCAT	cis-acting regulatory element required for endosperm expression

+ TC-rich repeats

Site Name	Organism	Position	Strand	Matrix score.	sequence	function
TC-rich repeats	Nicotiana tabacum	568	-	9	ATTTTCTCCA	cis-acting element involved in defense and stress responsiveness
TC-rich repeats	Nicotiana tabacum	1403	+	9	ATTCTCTAAC	cis-acting element involved in defense and stress responsiveness
TC-rich repeats	Nicotiana tabacum	856	+	9	ATTTTCTTCA	cis-acting element involved in defense and stress responsiveness

+ TCA-element

Site Name	Organism	Position	Strand	Matrix score.	sequence	function
TCA-element	Nicotiana tabacum	754	+	9	CCATCTTTTT	cis-acting element involved in salicylic acid responsiveness
TCA-element	Brassica oleracea	1084	+	9	GAGAAGAATA	cis-acting element involved in salicylic acid responsiveness

+ TGACG-motif

Site Name	Organism	Position	Strand	Matrix score.	sequence	function
TGACG-motif	Hordeum vulgare	464	-	5	TGACG	cis-acting regulatory element involved in the MeJA-responsiveness

Kalax.0420s0030 (*Kalanchoe laxiflora*)

+ ABRE

Site Name	Organism	Position	Strand	Matrix score.	sequence	function
ABRE	Arabidopsis thaliana	14	+	6	CACGTG	cis-acting element involved in the abscisic acid responsiveness

+ ARE

Site Name	Organism	Position	Strand	Matrix score.	sequence	function
ARE	Zea mays	1467	-	6	TGGTTT	cis-acting regulatory element essential for the anaerobic induction

+ Box-W1

Site Name	Organism	Position	Strand	Matrix score.	sequence	function
Box-W1	Petroselinum crispum	1393	+	6	TTGACC	fungal elicitor responsive element

+ HSE

Site Name	Organism	Position	Strand	Matrix score.	sequence	function
HSE	Brassica oleracea	102	-	9	AAAAAATTTC	cis-acting element involved in heat stress responsiveness
HSE	Brassica oleracea	1420	+	9	AAAAAATTTC	cis-acting element involved in heat stress responsiveness
HSE	Brassica oleracea	648	-	9	AAAAAATTTC	cis-acting element involved in heat stress responsiveness
HSE	Brassica oleracea	1421	+	9	AGAAAATTCG	cis-acting element involved in heat stress responsiveness

+ MBS

Site Name	Organism	Position	Strand	Matrix score.	sequence	function
MBS	Arabidopsis thaliana	187	-	6	CAACTG	MYB binding site involved in drought-inducibility
MBS	Arabidopsis thaliana	832	-	6	CAACTG	MYB binding site involved in drought-inducibility

+ Skn-1_motif

Site Name	Organism	Position	Strand	Matrix score.	sequence	function
Skn-1_motif	Oryza sativa	128	+	5	GTCAT	cis-acting regulatory element required for endosperm expression
Skn-1_motif	Oryza sativa	641	+	5	GTCAT	cis-acting regulatory element required for

						endosperm expression
Skn-1_motif	Oryza sativa	504	+	5	GTCAT	cis-acting regulatory element required for endosperm expression
Skn-1_motif	Oryza sativa	751	-	5	GTCAT	cis-acting regulatory element required for endosperm expression

+ TC-rich repeats

Site Name	Organism	Position	Strand	Matrix score.	sequence	function
TC-rich repeats	Nicotiana tabacum	651	+	9	ATTCTCTAAC	cis-acting element involved in defense and stress responsiveness
TC-rich repeats	Nicotiana tabacum	973	+	9	ATTTTCTCCA	cis-acting element involved in defense and stress responsiveness

+ TCA-element

Site Name	Organism	Position	Strand	Matrix score.	sequence	function
TCA-element	Brassica oleracea	332	+	9	GAGAAGAATA	cis-acting element involved in salicylic acid responsiveness

+ WUN-motif

Site Name	Organism	Position	Strand	Matrix score.	sequence	function
WUN-motif	Brassica oleracea	863	-	9	TCATTACGAA	wound-responsive element

Cucsa.306500 (*Cucumis sativus*)

+ ABRE

Site Name	Organism	Position	Strand	Matrix score.	sequence	function
ABRE	Arabidopsis thaliana	1357	-	6	CACGTG	cis-acting element involved in the abscisic acid responsiveness
ABRE	Arabidopsis thaliana	1453	-	7	TACGGTC	cis-acting element involved in the abscisic acid responsiveness

+ ARE

Site Name	Organism	Position	Strand	Matrix score.	sequence	function
ARE	Zea mays	271	+	6	TGGTTT	cis-acting regulatory element essential for the anaerobic induction
ARE	Zea mays	536	+	6	TGGTTT	cis-acting regulatory element essential for the anaerobic induction

+ CGTCA-motif

Site Name	Organism	Position	Strand	Matrix score.	sequence	function
CGTCA-motif	Hordeum vulgare	857	+	5	CGTCA	cis-acting regulatory element involved in the MeJA-responsiveness

+ ERE

Site Name	Organism	Position	Strand	Matrix score.	sequence	function
ERE	Dianthus caryophyllus	435	-	8	ATTTCAAA	ethylene-responsive element

+ GARE-motif

Site Name	Organism	Position	Strand	Matrix score.	sequence	function
GARE-motif	Brassica oleracea	22	+	7	AAACAGA	gibberellin-responsive element
GARE-motif	Brassica oleracea	549	-	7	AAACAGA	gibberellin-responsive element
GARE-motif	Brassica oleracea	457	-	7	AAACAGA	gibberellin-responsive element

+ HD-Zip 1

Site Name	Organism	Position	Strand	Matrix score.	sequence	function
HD-Zip 1	Arabidopsis thaliana	484	+	8.5	CAAT(A/T)ATTG	element involved in differentiation of the palisade mesophyll cells

+ HD-Zip 2

Site Name	Organism	Position	Strand	Matrix score.	sequence	function
HD-Zip 2	Arabidopsis thaliana	484	+	8	CAAT(G/C)ATTG	element involved in the control of leaf morphology development

+ MBS

Site Name	Organism	Position	Strand	Matrix score.	sequence	function
MBS	Arabidopsis thaliana	157	+	6	CAACTG	MYB binding site involved in drought-inducibility

+ O2-site

Site Name	Organism	Position	Strand	Matrix score.	sequence	function
O2-site	Zea mays	656	-	9	GATGACATGA	cis-acting regulatory element involved in zein metabolism regulation
O2-site	Zea mays	1277	+	9	GATGACATGA	cis-acting regulatory element involved in zein metabolism regulation

+ P-box

Site Name	Organism	Position	Strand	Matrix score.	sequence	function
P-box	Oryza sativa	997	-	7	CCTTTTG	gibberellin-responsive element

+ Skn-1_motif

Site Name	Organism	Position	Strand	Matrix score.	sequence	function
Skn-1_motif	Oryza sativa	655	+	5	GTCAT	cis-acting regulatory element required for endosperm expression
Skn-1_motif	Oryza sativa	1278	-	5	GTCAT	cis-acting regulatory element required for endosperm expression
Skn-1_motif	Oryza sativa	658	-	5	GTCAT	cis-acting regulatory element required for endosperm expression

+ TC-rich repeats

Site Name	Organism	Position	Strand	Matrix score.	sequence	function
TC-rich repeats	Nicotiana tabacum	6	+	9	ATTTTCTTCA	cis-acting element involved in defense and stress responsiveness
TC-rich repeats	Nicotiana tabacum	64	-	9	ATTTTCTTCA	cis-acting element involved in defense and stress responsiveness

+ TCA-element

Site Name	Organism	Position	Strand	Matrix score.	sequence	function
TCA-element	Brassica	119	+	10	GAGAAGAATA	cis-acting element involved in salicylic

	oleracea					acid responsiveness
TCA-element	Nicotiana tabacum	940	-	9	CCATCTTTTT	cis-acting element involved in salicylic acid responsiveness
TCA-element	Brassica oleracea	309	+	9	CAGAAAAGGA	cis-acting element involved in salicylic acid responsiveness

+ TGACG-motif

Site Name	Organism	Position	Strand	Matrix score.	sequence	function
TGACG-motif	Hordeum vulgare	857	-	5	TGACG	cis-acting regulatory element involved in the MeJA-responsiveness

+ as-2-box

Site Name	Organism	Position	Strand	Matrix score.	sequence	function
as-2-box	Nicotiana tabacum	1074	+	10	GATAatGATG	involved in shoot-specific expression and light responsiveness

Zosma64g00720 (*Zostera marina*)

+ ABRE

Site Name	Organism	Position	Strand	Matrix score.	sequence	function
ABRE	Arabidopsis thaliana	88	+	6	CACGTG	cis-acting element involved in the abscisic acid responsiveness

+ AuxRR-core

Site Name	Organism	Position	Strand	Matrix score.	sequence	function
AuxRR-core	Nicotiana tabacum	948	+	7	GGTCCAT	cis-acting regulatory element involved in auxin responsiveness

+ CCGTCC-box

Site Name	Organism	Position	Strand	Matrix score.	sequence	function
CCGTCC-box	Arabidopsis thaliana	896	-	6	CCGTCC	cis-acting regulatory element related to meristem specific activation

+ CGTCA-motif

Site Name	Organism	Position	Strand	Matrix score.	sequence	function
CGTCA-motif	Hordeum vulgare	12	-	5	CGTCA	cis-acting regulatory element involved in the MeJA-responsiveness
CGTCA-motif	Hordeum vulgare	85	+	5	CGTCA	cis-acting regulatory element involved in the MeJA-responsiveness
CGTCA-motif	Hordeum vulgare	82	-	5	CGTCA	cis-acting regulatory element involved in the MeJA-responsiveness

+ EIRE

Site Name	Organism	Position	Strand	Matrix score.	sequence	function
EIRE	Nicotiana tabacum	1036	+	7	TTCGACC	elicitor-responsive element

+ GARE-motif

Site Name	Organism	Position	Strand	Matrix score.	sequence	function
GARE-motif	Brassica oleracea	1057	-	7	AAACAGA	gibberellin-responsive element
GARE-motif	Brassica oleracea	1463	+	7	AAACAGA	gibberellin-responsive element

+ GCN4_motif

Site Name	Organism	Position	Strand	Matrix score.	sequence	function

GCN4_motif	Oryza sativa	981	-	7	TGAGTCA	cis-regulatory element involved in endosperm expression

+ LTR

Site Name	Organism	Position	Strand	Matrix score.	sequence	function
LTR	Hordeum vulgare	375	+	6	CCGAAA	cis-acting element involved in low-temperature responsiveness

+ MBS

Site Name	Organism	Position	Strand	Matrix score.	sequence	function
MBS	Arabidopsis thaliana	800	+	6	TAACTG	MYB binding site involved in drought-inducibility

+ O2-site

Site Name	Organism	Position	Strand	Matrix score.	sequence	function
O2-site	Zea mays	80	+	9	GTTGACGTGA	cis-acting regulatory element involved in zein metabolism regulation

+ Skn-1_motif

Site Name	Organism	Position	Strand	Matrix score.	sequence	function
Skn-1_motif	Oryza sativa	11	-	5	GTCAT	cis-acting regulatory element required for endosperm expression
Skn-1_motif	Oryza sativa	1217	+	5	GTCAT	cis-acting regulatory element required for endosperm expression
Skn-1_motif	Oryza sativa	759	-	5	GTCAT	cis-acting regulatory element required for endosperm expression

+ TCA-element

Site Name	Organism	Position	Strand	Matrix score.	sequence	function
TCA-element	Brassica oleracea	49	-	9	GAGAAGAATA	cis-acting element involved in salicylic acid responsiveness
TCA-element	Brassica oleracea	375	+	9	CAGAAAAGGA	cis-acting element involved in salicylic acid responsiveness

+ TGACG-motif

Site Name	Organism	Position	Strand	Matrix score.	sequence	function
TGACG-motif	Hordeum vulgare	12	+	5	TGACG	cis-acting regulatory element involved in the MeJA-responsiveness
TGACG-motif	Hordeum vulgare	85	-	5	TGACG	cis-acting regulatory element involved in the MeJA-responsiveness

TGACG-motif	Hordeum vulgare	82	+	5	TGACG	cis-acting regulatory element involved in the MeJA-responsiveness

+ as1

Site Name	Organism	Position	Strand	Matrix score.	sequence	function
as1	Arabidopsis thaliana	82	+	8	TGACGTCA	cis-acting regulatory element involved in the root-specific expression

GSVIVT01033687001 (*Vitis vinifera*)

+ ARE

Site Name	Organism	Position	Strand	Matrix score.	sequence	function
ARE	Zea mays	106	-	6	TGGTTT	cis-acting regulatory element essential for the anaerobic induction

+ CGTCA-motif

Site Name	Organism	Position	Strand	Matrix score.	sequence	function
CGTCA-motif	Hordeum vulgare	1420	+	5	CGTCA	cis-acting regulatory element involved in the MeJA-responsiveness

+ ERE

Site Name	Organism	Position	Strand	Matrix score.	sequence	function
ERE	Dianthus caryophyllus	616	+	8	ATTTCAAA	ethylene-responsive element

+ GARE-motif

Site Name	Organism	Position	Strand	Matrix score.	sequence	function
GARE-motif	Brassica oleracea	139	+	7	AAACAGA	gibberellin-responsive element
GARE-motif	Brassica oleracea	410	+	7	AAACAGA	gibberellin-responsive element

+ GCN4_motif

Site Name	Organism	Position	Strand	Matrix score.	sequence	function
GCN4_motif	Oryza sativa	685	-	7	TGTGTCA	cis-regulatory element involved in endosperm expression

+ HSE

Site Name	Organism	Position	Strand	Matrix score.	sequence	function
HSE	Brassica oleracea	28	+	9	AAAAAATTTC	cis-acting element involved in heat stress responsiveness
HSE	Brassica oleracea	611	+	9	AAAAAATTTC	cis-acting element involved in heat stress responsiveness
HSE	Brassica oleracea	84	-	9	AAAAAATTTC	cis-acting element involved in heat stress responsiveness

+ Skn-1_motif

Site Name	Organism	Position	Strand	Matrix score.	sequence	function

Skn-1_motif	Oryza sativa	368	+	5	GTCAT	cis-acting regulatory element required for endosperm expression
Skn-1_motif	Oryza sativa	1194	+	5	GTCAT	cis-acting regulatory element required for endosperm expression
Skn-1_motif	Oryza sativa	380	-	5	GTCAT	cis-acting regulatory element required for endosperm expression

+ TCA-element

Site Name	Organism	Position	Strand	Matrix score.	sequence	function
TCA-element	Brassica oleracea	271	-	9	CAGAAAAGGA	cis-acting element involved in salicylic acid responsiveness

+ TGACG-motif

Site Name	Organism	Position	Strand	Matrix score.	sequence	function
TGACG-motif	Hordeum vulgare	1420	-	5	TGACG	cis-acting regulatory element involved in the MeJA-responsiveness

Glyma.15G005900 (*Glycine max*)

+ ARE

Site Name	Organism	Position	Strand	Matrix score.	sequence	function
ARE	Zea mays	433	-	6	TGGTTT	cis-acting regulatory element essential for the anaerobic induction

+ ERE

Site Name	Organism	Position	Strand	Matrix score.	sequence	function
ERE	Dianthus caryophyllus	490	+	8	ATTTCAAA	ethylene-responsive element

+ GARE-motif

Site Name	Organism	Position	Strand	Matrix score.	sequence	function
GARE-motif	Brassica oleracea	335	+	7	AAACAGA	gibberellin-responsive element
GARE-motif	Brassica oleracea	1215	+	7	AAACAGA	gibberellin-responsive element

+ HD-Zip 1

Site Name	Organism	Position	Strand	Matrix score.	sequence	function
HD-Zip 1	Arabidopsis thaliana	144	+	8.5	CAAT(A/T)ATTG	element involved in differentiation of the palisade mesophyll cells

+ HD-Zip 2

Site Name	Organism	Position	Strand	Matrix score.	sequence	function
HD-Zip 2	Arabidopsis thaliana	144	+	8	CAAT(G/C)ATTG	element involved in the control of leaf morphology development

+ HSE

Site Name	Organism	Position	Strand	Matrix score.	sequence	function
HSE	Brassica oleracea	625	-	9	AAAAAATTTC	cis-acting element involved in heat stress responsiveness
HSE	Brassica oleracea	919	+	9	AAAAAATTTC	cis-acting element involved in heat stress responsiveness

+ MBS

Site Name	Organism	Position	Strand	Matrix score.	sequence	function
MBS	Arabidopsis thaliana	194	-	6	TAACTG	MYB binding site involved in drought-inducibility

MBS	Arabidopsis thaliana	1393	+	6	TAACTG	MYB binding site involved in drought-inducibility

+ O2-site

Site Name	Organism	Position	Strand	Matrix score.	sequence	function
O2-site	Zea mays	929	+	9	GATGATATGG	cis-acting regulatory element involved in zein metabolism regulation

+ RY-element

Site Name	Organism	Position	Strand	Matrix score.	sequence	function
RY-element	Helianthus annuus	134	+	8	CATGCATG	cis-acting regulatory element involved in seed-specific regulation

+ Skn-1_motif

Site Name	Organism	Position	Strand	Matrix score.	sequence	function
Skn-1_motif	Oryza sativa	20	-	5	GTCAT	cis-acting regulatory element required for endosperm expression
Skn-1_motif	Oryza sativa	750	-	5	GTCAT	cis-acting regulatory element required for endosperm expression
Skn-1_motif	Oryza sativa	412	-	5	GTCAT	cis-acting regulatory element required for endosperm expression

+ TC-rich repeats

Site Name	Organism	Position	Strand	Matrix score.	sequence	function
TC-rich repeats	Nicotiana tabacum	1201	+	9	ATTTTCTTCA	cis-acting element involved in defense and stress responsiveness

+ TCA-element

Site Name	Organism	Position	Strand	Matrix score.	sequence	function
TCA-element	Brassica oleracea	406	+	9	CAGAAAAGGA	cis-acting element involved in salicylic acid responsiveness

Glyma.13G367400 (*Glycine max*)

+ ABRE

Site Name	Organism	Position	Strand	Matrix score.	sequence	function
ABRE	Hordeum vulgare	310	+	9	CCTACGTGGC	cis-acting element involved in the abscisic acid responsiveness
ABRE	Arabidopsis thaliana	583	-	6	TACGTG	cis-acting element involved in the abscisic acid responsiveness
ABRE	Arabidopsis thaliana	312	+	6	TACGTG	cis-acting element involved in the abscisic acid responsiveness
ABRE	Arabidopsis thaliana	613	+	6	CACGTG	cis-acting element involved in the abscisic acid responsiveness

+ CAT-box

Site Name	Organism	Position	Strand	Matrix score.	sequence	function
CAT-box	Arabidopsis thaliana	296	+	6	GCCACT	cis-acting regulatory element related to meristem expression

+ CGTCA-motif

Site Name	Organism	Position	Strand	Matrix score.	sequence	function
CGTCA-motif	Hordeum vulgare	209	+	5	CGTCA	cis-acting regulatory element involved in the MeJA-responsiveness

+ GARE-motif

Site Name	Organism	Position	Strand	Matrix score.	sequence	function
GARE-motif	Brassica oleracea	571	+	7	AAACAGA	gibberellin-responsive element

+ HSE

Site Name	Organism	Position	Strand	Matrix score.	sequence	function
HSE	Brassica oleracea	259	-	9	AAAAAATTTC	cis-acting element involved in heat stress responsiveness

+ MBS

Site Name	Organism	Position	Strand	Matrix score.	sequence	function
MBS	Arabidopsis thaliana	942	-	6	TAACTG	MYB binding site involved in drought-inducibility
MBS	Arabidopsis thaliana	965	+	6	TAACTG	MYB binding site involved in drought-inducibility
MBS	Arabidopsis	949	-	6	TAACTG	MYB binding site involved in

	thaliana					drought-inducibility

+ Skn-1_motif

Site Name	Organism	Position	Strand	Matrix score.	sequence	function
Skn-1_motif	Oryza sativa	451	+	5	GTCAT	cis-acting regulatory element required for endosperm expression
Skn-1_motif	Oryza sativa	933	+	5	GTCAT	cis-acting regulatory element required for endosperm expression

+ TC-rich repeats

Site Name	Organism	Position	Strand	Matrix score.	sequence	function
TC-rich repeats	Nicotiana tabacum	1072	+	9	ATTTTCTTCA	cis-acting element involved in defense and stress responsiveness
TC-rich repeats	Nicotiana tabacum	1102	+	9	ATTTTCTTCA	cis-acting element involved in defense and stress responsiveness

+ TGACG-motif

Site Name	Organism	Position	Strand	Matrix score.	sequence	function
TGACG-motif	Hordeum vulgare	209	-	5	TGACG	cis-acting regulatory element involved in the MeJA-responsiveness

Phvul.005G180000 (*Phaseolus vulgaris*)

+ ABRE

Site Name	Organism	Position	Strand	Matrix score.	sequence	function
ABRE	Arabidopsis thaliana	109	-	6	TACGTG	cis-acting element involved in the abscisic acid responsiveness
ABRE	Arabidopsis thaliana	762	-	6	TACGTG	cis-acting element involved in the abscisic acid responsiveness

+ ARE

Site Name	Organism	Position	Strand	Matrix score.	sequence	function
ARE	Zea mays	583	-	6	TGGTTT	cis-acting regulatory element essential for the anaerobic induction
ARE	Zea mays	1057	+	6	TGGTTT	cis-acting regulatory element essential for the anaerobic induction
ARE	Zea mays	959	+	6	TGGTTT	cis-acting regulatory element essential for the anaerobic induction

+ Box-W1

Site Name	Organism	Position	Strand	Matrix score.	sequence	function
Box-W1	Petroselinum crispum	660	+	6	TTGACC	fungal elicitor responsive element

+ CGTCA-motif

Site Name	Organism	Position	Strand	Matrix score.	sequence	function
CGTCA-motif	Hordeum vulgare	10	+	5	CGTCA	cis-acting regulatory element involved in the MeJA-responsiveness

+ HSE

Site Name	Organism	Position	Strand	Matrix score.	sequence	function
HSE	Brassica oleracea	1023	+	9	AGAAAATTCG	cis-acting element involved in heat stress responsiveness

+ MBS

Site Name	Organism	Position	Strand	Matrix score.	sequence	function
MBS	Arabidopsis thaliana	171	+	6	TAACTG	MYB binding site involved in drought-inducibility

+ P-box

Site Name	Organism	Position	Strand	Matrix score.	sequence	function

P-box	Oryza sativa	801	-	7	CCTTTTG	gibberellin-responsive element

+ Skn-1_motif

Site Name	Organism	Position	Strand	Matrix score.	sequence	function
Skn-1_motif	Oryza sativa	11	+	5	GTCAT	cis-acting regulatory element required for endosperm expression

+ TC-rich repeats

Site Name	Organism	Position	Strand	Matrix score.	sequence	function
TC-rich repeats	Nicotiana tabacum	316	+	9	ATTTTCTTCA	cis-acting element involved in defense and stress responsiveness
TC-rich repeats	Nicotiana tabacum	1199	+	9	ATTCTCTAAC	cis-acting element involved in defense and stress responsiveness

+ TCA-element

Site Name	Organism	Position	Strand	Matrix score.	sequence	function
TCA-element	Nicotiana tabacum	705	-	9	CCATCTTTTT	cis-acting element involved in salicylic acid responsiveness

+ TGACG-motif

Site Name	Organism	Position	Strand	Matrix score.	sequence	function
TGACG-motif	Hordeum vulgare	10	-	5	TGACG	cis-acting regulatory element involved in the MeJA-responsiveness

Tp57577_TGAC_v2 (*Trifolium pratense*)

+ ARE

Site Name	Organism	Position	Strand	Matrix score.	sequence	function
ARE	Zea mays	651	-	6	TGGTTT	cis-acting regulatory element essential for the anaerobic induction
ARE	Zea mays	1340	+	6	TGGTTT	cis-acting regulatory element essential for the anaerobic induction
ARE	Zea mays	1107	-	6	TGGTTT	cis-acting regulatory element essential for the anaerobic induction

+ AT-rich sequence

Site Name	Organism	Position	Strand	Matrix score.	sequence	function
AT-rich sequence	Pisum sativum	525	-	9	TAAAATACT	element for maximal elicitor-mediated activation (2copies)

+ Box-W1

Site Name	Organism	Position	Strand	Matrix score.	sequence	function
Box-W1	Petroselinum crispum	1251	-	6	TTGACC	fungal elicitor responsive element

+ CGTCA-motif

Site Name	Organism	Position	Strand	Matrix score.	sequence	function
CGTCA-motif	Hordeum vulgare	586	-	5	CGTCA	cis-acting regulatory element involved in the MeJA-responsiveness

+ GCN4_motif

Site Name	Organism	Position	Strand	Matrix score.	sequence	function
GCN4_motif	Oryza sativa	418	-	7	TGTGTCA	cis-regulatory element involved in endosperm expression
GCN4_motif	Oryza sativa	1349	-	7	TGAGTCA	cis-regulatory element involved in endosperm expression
GCN4_motif	Oryza sativa	1299	-	7	TGAGTCA	cis-regulatory element involved in endosperm expression

+ HSE

Site Name	Organism	Position	Strand	Matrix score.	sequence	function
HSE	Brassica oleracea	327	+	9	AAAAAATTTC	cis-acting element involved in heat stress responsiveness

+ Skn-1_motif

Site Name	Organism	Position	Strand	Matrix score.	sequence	function
Skn-1_motif	Oryza sativa	417	-	5	GTCAT	cis-acting regulatory element required for endosperm expression
Skn-1_motif	Oryza sativa	633	+	5	GTCAT	cis-acting regulatory element required for endosperm expression
Skn-1_motif	Oryza sativa	585	-	5	GTCAT	cis-acting regulatory element required for endosperm expression

+ TC-rich repeats

Site Name	Organism	Position	Strand	Matrix score.	sequence	function
TC-rich repeats	Nicotiana tabacum	368	+	9	ATTTTCTCCA	cis-acting element involved in defense and stress responsiveness
TC-rich repeats	Nicotiana tabacum	604	-	9	GTTTTCTTAC	cis-acting element involved in defense and stress responsiveness

+ TGACG-motif

Site Name	Organism	Position	Strand	Matrix score.	sequence	function
TGACG-motif	Hordeum vulgare	586	+	5	TGACG	cis-acting regulatory element involved in the MeJA-responsiveness

Medtr8g009970 (*Medicago truncatula*)

+ ARE

Site Name	Organism	Position	Strand	Matrix score.	sequence	function
ARE	Zea mays	1202	-	6	TGGTTT	cis-acting regulatory element essential for the anaerobic induction

+ CGTCA-motif

Site Name	Organism	Position	Strand	Matrix score.	sequence	function
CGTCA-motif	Hordeum vulgare	1073	-	5	CGTCA	cis-acting regulatory element involved in the MeJA-responsiveness
CGTCA-motif	Hordeum vulgare	1076	+	5	CGTCA	cis-acting regulatory element involved in the MeJA-responsiveness

+ GARE-motif

Site Name	Organism	Position	Strand	Matrix score.	sequence	function
GARE-motif	Brassica oleracea	1176	-	7	TCTGTTG	gibberellin-responsive element

+ HSE

Site Name	Organism	Position	Strand	Matrix score.	sequence	function
HSE	Brassica oleracea	52	-	9	AAAAAATTTC	cis-acting element involved in heat stress responsiveness

+ MBS

Site Name	Organism	Position	Strand	Matrix score.	sequence	function
MBS	Arabidopsis thaliana	1035	+	6	TAACTG	MYB binding site involved in drought-inducibility

+ O2-site

Site Name	Organism	Position	Strand	Matrix score.	sequence	function
O2-site	Zea mays	15	+	9	GATGACATGA	cis-acting regulatory element involved in zein metabolism regulation

+ Skn-1_motif

Site Name	Organism	Position	Strand	Matrix score.	sequence	function
Skn-1_motif	Oryza sativa	16	-	5	GTCAT	cis-acting regulatory element required for endosperm expression
Skn-1_motif	Oryza sativa	1072	-	5	GTCAT	cis-acting regulatory element required for endosperm expression

Skn-1_motif	Oryza sativa	754	-	5	GTCAT	cis-acting regulatory element required for endosperm expression
Skn-1_motif	Oryza sativa	1166	+	5	GTCAT	cis-acting regulatory element required for endosperm expression

+ TC-rich repeats

Site Name	Organism	Position	Strand	Matrix score.	sequence	function
TC-rich repeats	Nicotiana tabacum	471	-	9	ATTTTCTTCA	cis-acting element involved in defense and stress responsiveness
TC-rich repeats	Nicotiana tabacum	1221	-	9	ATTTTCTTCA	cis-acting element involved in defense and stress responsiveness

+ TCA-element

Site Name	Organism	Position	Strand	Matrix score.	sequence	function
TCA-element	Brassica oleracea	875	+	9	GAGAAGAATA	cis-acting element involved in salicylic acid responsiveness

+ TGACG-motif

Site Name	Organism	Position	Strand	Matrix score.	sequence	function
TGACG-motif	Hordeum vulgare	1073	+	5	TGACG	cis-acting regulatory element involved in the MeJA-responsiveness
TGACG-motif	Hordeum vulgare	1076	-	5	TGACG	cis-acting regulatory element involved in the MeJA-responsiveness

+ as1

Site Name	Organism	Position	Strand	Matrix score.	sequence	function
as1	Arabidopsis thaliana	1073	-	8	TGACGTCA	cis-acting regulatory element involved in the root-specific expression

Solyc09g008470.2 (*Solanum lycopersicum*)

+ ARE

Site Name	Organism	Position	Strand	Matrix score.	sequence	function
ARE	Zea mays	317	-	6	TGGTTT	cis-acting regulatory element essential for the anaerobic induction

+ AuxRR-core

Site Name	Organism	Position	Strand	Matrix score.	sequence	function
AuxRR-core	Nicotiana tabacum	51	+	7	GGTCCAT	cis-acting regulatory element involved in auxin responsiveness

+ Box-W1

Site Name	Organism	Position	Strand	Matrix score.	sequence	function
Box-W1	Petroselinum crispum	274	+	6	TTGACC	fungal elicitor responsive element

+ CGTCA-motif

Site Name	Organism	Position	Strand	Matrix score.	sequence	function
CGTCA-motif	Hordeum vulgare	10	-	5	CGTCA	cis-acting regulatory element involved in the MeJA-responsiveness

+ HSE

Site Name	Organism	Position	Strand	Matrix score.	sequence	function
HSE	Brassica oleracea	135	-	9	AAAAAATTTC	cis-acting element involved in heat stress responsiveness
HSE	Brassica oleracea	838	+	9	AAAAAATTTC	cis-acting element involved in heat stress responsiveness
HSE	Brassica oleracea	339	-	9	AAAAAATTTC	cis-acting element involved in heat stress responsiveness

+ Skn-1_motif

Site Name	Organism	Position	Strand	Matrix score.	sequence	function
Skn-1_motif	Oryza sativa	908	+	5	GTCAT	cis-acting regulatory element required for endosperm expression
Skn-1_motif	Oryza sativa	1449	-	5	GTCAT	cis-acting regulatory element required for endosperm expression

+ TC-rich repeats

Site Name	Organism	Position	Strand	Matrix score.	sequence	function

TC-rich repeats	Nicotiana tabacum	1306	+	9	ATTTTCTTCA	cis-acting element involved in defense and stress responsiveness

+ TGACG-motif

Site Name	Organism	Position	Strand	Matrix score.	sequence	function
TGACG-motif	Hordeum vulgare	10	+	5	TGACG	cis-acting regulatory element involved in the MeJA-responsiveness

PGSC0003DMG400003874 (*Solanum tuberosum*)

+ ARE

Site Name	Organism	Position	Strand	Matrix score.	sequence	function
ARE	Zea mays	294	-	6	TGGTTT	cis-acting regulatory element essential for the anaerobic induction

+ Box-W1

Site Name	Organism	Position	Strand	Matrix score.	sequence	function
Box-W1	Petroselinum crispum	337	+	6	TTGACC	fungal elicitor responsive element

+ GARE-motif

Site Name	Organism	Position	Strand	Matrix score.	sequence	function
GARE-motif	Brassica oleracea	727	-	7	AAACAGA	gibberellin-responsive element
GARE-motif	Brassica oleracea	1186	-	7	AAACAGA	gibberellin-responsive element

+ GCN4_motif

Site Name	Organism	Position	Strand	Matrix score.	sequence	function
GCN4_motif	Oryza sativa	484	-	7	TGAGTCA	cis-regulatory element involved in endosperm expression

+ HSE

Site Name	Organism	Position	Strand	Matrix score.	sequence	function
HSE	Brassica oleracea	223	+	9	AAAAAATTTC	cis-acting element involved in heat stress responsiveness
HSE	Brassica oleracea	804	+	9	AAAAAATTTC	cis-acting element involved in heat stress responsiveness
HSE	Brassica oleracea	706	+	9	AAAAAATTTC	cis-acting element involved in heat stress responsiveness
HSE	Brassica oleracea	902	+	9	AAAAAATTTC	cis-acting element involved in heat stress responsiveness

+ P-box

Site Name	Organism	Position	Strand	Matrix score.	sequence	function
P-box	Oryza sativa	140	-	7	CCTTTTG	gibberellin-responsive element
P-box	Oryza sativa	682	+	7	CCTTTTG	gibberellin-responsive element

+ Skn-1_motif

Site Name	Organism	Position	Strand	Matrix score.	sequence	function
Skn-1_motif	Oryza sativa	1193	-	5	GTCAT	cis-acting regulatory element required for endosperm expression

+ TC-rich repeats

Site Name	Organism	Position	Strand	Matrix score.	sequence	function
TC-rich repeats	Nicotiana tabacum	102	+	9	GTTTTCTTAC	cis-acting element involved in defense and stress responsiveness

+ TCA-element

Site Name	Organism	Position	Strand	Matrix score.	sequence	function
TCA-element	Brassica oleracea	721	-	9	CAGAAAAGGA	cis-acting element involved in salicylic acid responsiveness

+ WUN-motif

Site Name	Organism	Position	Strand	Matrix score.	sequence	function
WUN-motif	Brassica oleracea	544	-	9	TCATTACGAA	wound-responsive element

Migut.J00456 (*Mimulus guttatus*)

+ ABRE

Site Name	Organism	Position	Strand	Matrix score.	sequence	function
ABRE	Arabidopsis thaliana	522	+	7	TACGGTC	cis-acting element involved in the abscisic acid responsiveness

+ ARE

Site Name	Organism	Position	Strand	Matrix score.	sequence	function
ARE	Zea mays	709	+	6	TGGTTT	cis-acting regulatory element essential for the anaerobic induction
ARE	Zea mays	925	+	6	TGGTTT	cis-acting regulatory element essential for the anaerobic induction

+ Box-W1

Site Name	Organism	Position	Strand	Matrix score.	sequence	function
Box-W1	Petroselinum crispum	1323	-	6	TTGACC	fungal elicitor responsive element

+ CGTCA-motif

Site Name	Organism	Position	Strand	Matrix score.	sequence	function
CGTCA-motif	Hordeum vulgare	902	-	5	CGTCA	cis-acting regulatory element involved in the MeJA-responsiveness

+ ELI-box3

Site Name	Organism	Position	Strand	Matrix score.	sequence	function
ELI-box3	Brassica oleracea	706	-	9	AAACCAATT	elicitor-responsive element

+ ERE

Site Name	Organism	Position	Strand	Matrix score.	sequence	function
ERE	Dianthus caryophyllus	292	-	8	ATTTCAAA	ethylene-responsive element

+ HSE

Site Name	Organism	Position	Strand	Matrix score.	sequence	function
HSE	Brassica oleracea	274	+	9	AAAAAATTTC	cis-acting element involved in heat stress responsiveness

+ LTR

Site Name	Organism	Position	Strand	Matrix	sequence	function

				score.		
LTR	Hordeum vulgare	25	+	6	CCGAAA	cis-acting element involved in low-temperature responsiveness
LTR	Hordeum vulgare	339	+	6	CCGAAA	cis-acting element involved in low-temperature responsiveness
LTR	Hordeum vulgare	280	-	6	CCGAAA	cis-acting element involved in low-temperature responsiveness
LTR	Hordeum vulgare	434	+	6	CCGAAA	cis-acting element involved in low-temperature responsiveness
LTR	Hordeum vulgare	46	+	6	CCGAAA	cis-acting element involved in low-temperature responsiveness
LTR	Hordeum vulgare	372	+	6	CCGAAA	cis-acting element involved in low-temperature responsiveness
LTR	Hordeum vulgare	331	+	6	CCGAAA	cis-acting element involved in low-temperature responsiveness
LTR	Hordeum vulgare	608	+	6	CCGAAA	cis-acting element involved in low-temperature responsiveness

+ P-box

Site Name	Organism	Position	Strand	Matrix score.	sequence	function
P-box	Oryza sativa	353	+	7	CCTTTTG	gibberellin-responsive element
P-box	Oryza sativa	1326	-	7	CCTTTTG	gibberellin-responsive element

+ TCA-element

Site Name	Organism	Position	Strand	Matrix score.	sequence	function
TCA-element	Nicotiana tabacum	1193	-	9	CCATCTTTTT	cis-acting element involved in salicylic acid responsiveness
TCA-element	Nicotiana tabacum	1217	-	9	CCATCTTTTT	cis-acting element involved in salicylic acid responsiveness

+ TGACG-motif

Site Name	Organism	Position	Strand	Matrix score.	sequence	function
TGACG-motif	Hordeum vulgare	902	+	5	TGACG	cis-acting regulatory element involved in the MeJA-responsiveness

DCAR_020804 (*Daucus carota*)

+ ABRE

Site Name	Organism	Position	Strand	Matrix score.	sequence	function
ABRE	Arabidopsis thaliana	147	+	6	CACGTG	cis-acting element involved in the abscisic acid responsiveness

+ AT-rich sequence

Site Name	Organism	Position	Strand	Matrix score.	sequence	function
AT-rich sequence	Pisum sativum	1043	+	9	TAAAATACT	element for maximal elicitor-mediated activation (2copies)

+ Box-W1

Site Name	Organism	Position	Strand	Matrix score.	sequence	function
Box-W1	Petroselinum crispum	1211	+	6	TTGACC	fungal elicitor responsive element

+ GCN4_motif

Site Name	Organism	Position	Strand	Matrix score.	sequence	function
GCN4_motif	Oryza sativa	853	+	7	TGAGTCA	cis-regulatory element involved in endosperm expression

+ HSE

Site Name	Organism	Position	Strand	Matrix score.	sequence	function
HSE	Brassica oleracea	935	+	9	AAAAAATTTC	cis-acting element involved in heat stress responsiveness

+ LTR

Site Name	Organism	Position	Strand	Matrix score.	sequence	function
LTR	Hordeum vulgare	1334	-	6	CCGAAA	cis-acting element involved in low-temperature responsiveness

+ O2-site

Site Name	Organism	Position	Strand	Matrix score.	sequence	function
O2-site	Zea mays	1127	+	9	GATGATGTGG	cis-acting regulatory element involved in zein metabolism regulation

+ Skn-1_motif

Site Name	Organism	Position	Strand	Matrix score.	sequence	function
Skn-1_motif	Oryza sativa	870	+	5	GTCAT	cis-acting regulatory element required for

endosperm expression

+ TC-rich repeats

Site Name	Organism	Position	Strand	Matrix score.	sequence	function
TC-rich repeats	Nicotiana tabacum	680	+	9	ATTTTCTTCA	cis-acting element involved in defense and stress responsiveness

Aqcoe2G212900 (*Aquilegia coerulea*)

+ ARE

Site Name	Organism	Position	Strand	Matrix score.	sequence	function
ARE	Zea mays	597	+	6	TGGTTT	cis-acting regulatory element essential for the anaerobic induction

+ AuxRR-core

Site Name	Organism	Position	Strand	Matrix score.	sequence	function
AuxRR-core	Nicotiana tabacum	1401	-	7	GGTCCAT	cis-acting regulatory element involved in auxin responsiveness

+ CAT-box

Site Name	Organism	Position	Strand	Matrix score.	sequence	function
CAT-box	Arabidopsis thaliana	1108	+	6	GCCACT	cis-acting regulatory element related to meristem expression

+ GARE-motif

Site Name	Organism	Position	Strand	Matrix score.	sequence	function
GARE-motif	Brassica oleracea	946	-	7	AAACAGA	gibberellin-responsive element
GARE-motif	Brassica oleracea	952	-	7	AAACAGA	gibberellin-responsive element

+ GCN4_motif

Site Name	Organism	Position	Strand	Matrix score.	sequence	function
GCN4_motif	Oryza sativa	36	-	7	TGAGTCA	cis-regulatory element involved in endosperm expression
GCN4_motif	Oryza sativa	211	+	7	TGTGTCA	cis-regulatory element involved in endosperm expression

+ HSE

Site Name	Organism	Position	Strand	Matrix score.	sequence	function
HSE	Brassica oleracea	391	+	9	AAAAAATTTC	cis-acting element involved in heat stress responsiveness

+ LTR

Site Name	Organism	Position	Strand	Matrix score.	sequence	function
LTR	Hordeum vulgare	1325	+	6	CCGAAA	cis-acting element involved in low-temperature responsiveness

+ MBS

Site Name	Organism	Position	Strand	Matrix score.	sequence	function
MBS	Arabidopsis thaliana	344	+	6	TAACTG	MYB binding site involved in drought-inducibility
MBS	Arabidopsis thaliana	593	+	6	TAACTG	MYB binding site involved in drought-inducibility
MBS	Arabidopsis thaliana	382	+	6	TAACTG	MYB binding site involved in drought-inducibility

+ O2-site

Site Name	Organism	Position	Strand	Matrix score.	sequence	function
O2-site	Zea mays	1381	+	9	GATGATATGG	cis-acting regulatory element involved in zein metabolism regulation

+ P-box

Site Name	Organism	Position	Strand	Matrix score.	sequence	function
P-box	Oryza sativa	1019	-	7	CCTTTTG	gibberellin-responsive element
P-box	Petroselinum crispum	1117	-	13	TTCCAACAAACCCC	gibberellin-responsive element and part of a light responsive element

+ Skn-1_motif

Site Name	Organism	Position	Strand	Matrix score.	sequence	function
Skn-1_motif	Oryza sativa	35	-	5	GTCAT	cis-acting regulatory element required for endosperm expression
Skn-1_motif	Oryza sativa	476	-	5	GTCAT	cis-acting regulatory element required for endosperm expression
Skn-1_motif	Oryza sativa	214	+	5	GTCAT	cis-acting regulatory element required for endosperm expression

+ TCA-element

Site Name	Organism	Position	Strand	Matrix score.	sequence	function
TCA-element	Brassica oleracea	814	+	9	GAGAAGAATA	cis-acting element involved in salicylic acid responsiveness
TCA-element	Brassica oleracea	1239	-	9	GAGAAGAATA	cis-acting element involved in salicylic acid responsiveness
TCA-element	Brassica oleracea	1153	+	9	GAGAAGAATA	cis-acting element involved in salicylic acid responsiveness

MDP0000286077 (*Malus domestica*)

+ ABRE

Site Name	Organism	Position	Strand	Matrix score.	sequence	function
ABRE	Arabidopsis thaliana	837	-	6	CACGTG	cis-acting element involved in the abscisic acid responsiveness
ABRE	Arabidopsis thaliana	1100	-	6	CACGTG	cis-acting element involved in the abscisic acid responsiveness

+ Box-W1

Site Name	Organism	Position	Strand	Matrix score.	sequence	function
Box-W1	Petroselinum crispum	171	+	6	TTGACC	fungal elicitor responsive element

+ CAT-box

Site Name	Organism	Position	Strand	Matrix score.	sequence	function
CAT-box	Arabidopsis thaliana	1136	+	6	GCCACT	cis-acting regulatory element related to meristem expression

+ CCGTCC-box

Site Name	Organism	Position	Strand	Matrix score.	sequence	function
CCGTCC-box	Arabidopsis thaliana	1260	-	6	CCGTCC	cis-acting regulatory element related to meristem specific activation

+ CGTCA-motif

Site Name	Organism	Position	Strand	Matrix score.	sequence	function
CGTCA-motif	Hordeum vulgare	227	+	5	CGTCA	cis-acting regulatory element involved in the MeJA-responsiveness
CGTCA-motif	Hordeum vulgare	1239	+	5	CGTCA	cis-acting regulatory element involved in the MeJA-responsiveness
CGTCA-motif	Hordeum vulgare	496	+	5	CGTCA	cis-acting regulatory element involved in the MeJA-responsiveness
CGTCA-motif	Hordeum vulgare	369	+	5	CGTCA	cis-acting regulatory element involved in the MeJA-responsiveness
CGTCA-motif	Hordeum vulgare	505	-	5	CGTCA	cis-acting regulatory element involved in the MeJA-responsiveness

+ HSE

Site Name	Organism	Position	Strand	Matrix score.	sequence	function
HSE	Brassica	344	+	9	AAAAAATTTC	cis-acting element involved in heat stress

	oleracea					responsiveness
HSE	Brassica oleracea	570	-	9	AAAAAATTTC	cis-acting element involved in heat stress responsiveness

+ LTR

Site Name	Organism	Position	Strand	Matrix score.	sequence	function
LTR	Hordeum vulgare	559	+	6	CCGAAA	cis-acting element involved in low-temperature responsiveness

+ MBS

Site Name	Organism	Position	Strand	Matrix score.	sequence	function
MBS	Arabidopsis thaliana	1489	+	6	CAACTG	MYB binding site involved in drought-inducibility

+ O2-site

Site Name	Organism	Position	Strand	Matrix score.	sequence	function
O2-site	Zea mays	1236	-	9	GTTGACGTGA	cis-acting regulatory element involved in zein metabolism regulation
O2-site	Zea mays	1308	-	9	GATGACATGA	cis-acting regulatory element involved in zein metabolism regulation

+ Skn-1_motif

Site Name	Organism	Position	Strand	Matrix score.	sequence	function
Skn-1_motif	Oryza sativa	150	+	5	GTCAT	cis-acting regulatory element required for endosperm expression
Skn-1_motif	Oryza sativa	519	+	5	GTCAT	cis-acting regulatory element required for endosperm expression
Skn-1_motif	Oryza sativa	228	+	5	GTCAT	cis-acting regulatory element required for endosperm expression
Skn-1_motif	Oryza sativa	1240	+	5	GTCAT	cis-acting regulatory element required for endosperm expression

+ TCA-element

Site Name	Organism	Position	Strand	Matrix score.	sequence	function
TCA-element	Nicotiana tabacum	332	+	9	CCATCTTTTT	cis-acting element involved in salicylic acid responsiveness

+ TGACG-motif

Site Name	Organism	Position	Strand	Matrix score.	sequence	function
TGACG-motif	Hordeum	227	-	5	TGACG	cis-acting regulatory element involved in the

	vulgare					MeJA-responsiveness
TGACG-motif	Hordeum vulgare	1239	-	5	TGACG	cis-acting regulatory element involved in the MeJA-responsiveness
TGACG-motif	Hordeum vulgare	496	-	5	TGACG	cis-acting regulatory element involved in the MeJA-responsiveness
TGACG-motif	Hordeum vulgare	369	-	5	TGACG	cis-acting regulatory element involved in the MeJA-responsiveness
TGACG-motif	Hordeum vulgare	505	+	5	TGACG	cis-acting regulatory element involved in the MeJA-responsiveness

+ motif IIb

Site Name	Organism	Position	Strand	Matrix score.	sequence	function
motif IIb	Oryza sativa	1051	-	9	CCGCCGCGCT	abscisic acid responsive element

MDP0000534942 (*Malus domestica*)

+ ABRE

Site Name	Organism	Position	Strand	Matrix score.	sequence	function
ABRE	Triticum aestivum	651	-	9	GACACGTGGC	cis-acting element involved in the abscisic acid responsiveness
ABRE	Arabidopsis thaliana	1238	-	7	TACGGTC	cis-acting element involved in the abscisic acid responsiveness

+ ARE

Site Name	Organism	Position	Strand	Matrix score.	sequence	function
ARE	Zea mays	532	-	6	TGGTTT	cis-acting regulatory element essential for the anaerobic induction

+ Box-W1

Site Name	Organism	Position	Strand	Matrix score.	sequence	function
Box-W1	Petroselinum crispum	99	-	6	TTGACC	fungal elicitor responsive element
Box-W1	Petroselinum crispum	435	-	6	TTGACC	fungal elicitor responsive element

+ CGTCA-motif

Site Name	Organism	Position	Strand	Matrix score.	sequence	function
CGTCA-motif	Hordeum vulgare	844	-	5	CGTCA	cis-acting regulatory element involved in the MeJA-responsiveness

+ EIRE

Site Name	Organism	Position	Strand	Matrix score.	sequence	function
EIRE	Nicotiana tabacum	829	-	7	TTCGACC	elicitor-responsive element

+ GCN4_motif

Site Name	Organism	Position	Strand	Matrix score.	sequence	function
GCN4_motif	Oryza sativa	400	-	7	TGTGTCA	cis-regulatory element involved in endosperm expression

+ MBS

Site Name	Organism	Position	Strand	Matrix score.	sequence	function
MBS	Arabidopsis thaliana	1328	+	6	CAACTG	MYB binding site involved in drought-inducibility

MBS	Arabidopsis thaliana	1489	+	6	CAACTG	MYB binding site involved in drought-inducibility

+ O2-site

Site Name	Organism	Position	Strand	Matrix score.	sequence	function
O2-site	Zea mays	1254	+	9	GATGACATGA	cis-acting regulatory element involved in zein metabolism regulation

+ P-box

Site Name	Organism	Position	Strand	Matrix score.	sequence	function
P-box	Oryza sativa	300	+	7	CCTTTTG	gibberellin-responsive element
P-box	Oryza sativa	1028	-	7	CCTTTTG	gibberellin-responsive element

+ Skn-1_motif

Site Name	Organism	Position	Strand	Matrix score.	sequence	function
Skn-1_motif	Oryza sativa	110	-	5	GTCAT	cis-acting regulatory element required for endosperm expression
Skn-1_motif	Oryza sativa	1255	-	5	GTCAT	cis-acting regulatory element required for endosperm expression

+ TC-rich repeats

Site Name	Organism	Position	Strand	Matrix score.	sequence	function
TC-rich repeats	Nicotiana tabacum	1442	+	9	ATTTTCTCCA	cis-acting element involved in defense and stress responsiveness

+ TCA-element

Site Name	Organism	Position	Strand	Matrix score.	sequence	function
TCA-element	Brassica oleracea	895	-	10	GAGAAGAATA	cis-acting element involved in salicylic acid responsiveness

+ TGACG-motif

Site Name	Organism	Position	Strand	Matrix score.	sequence	function
TGACG-motif	Hordeum vulgare	844	+	5	TGACG	cis-acting regulatory element involved in the MeJA-responsiveness

Prupe.7G125300 (*Prunus persica*)

+ ARE

Site Name	Organism	Position	Strand	Matrix score.	sequence	function
ARE	Zea mays	624	+	6	TGGTTT	cis-acting regulatory element essential for the anaerobic induction
ARE	Zea mays	812	-	6	TGGTTT	cis-acting regulatory element essential for the anaerobic induction
ARE	Zea mays	795	-	6	TGGTTT	cis-acting regulatory element essential for the anaerobic induction
ARE	Zea mays	1223	-	6	TGGTTT	cis-acting regulatory element essential for the anaerobic induction

+ CGTCA-motif

Site Name	Organism	Position	Strand	Matrix score.	sequence	function
CGTCA-motif	Hordeum vulgare	296	+	5	CGTCA	cis-acting regulatory element involved in the MeJA-responsiveness

+ GARE-motif

Site Name	Organism	Position	Strand	Matrix score.	sequence	function
GARE-motif	Brassica oleracea	146	-	7	AAACAGA	gibberellin-responsive element
GARE-motif	Brassica oleracea	210	+	7	TCTGTTG	gibberellin-responsive element

+ MBS

Site Name	Organism	Position	Strand	Matrix score.	sequence	function
MBS	Arabidopsis thaliana	556	+	6	TAACTG	MYB binding site involved in drought-inducibility
MBS	Arabidopsis thaliana	1448	+	6	CAACTG	MYB binding site involved in drought-inducibility

+ Skn-1_motif

Site Name	Organism	Position	Strand	Matrix score.	sequence	function
Skn-1_motif	Oryza sativa	498	-	5	GTCAT	cis-acting regulatory element required for endosperm expression
Skn-1_motif	Oryza sativa	766	-	5	GTCAT	cis-acting regulatory element required for endosperm expression

+ TGACG-motif

Site Name	Organism	Position	Strand	Matrix	sequence	function

				score.		
TGACG-motif	Hordeum vulgare	296	-	5	TGACG	cis-acting regulatory element involved in the MeJA-responsiveness

+ █ as-2-box

Site Name	Organism	Position	Strand	Matrix score.	sequence	function
as-2-box	Nicotiana tabacum	432	+	9	GATAatGATG	involved in shoot-specific expression and light responsiveness

Prupe.4G071900.1.p (*Prunus persica*)

+ ABRE

Site Name	Organism	Position	Strand	Matrix score.	sequence	function
ABRE	Arabidopsis thaliana	306	+	6	CACGTG	cis-acting element involved in the abscisic acid responsiveness

+ ARE

Site Name	Organism	Position	Strand	Matrix score.	sequence	function
ARE	Zea mays	35	+	6	TGGTTT	cis-acting regulatory element essential for the anaerobic induction
ARE	Zea mays	1272	-	6	TGGTTT	cis-acting regulatory element essential for the anaerobic induction
ARE	Zea mays	1027	-	6	TGGTTT	cis-acting regulatory element essential for the anaerobic induction

+ Box-W1

Site Name	Organism	Position	Strand	Matrix score.	sequence	function
Box-W1	Petroselinum crispum	529	+	6	TTGACC	fungal elicitor responsive element

+ CGTCA-motif

Site Name	Organism	Position	Strand	Matrix score.	sequence	function
CGTCA-motif	Hordeum vulgare	101	+	5	CGTCA	cis-acting regulatory element involved in the MeJA-responsiveness
CGTCA-motif	Hordeum vulgare	1457	+	5	CGTCA	cis-acting regulatory element involved in the MeJA-responsiveness
CGTCA-motif	Hordeum vulgare	636	+	5	CGTCA	cis-acting regulatory element involved in the MeJA-responsiveness

+ MBS

Site Name	Organism	Position	Strand	Matrix score.	sequence	function
MBS	Arabidopsis thaliana	206	+	6	CAACTG	MYB binding site involved in drought-inducibility
MBS	Arabidopsis thaliana	1066	+	6	CAACTG	MYB binding site involved in drought-inducibility

+ O2-site

Site Name	Organism	Position	Strand	Matrix score.	sequence	function
O2-site	Zea mays	618	-	9	GATGATGTGG	cis-acting regulatory element involved in

zein metabolism regulation

+ Skn-1_motif

Site Name	Organism	Position	Strand	Matrix score.	sequence	function
Skn-1_motif	Oryza sativa	94	+	5	GTCAT	cis-acting regulatory element required for endosperm expression
Skn-1_motif	Oryza sativa	503	-	5	GTCAT	cis-acting regulatory element required for endosperm expression
Skn-1_motif	Oryza sativa	382	-	5	GTCAT	cis-acting regulatory element required for endosperm expression

+ TC-rich repeats

Site Name	Organism	Position	Strand	Matrix score.	sequence	function
TC-rich repeats	Nicotiana tabacum	418	+	9	ATTTTCTTCA	cis-acting element involved in defense and stress responsiveness

+ TGACG-motif

Site Name	Organism	Position	Strand	Matrix score.	sequence	function
TGACG-motif	Hordeum vulgare	101	-	5	TGACG	cis-acting regulatory element involved in the MeJA-responsiveness
TGACG-motif	Hordeum vulgare	1457	-	5	TGACG	cis-acting regulatory element involved in the MeJA-responsiveness
TGACG-motif	Hordeum vulgare	636	-	5	TGACG	cis-acting regulatory element involved in the MeJA-responsiveness

+ as-2-box

Site Name	Organism	Position	Strand	Matrix score.	sequence	function
as-2-box	Nicotiana tabacum	1211	-	9	GATAatGATG	involved in shoot-specific expression and light responsiveness

mrna09805.1-v1.0-hybrid (*Fragaria vesca*)

+ ARE

Site Name	Organism	Position	Strand	Matrix score.	sequence	function
ARE	Zea mays	1309	-	6	TGGTTT	cis-acting regulatory element essential for the anaerobic induction
ARE	Zea mays	1404	-	6	TGGTTT	cis-acting regulatory element essential for the anaerobic induction

+ AuxRR-core

Site Name	Organism	Position	Strand	Matrix score.	sequence	function
AuxRR-core	Nicotiana tabacum	6	-	7	GGTCCAT	cis-acting regulatory element involved in auxin responsiveness
AuxRR-core	Nicotiana tabacum	1181	-	7	GGTCCAT	cis-acting regulatory element involved in auxin responsiveness

+ GARE-motif

Site Name	Organism	Position	Strand	Matrix score.	sequence	function
GARE-motif	Brassica oleracea	105	-	7	TCTGTTG	gibberellin-responsive element
GARE-motif	Brassica oleracea	1280	-	7	AAACAGA	gibberellin-responsive element
GARE-motif	Brassica oleracea	1255	-	7	TCTGTTG	gibberellin-responsive element

+ LTR

Site Name	Organism	Position	Strand	Matrix score.	sequence	function
LTR	Hordeum vulgare	1273	+	6	CCGAAA	cis-acting element involved in low-temperature responsiveness

+ MBS

Site Name	Organism	Position	Strand	Matrix score.	sequence	function
MBS	Arabidopsis thaliana	755	-	6	TAACTG	MYB binding site involved in drought-inducibility
MBS	Arabidopsis thaliana	1372	+	6	TAACTG	MYB binding site involved in drought-inducibility

+ O2-site

Site Name	Organism	Position	Strand	Matrix score.	sequence	function
O2-site	Zea mays	488	+	9	GATGACATGA	cis-acting regulatory element involved in

						zein metabolism regulation
O2-site	Zea mays	493	+	9	GATGACATGA	cis-acting regulatory element involved in zein metabolism regulation

+ P-box

Site Name	Organism	Position	Strand	Matrix score.	sequence	function
P-box	Oryza sativa	337	-	7	CCTTTTG	gibberellin-responsive element

+ Skn-1_motif

Site Name	Organism	Position	Strand	Matrix score.	sequence	function
Skn-1_motif	Oryza sativa	25	-	5	GTCAT	cis-acting regulatory element required for endosperm expression
Skn-1_motif	Oryza sativa	1147	+	5	GTCAT	cis-acting regulatory element required for endosperm expression
Skn-1_motif	Oryza sativa	1014	-	5	GTCAT	cis-acting regulatory element required for endosperm expression

+ as-2-box

Site Name	Organism	Position	Strand	Matrix score.	sequence	function
as-2-box	Nicotiana tabacum	1294	-	9	GATAatGATG	involved in shoot-specific expression and light responsiveness

Potri.006G121300 (*Populus trichocarpa*)

+ ABRE

Site Name	Organism	Position	Strand	Matrix score.	sequence	function
ABRE	Arabidopsis thaliana	383	+	6	CACGTG	cis-acting element involved in the abscisic acid responsiveness

+ ARE

Site Name	Organism	Position	Strand	Matrix score.	sequence	function
ARE	Zea mays	823	-	6	TGGTTT	cis-acting regulatory element essential for the anaerobic induction

+ Box-W1

Site Name	Organism	Position	Strand	Matrix score.	sequence	function
Box-W1	Petroselinum crispum	678	-	6	TTGACC	fungal elicitor responsive element

+ CAT-box

Site Name	Organism	Position	Strand	Matrix score.	sequence	function
CAT-box	Arabidopsis thaliana	1034	+	6	GCCACT	cis-acting regulatory element related to meristem expression

+ CGTCA-motif

Site Name	Organism	Position	Strand	Matrix score.	sequence	function
CGTCA-motif	Hordeum vulgare	1063	+	5	CGTCA	cis-acting regulatory element involved in the MeJA-responsiveness

+ EIRE

Site Name	Organism	Position	Strand	Matrix score.	sequence	function
EIRE	Nicotiana tabacum	889	-	7	TTCGACC	elicitor-responsive element

+ GCN4_motif

Site Name	Organism	Position	Strand	Matrix score.	sequence	function
GCN4_motif	Oryza sativa	87	+	7	TGAGTCA	cis-regulatory element involved in endosperm expression
GCN4_motif	Oryza sativa	472	+	7	TGTGTCA	cis-regulatory element involved in endosperm expression

+ HSE

Site Name	Organism	Position	Strand	Matrix	sequence	function

				score.		
HSE	Brassica oleracea	634	+	9	AAAAAATTTC	cis-acting element involved in heat stress responsiveness

+ P-box

Site Name	Organism	Position	Strand	Matrix score.	sequence	function
P-box	Oryza sativa	1231	-	7	CCTTTTG	gibberellin-responsive element

+ Skn-1_motif

Site Name	Organism	Position	Strand	Matrix score.	sequence	function
Skn-1_motif	Oryza sativa	39	-	5	GTCAT	cis-acting regulatory element required for endosperm expression
Skn-1_motif	Oryza sativa	90	+	5	GTCAT	cis-acting regulatory element required for endosperm expression
Skn-1_motif	Oryza sativa	55	-	5	GTCAT	cis-acting regulatory element required for endosperm expression
Skn-1_motif	Oryza sativa	475	+	5	GTCAT	cis-acting regulatory element required for endosperm expression

+ TATC-box

Site Name	Organism	Position	Strand	Matrix score.	sequence	function
TATC-box	Oryza sativa	244	-	7	TATCCCA	cis-acting element involved in gibberellin-responsiveness
TATC-box	Oryza sativa	1054	+	7	TATCCCA	cis-acting element involved in gibberellin-responsiveness

+ TGACG-motif

Site Name	Organism	Position	Strand	Matrix score.	sequence	function
TGACG-motif	Hordeum vulgare	1063	-	5	TGACG	cis-acting regulatory element involved in the MeJA-responsiveness

Podel.06G130900 (*Populus deltoides*)

+ ARE

Site Name	Organism	Position	Strand	Matrix score.	sequence	function
ARE	Zea mays	280	-	6	TGGTTT	cis-acting regulatory element essential for the anaerobic induction

+ CAT-box

Site Name	Organism	Position	Strand	Matrix score.	sequence	function
CAT-box	Arabidopsis thaliana	991	+	6	GCCACT	cis-acting regulatory element related to meristem expression

+ CGTCA-motif

Site Name	Organism	Position	Strand	Matrix score.	sequence	function
CGTCA-motif	Hordeum vulgare	163	+	5	CGTCA	cis-acting regulatory element involved in the MeJA-responsiveness
CGTCA-motif	Hordeum vulgare	1097	-	5	CGTCA	cis-acting regulatory element involved in the MeJA-responsiveness
CGTCA-motif	Hordeum vulgare	1020	+	5	CGTCA	cis-acting regulatory element involved in the MeJA-responsiveness

+ GCN4_motif

Site Name	Organism	Position	Strand	Matrix score.	sequence	function
GCN4_motif	Oryza sativa	37	+	7	TGAGTCA	cis-regulatory element involved in endosperm expression

+ HSE

Site Name	Organism	Position	Strand	Matrix score.	sequence	function
HSE	Brassica oleracea	592	+	9	AAAAAATTTC	cis-acting element involved in heat stress responsiveness
HSE	Brassica oleracea	1231	+	9	AAAAAATTTC	cis-acting element involved in heat stress responsiveness
HSE	Brassica oleracea	848	+	9	AAAAAATTTC	cis-acting element involved in heat stress responsiveness

+ Skn-1_motif

Site Name	Organism	Position	Strand	Matrix score.	sequence	function
Skn-1_motif	Oryza sativa	5	-	5	GTCAT	cis-acting regulatory element required for endosperm expression
Skn-1_motif	Oryza sativa	345	+	5	GTCAT	cis-acting regulatory element required for

						endosperm expression
Skn-1_motif	Oryza sativa	40	+	5	GTCAT	cis-acting regulatory element required for endosperm expression
Skn-1_motif	Oryza sativa	1096	-	5	GTCAT	cis-acting regulatory element required for endosperm expression

+ TATC-box

Site Name	Organism	Position	Strand	Matrix score.	sequence	function
TATC-box	Oryza sativa	192	-	7	TATCCCA	cis-acting element involved in gibberellin-responsiveness

+ TC-rich repeats

Site Name	Organism	Position	Strand	Matrix score.	sequence	function
TC-rich repeats	Nicotiana tabacum	129	-	9	ATTTTCTTCA	cis-acting element involved in defense and stress responsiveness
TC-rich repeats	Nicotiana tabacum	1119	-	9	ATTTTCTTCA	cis-acting element involved in defense and stress responsiveness

+ TCA-element

Site Name	Organism	Position	Strand	Matrix score.	sequence	function
TCA-element	Nicotiana tabacum	695	+	9	CCATCTTTTT	cis-acting element involved in salicylic acid responsiveness

+ TGACG-motif

Site Name	Organism	Position	Strand	Matrix score.	sequence	function
TGACG-motif	Hordeum vulgare	163	-	5	TGACG	cis-acting regulatory element involved in the MeJA-responsiveness
TGACG-motif	Hordeum vulgare	1097	+	5	TGACG	cis-acting regulatory element involved in the MeJA-responsiveness
TGACG-motif	Hordeum vulgare	1020	-	5	TGACG	cis-acting regulatory element involved in the MeJA-responsiveness

SapurV1A.1059s0020 (*Salix purpurea*)

+ ARE

Site Name	Organism	Position	Strand	Matrix score.	sequence	function
ARE	Zea mays	122	+	6	TGGTTT	cis-acting regulatory element essential for the anaerobic induction
ARE	Zea mays	554	-	6	TGGTTT	cis-acting regulatory element essential for the anaerobic induction

+ CAT-box

Site Name	Organism	Position	Strand	Matrix score.	sequence	function
CAT-box	Arabidopsis thaliana	1453	-	6	GCCACT	cis-acting regulatory element related to meristem expression

+ GCN4_motif

Site Name	Organism	Position	Strand	Matrix score.	sequence	function
GCN4_motif	Oryza sativa	502	-	7	TGAGTCA	cis-regulatory element involved in endosperm expression

+ MBS

Site Name	Organism	Position	Strand	Matrix score.	sequence	function
MBS	Arabidopsis thaliana	1365	+	6	TAACTG	MYB binding site involved in drought-inducibility

+ O2-site

Site Name	Organism	Position	Strand	Matrix score.	sequence	function
O2-site	Zea mays	1345	-	9	GATGACATGA	cis-acting regulatory element involved in zein metabolism regulation

+ P-box

Site Name	Organism	Position	Strand	Matrix score.	sequence	function
P-box	Oryza sativa	721	-	7	CCTTTTG	gibberellin-responsive element

+ Skn-1_motif

Site Name	Organism	Position	Strand	Matrix score.	sequence	function
Skn-1_motif	Oryza sativa	401	+	5	GTCAT	cis-acting regulatory element required for endosperm expression
Skn-1_motif	Oryza sativa	1349	+	5	GTCAT	cis-acting regulatory element required for endosperm expression
Skn-1_motif	Oryza sativa	501	-	5	GTCAT	cis-acting regulatory element required for

						endosperm expression
Skn-1_motif	Oryza sativa	498	+	5	GTCAT	cis-acting regulatory element required for endosperm expression
Skn-1_motif	Oryza sativa	1109	-	5	GTCAT	cis-acting regulatory element required for endosperm expression

+ TC-rich repeats

Site Name	Organism	Position	Strand	Matrix score.	sequence	function
TC-rich repeats	Nicotiana tabacum	276	+	9	ATTCTCTAAC	cis-acting element involved in defense and stress responsiveness

+ TCA-element

Site Name	Organism	Position	Strand	Matrix score.	sequence	function
TCA-element	Brassica oleracea	320	-	9	GAGAAGAATA	cis-acting element involved in salicylic acid responsiveness

Potri.016G097600 (*Populus trichocarpa*)

+ ARE

Site Name	Organism	Position	Strand	Matrix score.	sequence	function
ARE	Zea mays	176	-	6	TGGTTT	cis-acting regulatory element essential for the anaerobic induction
ARE	Zea mays	1282	-	6	TGGTTT	cis-acting regulatory element essential for the anaerobic induction

+ Box-W1

Site Name	Organism	Position	Strand	Matrix score.	sequence	function
Box-W1	Petroselinum crispum	1198	+	6	TTGACC	fungal elicitor responsive element

+ GARE-motif

Site Name	Organism	Position	Strand	Matrix score.	sequence	function
GARE-motif	Brassica oleracea	1488	+	7	AAACAGA	gibberellin-responsive element

+ HSE

Site Name	Organism	Position	Strand	Matrix score.	sequence	function
HSE	Brassica oleracea	261	+	9	AAAAAATTTC	cis-acting element involved in heat stress responsiveness

+ MBS

Site Name	Organism	Position	Strand	Matrix score.	sequence	function
MBS	Arabidopsis thaliana	180	-	6	TAACTG	MYB binding site involved in drought-inducibility
MBS	Arabidopsis thaliana	810	+	6	TAACTG	MYB binding site involved in drought-inducibility
MBS	Arabidopsis thaliana	338	-	6	TAACTG	MYB binding site involved in drought-inducibility

+ Skn-1_motif

Site Name	Organism	Position	Strand	Matrix score.	sequence	function
Skn-1_motif	Oryza sativa	23	+	5	GTCAT	cis-acting regulatory element required for endosperm expression

+ TCA-element

Site Name	Organism	Position	Strand	Matrix score.	sequence	function

TCA-element	Brassica oleracea	931	+	9	GAGAAGAATA	cis-acting element involved in salicylic acid responsiveness
TCA-element	Brassica oleracea	1490	+	9	TCAGAAGAGG	cis-acting element involved in salicylic acid responsiveness

30078.t000042 (*Ricinus communis*)

+ Box-W1

Site Name	Organism	Position	Strand	Matrix score.	sequence	function
Box-W1	Petroselinum crispum	1004	+	6	TTGACC	fungal elicitor responsive element
Box-W1	Petroselinum crispum	1058	-	6	TTGACC	fungal elicitor responsive element

+ GARE-motif

Site Name	Organism	Position	Strand	Matrix score.	sequence	function
GARE-motif	Brassica oleracea	1483	+	7	AAACAGA	gibberellin-responsive element

+ HSE

Site Name	Organism	Position	Strand	Matrix score.	sequence	function
HSE	Brassica oleracea	563	-	9	AAAAAATTTC	cis-acting element involved in heat stress responsiveness
HSE	Brassica oleracea	889	+	9	AAAAAATTTC	cis-acting element involved in heat stress responsiveness

+ LTR

Site Name	Organism	Position	Strand	Matrix score.	sequence	function
LTR	Hordeum vulgare	810	-	6	CCGAAA	cis-acting element involved in low-temperature responsiveness
LTR	Hordeum vulgare	1305	+	6	CCGAAA	cis-acting element involved in low-temperature responsiveness

+ Skn-1_motif

Site Name	Organism	Position	Strand	Matrix score.	sequence	function
Skn-1_motif	Oryza sativa	198	-	5	GTCAT	cis-acting regulatory element required for endosperm expression
Skn-1_motif	Oryza sativa	1130	-	5	GTCAT	cis-acting regulatory element required for endosperm expression
Skn-1_motif	Oryza sativa	279	-	5	GTCAT	cis-acting regulatory element required for endosperm expression

+ TC-rich repeats

Site Name	Organism	Position	Strand	Matrix score.	sequence	function
TC-rich repeats	Nicotiana	245	-	9	ATTTTCTTCA	cis-acting element involved in defense and

	tabacum					stress responsiveness
TC-rich repeats	Nicotiana tabacum	687	-	9	ATTTTCTTCA	cis-acting element involved in defense and stress responsiveness

+ WUN-motif

Site Name	Organism	Position	Strand	Matrix score.	sequence	function
WUN-motif	Brassica oleracea	248	-	9	AAATTTCCT	wound-responsive element

Manes.09G061900 (*Manihot esculenta*)

+ ARE

Site Name	Organism	Position	Strand	Matrix score.	sequence	function
ARE	Zea mays	64	+	6	TGGTTT	cis-acting regulatory element essential for the anaerobic induction
ARE	Zea mays	282	+	6	TGGTTT	cis-acting regulatory element essential for the anaerobic induction

Box-W1

Site Name	Organism	Position	Strand	Matrix score.	sequence	function
Box-W1	Petroselinum crispum	261	-	6	TTGACC	fungal elicitor responsive element

+ P-box

Site Name	Organism	Position	Strand	Matrix score.	sequence	function
P-box	Oryza sativa	203	+	7	CCTTTTG	gibberellin-responsive element

Lus10022607.g (*Linum usitatissimum*)

+ ABRE

Site Name	Organism	Position	Strand	Matrix score.	sequence	function
ABRE	Hordeum vulgare	307	+	9	CCTACGTGGC	cis-acting element involved in the abscisic acid responsiveness
ABRE	Arabidopsis thaliana	309	+	6	TACGTG	cis-acting element involved in the abscisic acid responsiveness

+ ARE

Site Name	Organism	Position	Strand	Matrix score.	sequence	function
ARE	Zea mays	998	+	6	TGGTTT	cis-acting regulatory element essential for the anaerobic induction

+ Box-W1

Site Name	Organism	Position	Strand	Matrix score.	sequence	function
Box-W1	Petroselinum crispum	1056	+	6	TTGACC	fungal elicitor responsive element

+ C-repeat/DRE

Site Name	Organism	Position	Strand	Matrix score.	sequence	function
C-repeat/DRE	Arabidopsis thaliana	849	-	8	TGGCCGAC	regulatory element involved in cold- and dehydration-responsiveness

+ CAT-box

Site Name	Organism	Position	Strand	Matrix score.	sequence	function
CAT-box	Arabidopsis thaliana	1188	+	6	GCCACT	cis-acting regulatory element related to meristem expression

+ CGTCA-motif

Site Name	Organism	Position	Strand	Matrix score.	sequence	function
CGTCA-motif	Hordeum vulgare	1221	+	5	CGTCA	cis-acting regulatory element involved in the MeJA-responsiveness

+ ERE

Site Name	Organism	Position	Strand	Matrix score.	sequence	function
ERE	Dianthus caryophyllus	1114	-	8	ATTTCAAA	ethylene-responsive element

+ HSE

Site Name	Organism	Position	Strand	Matrix	sequence	function

				score.		
HSE	Brassica oleracea	226	-	9	AGAAAATTCG	cis-acting element involved in heat stress responsiveness
HSE	Brassica oleracea	1102	+	9	AAAAAATTTC	cis-acting element involved in heat stress responsiveness

+ LTR

Site Name	Organism	Position	Strand	Matrix score.	sequence	function
LTR	Hordeum vulgare	1157	-	6	CCGAAA	cis-acting element involved in low-temperature responsiveness

+ MBS

Site Name	Organism	Position	Strand	Matrix score.	sequence	function
MBS	Arabidopsis thaliana	375	-	6	TAACTG	MYB binding site involved in drought-inducibility

+ Skn-1_motif

Site Name	Organism	Position	Strand	Matrix score.	sequence	function
Skn-1_motif	Oryza sativa	811	+	5	GTCAT	cis-acting regulatory element required for endosperm expression
Skn-1_motif	Oryza sativa	1222	+	5	GTCAT	cis-acting regulatory element required for endosperm expression

+ TCA-element

Site Name	Organism	Position	Strand	Matrix score.	sequence	function
TCA-element	Brassica oleracea	676	+	9	GAGAAGAATA	cis-acting element involved in salicylic acid responsiveness
TCA-element	Brassica oleracea	1455	+	9	CAGAAAAGGA	cis-acting element involved in salicylic acid responsiveness

+ TGA-element

Site Name	Organism	Position	Strand	Matrix score.	sequence	function
TGA-element	Brassica oleracea	418	+	6	AACGAC	auxin-responsive element
TGA-element	Brassica oleracea	526	+	6	AACGAC	auxin-responsive element

+ TGACG-motif

Site Name	Organism	Position	Strand	Matrix score.	sequence	function

TGACG-motif	Hordeum vulgare	1221	-	5	TGACG	cis-acting regulatory element involved in the MeJA-responsiveness

Eucgr.K02436 (*Eucalyptus grandis*)

+ ARE

Site Name	Organism	Position	Strand	Matrix score.	sequence	function
ARE	Zea mays	257	+	6	TGGTTT	cis-acting regulatory element essential for the anaerobic induction
ARE	Zea mays	1193	+	6	TGGTTT	cis-acting regulatory element essential for the anaerobic induction

+ CGTCA-motif

Site Name	Organism	Position	Strand	Matrix score.	sequence	function
CGTCA-motif	Hordeum vulgare	176	+	5	CGTCA	cis-acting regulatory element involved in the MeJA-responsiveness
CGTCA-motif	Hordeum vulgare	637	+	5	CGTCA	cis-acting regulatory element involved in the MeJA-responsiveness

+ ERE

Site Name	Organism	Position	Strand	Matrix score.	sequence	function
ERE	Dianthus caryophyllus	280	-	8	ATTTCAAA	ethylene-responsive element

+ GCN4_motif

Site Name	Organism	Position	Strand	Matrix score.	sequence	function
GCN4_motif	Oryza sativa	601	-	7	CAAGCCA	cis-regulatory element involved in endosperm expression

+ HSE

Site Name	Organism	Position	Strand	Matrix score.	sequence	function
HSE	Brassica oleracea	221	-	9	AAAAAATTTC	cis-acting element involved in heat stress responsiveness

+ LTR

Site Name	Organism	Position	Strand	Matrix score.	sequence	function
LTR	Hordeum vulgare	1417	+	6	CCGAAA	cis-acting element involved in low-temperature responsiveness

+ Skn-1_motif

Site Name	Organism	Position	Strand	Matrix score.	sequence	function
Skn-1_motif	Oryza sativa	56	+	5	GTCAT	cis-acting regulatory element required for endosperm expression

Skn-1_motif	Oryza sativa	736	-	5	GTCAT	cis-acting regulatory element required for endosperm expression
Skn-1_motif	Oryza sativa	449	-	5	GTCAT	cis-acting regulatory element required for endosperm expression
Skn-1_motif	Oryza sativa	156	+	5	GTCAT	cis-acting regulatory element required for endosperm expression
Skn-1_motif	Oryza sativa	638	+	5	GTCAT	cis-acting regulatory element required for endosperm expression

+ TC-rich repeats

Site Name	Organism	Position	Strand	Matrix score.	sequence	function
TC-rich repeats	Nicotiana tabacum	7	-	9	ATTTTCTTCA	cis-acting element involved in defense and stress responsiveness
TC-rich repeats	Nicotiana tabacum	715	-	9	ATTTTCTTCA	cis-acting element involved in defense and stress responsiveness

+ TGACG-motif

Site Name	Organism	Position	Strand	Matrix score.	sequence	function
TGACG-motif	Hordeum vulgare	176	-	5	TGACG	cis-acting regulatory element involved in the MeJA-responsiveness
TGACG-motif	Hordeum vulgare	637	-	5	TGACG	cis-acting regulatory element involved in the MeJA-responsiveness

AHYPO_000598 (*Amaranthus hypochondriacus*)

+ ABRE

Site Name	Organism	Position	Strand	Matrix score.	sequence	function
ABRE	Arabidopsis thaliana	1405	-	6	CACGTG	cis-acting element involved in the abscisic acid responsiveness

+ Box-W1

Site Name	Organism	Position	Strand	Matrix score.	sequence	function
Box-W1	Petroselinum crispum	871	-	6	TTGACC	fungal elicitor responsive element
Box-W1	Petroselinum crispum	1278	+	6	TTGACC	fungal elicitor responsive element
Box-W1	Petroselinum crispum	1125	+	6	TTGACC	fungal elicitor responsive element

+ CE3

Site Name	Organism	Position	Strand	Matrix score.	sequence	function
CE3	Oryza sativa	988	-	9	GACGCGTGTC	cis-acting element involved in ABA and VP1 responsiveness

+ CGTCA-motif

Site Name	Organism	Position	Strand	Matrix score.	sequence	function
CGTCA-motif	Hordeum vulgare	1484	-	5	CGTCA	cis-acting regulatory element involved in the MeJA-responsiveness

+ ERE

Site Name	Organism	Position	Strand	Matrix score.	sequence	function
ERE	Dianthus caryophyllus	1271	-	8	ATTTCAAA	ethylene-responsive element

+ GCN4_motif

Site Name	Organism	Position	Strand	Matrix score.	sequence	function
GCN4_motif	Oryza sativa	1240	-	7	TGAGTCA	cis-regulatory element involved in endosperm expression

+ HSE

Site Name	Organism	Position	Strand	Matrix score.	sequence	function
HSE	Brassica oleracea	1148	+	9	AAAAAATTTC	cis-acting element involved in heat stress responsiveness

+ Skn-1_motif

Site Name	Organism	Position	Strand	Matrix score.	sequence	function
Skn-1_motif	Oryza sativa	118	-	5	GTCAT	cis-acting regulatory element required for endosperm expression
Skn-1_motif	Oryza sativa	1322	+	5	GTCAT	cis-acting regulatory element required for endosperm expression
Skn-1_motif	Oryza sativa	1137	+	5	GTCAT	cis-acting regulatory element required for endosperm expression
Skn-1_motif	Oryza sativa	673	-	5	GTCAT	cis-acting regulatory element required for endosperm expression
Skn-1_motif	Oryza sativa	1239	-	5	GTCAT	cis-acting regulatory element required for endosperm expression

+ TC-rich repeats

Site Name	Organism	Position	Strand	Matrix score.	sequence	function
TC-rich repeats	Nicotiana tabacum	326	+	10	ATTTTCTTCA	cis-acting element involved in defense and stress responsiveness
TC-rich repeats	Nicotiana tabacum	384	-	9	ATTTTCTTCA	cis-acting element involved in defense and stress responsiveness

+ TCA-element

Site Name	Organism	Position	Strand	Matrix score.	sequence	function
TCA-element	Brassica oleracea	166	+	9	CAGAAAAGGA	cis-acting element involved in salicylic acid responsiveness

+ TGACG-motif

Site Name	Organism	Position	Strand	Matrix score.	sequence	function
TGACG-motif	Hordeum vulgare	1484	+	5	TGACG	cis-acting regulatory element involved in the MeJA-responsiveness

AHYPO_001149 (*Amaranthus hypochondriacus*)

+ ARE

Site Name	Organism	Position	Strand	Matrix score.	sequence	function
ARE	Zea mays	1165	-	6	TGGTTT	cis-acting regulatory element essential for the anaerobic induction
ARE	Zea mays	1273	-	6	TGGTTT	cis-acting regulatory element essential for the anaerobic induction
ARE	Zea mays	1193	-	6	TGGTTT	cis-acting regulatory element essential for the anaerobic induction

+ CGTCA-motif

Site Name	Organism	Position	Strand	Matrix score.	sequence	function
CGTCA-motif	Hordeum vulgare	426	+	5	CGTCA	cis-acting regulatory element involved in the MeJA-responsiveness

+ GARE-motif

Site Name	Organism	Position	Strand	Matrix score.	sequence	function
GARE-motif	Brassica oleracea	1254	+	7	AAACAGA	gibberellin-responsive element

+ GCN4_motif

Site Name	Organism	Position	Strand	Matrix score.	sequence	function
GCN4_motif	Oryza sativa	688	-	7	CAAGCCA	cis-regulatory element involved in endosperm expression
GCN4_motif	Oryza sativa	747	+	7	TGAGTCA	cis-regulatory element involved in endosperm expression

+ HSE

Site Name	Organism	Position	Strand	Matrix score.	sequence	function
HSE	Brassica oleracea	36	+	9	AAAAAATTTC	cis-acting element involved in heat stress responsiveness
HSE	Brassica oleracea	1228	-	9	AAAAAATTTC	cis-acting element involved in heat stress responsiveness

+ LTR

Site Name	Organism	Position	Strand	Matrix score.	sequence	function
LTR	Hordeum vulgare	1208	+	6	CCGAAA	cis-acting element involved in low-temperature responsiveness

+ MBS

Site Name	Organism	Position	Strand	Matrix score.	sequence	function
MBS	Arabidopsis thaliana	1073	-	6	CAACTG	MYB binding site involved in drought-inducibility

+ P-box

Site Name	Organism	Position	Strand	Matrix score.	sequence	function
P-box	Oryza sativa	1027	-	7	CCTTTTG	gibberellin-responsive element

+ Skn-1_motif

Site Name	Organism	Position	Strand	Matrix score.	sequence	function
Skn-1_motif	Oryza sativa	270	-	5	GTCAT	cis-acting regulatory element required for endosperm expression
Skn-1_motif	Oryza sativa	750	+	5	GTCAT	cis-acting regulatory element required for endosperm expression

+ TC-rich repeats

Site Name	Organism	Position	Strand	Matrix score.	sequence	function
TC-rich repeats	Nicotiana tabacum	591	-	9	ATTCTCTAAC	cis-acting element involved in defense and stress responsiveness
TC-rich repeats	Nicotiana tabacum	1130	+	9	ATTTTCTTCA	cis-acting element involved in defense and stress responsiveness

+ TCA-element

Site Name	Organism	Position	Strand	Matrix score.	sequence	function
TCA-element	Nicotiana tabacum	435	+	9	CCATCTTTTT	cis-acting element involved in salicylic acid responsiveness

+ TGACG-motif

Site Name	Organism	Position	Strand	Matrix score.	sequence	function
TGACG-motif	Hordeum vulgare	426	-	5	TGACG	cis-acting regulatory element involved in the MeJA-responsiveness

AUR62026953 (*Chenopodium quinoa*)

+ Box-W1

Site Name	Organism	Position	Strand	Matrix score.	sequence	function
Box-W1	Petroselinum crispum	914	-	6	TTGACC	fungal elicitor responsive element

+ CGTCA-motif

Site Name	Organism	Position	Strand	Matrix score.	sequence	function
CGTCA-motif	Hordeum vulgare	270	-	5	CGTCA	cis-acting regulatory element involved in the MeJA-responsiveness

+ HSE

Site Name	Organism	Position	Strand	Matrix score.	sequence	function
HSE	Brassica oleracea	1222	+	9	AAAAAATTTC	cis-acting element involved in heat stress responsiveness

+ Skn-1_motif

Site Name	Organism	Position	Strand	Matrix score.	sequence	function
Skn-1_motif	Oryza sativa	631	+	5	GTCAT	cis-acting regulatory element required for endosperm expression
Skn-1_motif	Oryza sativa	901	-	5	GTCAT	cis-acting regulatory element required for endosperm expression

+ TC-rich repeats

Site Name	Organism	Position	Strand	Matrix score.	sequence	function
TC-rich repeats	Nicotiana tabacum	409	-	9	ATTTTCTTCA	cis-acting element involved in defense and stress responsiveness
TC-rich repeats	Nicotiana tabacum	1226	+	10	ATTTTCTTCA	cis-acting element involved in defense and stress responsiveness
TC-rich repeats	Nicotiana tabacum	434	-	9	ATTTTCTTCA	cis-acting element involved in defense and stress responsiveness

+ TCA-element

Site Name	Organism	Position	Strand	Matrix score.	sequence	function
TCA-element	Brassica oleracea	76	-	9	TCAGAAGAGG	cis-acting element involved in salicylic acid responsiveness
TCA-element	Nicotiana tabacum	959	-	9	CCATCTTTTT	cis-acting element involved in salicylic acid responsiveness

+ TGACG-motif

Site Name	Organism	Position	Strand	Matrix score.	sequence	function
TGACG-motif	Hordeum vulgare	270	+	5	TGACG	cis-acting regulatory element involved in the MeJA-responsiveness

+ WUN-motif

Site Name	Organism	Position	Strand	Matrix score.	sequence	function
WUN-motif	Brassica oleracea	65	+	9	TCATTACGAA	wound-responsive element

Zm00008a035638 (*Zea mays*)

+ ABRE

Site Name	Organism	Position	Strand	Matrix score.	sequence	function
ABRE	Arabidopsis thaliana	91	+	7	TACGGTC	cis-acting element involved in the abscisic acid responsiveness
ABRE	Oryza sativa	119	+	9	GCCGCGTGGC	cis-acting element involved in the abscisic acid responsiveness

+ ARE

Site Name	Organism	Position	Strand	Matrix score.	sequence	function
ARE	Zea mays	566	-	6	TGGTTT	cis-acting regulatory element essential for the anaerobic induction
ARE	Zea mays	1108	-	6	TGGTTT	cis-acting regulatory element essential for the anaerobic induction

+ Box-W1

Site Name	Organism	Position	Strand	Matrix score.	sequence	function
Box-W1	Petroselinum crispum	585	+	6	TTGACC	fungal elicitor responsive element

+ CGTCA-motif

Site Name	Organism	Position	Strand	Matrix score.	sequence	function
CGTCA-motif	Hordeum vulgare	524	-	5	CGTCA	cis-acting regulatory element involved in the MeJA-responsiveness

+ GC-motif

Site Name	Organism	Position	Strand	Matrix score.	sequence	function
GC-motif	Zea mays	51	+	8	CCACGGGG	enhancer-like element involved in anoxic specific inducibility
GC-motif	Zea mays	884	-	7	GCCCCGG	enhancer-like element involved in anoxic specific inducibility

+ MBS

Site Name	Organism	Position	Strand	Matrix score.	sequence	function
MBS	Arabidopsis thaliana	1024	-	6	CAACTG	MYB binding site involved in drought-inducibility

+ TC-rich repeats

Site Name	Organism	Position	Strand	Matrix score.	sequence	function

TC-rich repeats	Nicotiana tabacum	1346	+	9	ATTTTCTCCA	cis-acting element involved in defense and stress responsiveness

+ TCA-element

Site Name	Organism	Position	Strand	Matrix score.	sequence	function
TCA-element	Brassica oleracea	206	-	9	GAGAAGAATA	cis-acting element involved in salicylic acid responsiveness

+ TGA-element

Site Name	Organism	Position	Strand	Matrix score.	sequence	function
TGA-element	Brassica oleracea	103	-	6	AACGAC	auxin-responsive element
TGA-element	Brassica oleracea	1069	+	6	AACGAC	auxin-responsive element
TGA-element	Brassica oleracea	175	+	6	AACGAC	auxin-responsive element

+ TGACG-motif

Site Name	Organism	Position	Strand	Matrix score.	sequence	function
TGACG-motif	Hordeum vulgare	524	+	5	TGACG	cis-acting regulatory element involved in the MeJA-responsiveness

+ motif IIb

Site Name	Organism	Position	Strand	Matrix score.	sequence	function
motif IIb	Oryza sativa	113	-	9	CCGCCGCGCT	abscisic acid responsive element

GRMZM2G307906 (*Zea mays*)

+ ABRE

Site Name	Organism	Position	Strand	Matrix score.	sequence	function
ABRE	Hordeum vulgare	85	+	9	CGTACGTGCA	cis-acting element involved in the abscisic acid responsiveness
ABRE	Arabidopsis thaliana	87	+	6	TACGTG	cis-acting element involved in the abscisic acid responsiveness

+ ARE

Site Name	Organism	Position	Strand	Matrix score.	sequence	function
ARE	Zea mays	722	-	6	TGGTTT	cis-acting regulatory element essential for the anaerobic induction
ARE	Zea mays	1206	-	6	TGGTTT	cis-acting regulatory element essential for the anaerobic induction

+ CGTCA-motif

Site Name	Organism	Position	Strand	Matrix score.	sequence	function
CGTCA-motif	Hordeum vulgare	493	+	5	CGTCA	cis-acting regulatory element involved in the MeJA-responsiveness
CGTCA-motif	Hordeum vulgare	1339	+	5	CGTCA	cis-acting regulatory element involved in the MeJA-responsiveness
CGTCA-motif	Hordeum vulgare	824	-	5	CGTCA	cis-acting regulatory element involved in the MeJA-responsiveness

+ HSE

Site Name	Organism	Position	Strand	Matrix score.	sequence	function
HSE	Brassica oleracea	300	+	9	AAAAAATTTC	cis-acting element involved in heat stress responsiveness

+ LTR

Site Name	Organism	Position	Strand	Matrix score.	sequence	function
LTR	Hordeum vulgare	407	-	6	CCGAAA	cis-acting element involved in low-temperature responsiveness
LTR	Hordeum vulgare	1187	+	6	CCGAAA	cis-acting element involved in low-temperature responsiveness
LTR	Hordeum vulgare	430	-	6	CCGAAA	cis-acting element involved in low-temperature responsiveness

+ Skn-1_motif

Site Name	Organism	Position	Strand	Matrix	sequence	function

				score.		
Skn-1_motif	Oryza sativa	823	-	5	GTCAT	cis-acting regulatory element required for endosperm expression
Skn-1_motif	Oryza sativa	1361	+	5	GTCAT	cis-acting regulatory element required for endosperm expression
Skn-1_motif	Oryza sativa	935	+	5	GTCAT	cis-acting regulatory element required for endosperm expression
Skn-1_motif	Oryza sativa	924	-	5	GTCAT	cis-acting regulatory element required for endosperm expression
Skn-1_motif	Oryza sativa	1273	+	5	GTCAT	cis-acting regulatory element required for endosperm expression

+ TC-rich repeats

Site Name	Organism	Position	Strand	Matrix score.	sequence	function
TC-rich repeats	Nicotiana tabacum	248	+	9	GTTTTCTTAC	cis-acting element involved in defense and stress responsiveness
TC-rich repeats	Nicotiana tabacum	903	+	9	ATTTTCTTCA	cis-acting element involved in defense and stress responsiveness
TC-rich repeats	Nicotiana tabacum	879	+	9	ATTTTCTTCA	cis-acting element involved in defense and stress responsiveness

+ TCA-element

Site Name	Organism	Position	Strand	Matrix score.	sequence	function
TCA-element	Nicotiana tabacum	219	-	9	CCATCTTTTT	cis-acting element involved in salicylic acid responsiveness
TCA-element	Brassica oleracea	1426	-	9	TCAGAAGAGG	cis-acting element involved in salicylic acid responsiveness
TCA-element	Brassica oleracea	880	-	9	GAGAAGAATA	cis-acting element involved in salicylic acid responsiveness

+ TGACG-motif

Site Name	Organism	Position	Strand	Matrix score.	sequence	function
TGACG-motif	Hordeum vulgare	493	-	5	TGACG	cis-acting regulatory element involved in the MeJA-responsiveness
TGACG-motif	Hordeum vulgare	1339	-	5	TGACG	cis-acting regulatory element involved in the MeJA-responsiveness
TGACG-motif	Hordeum vulgare	824	+	5	TGACG	cis-acting regulatory element involved in the MeJA-responsiveness

Zm00008a001017 (*Zea mays*)

+ ABRE

Site Name	Organism	Position	Strand	Matrix score.	sequence	function
ABRE	Oryza sativa	1453	+	9	GCCGCGTGGC	cis-acting element involved in the abscisic acid responsiveness

+ Box-W1

Site Name	Organism	Position	Strand	Matrix score.	sequence	function
Box-W1	Petroselinum crispum	877	+	6	TTGACC	fungal elicitor responsive element
Box-W1	Petroselinum crispum	1432	+	6	TTGACC	fungal elicitor responsive element

+ Skn-1_motif

Site Name	Organism	Position	Strand	Matrix score.	sequence	function
Skn-1_motif	Oryza sativa	470	-	5	GTCAT	cis-acting regulatory element required for endosperm expression

+ TC-rich repeats

Site Name	Organism	Position	Strand	Matrix score.	sequence	function
TC-rich repeats	Nicotiana tabacum	588	+	9	ATTTTCTTCA	cis-acting element involved in defense and stress responsiveness

GRMZM2G100620 (*Zea mays*)

+ ABRE

Site Name	Organism	Position	Strand	Matrix score.	sequence	function
ABRE	Oryza sativa	198	+	9	AGTACGTGGC	cis-acting element involved in the abscisic acid responsiveness
ABRE	Hordeum vulgare	731	-	9	CCTACGTGGC	cis-acting element involved in the abscisic acid responsiveness
ABRE	Arabidopsis thaliana	200	+	6	TACGTG	cis-acting element involved in the abscisic acid responsiveness
ABRE	Arabidopsis thaliana	733	-	6	TACGTG	cis-acting element involved in the abscisic acid responsiveness

+ ARE

Site Name	Organism	Position	Strand	Matrix score.	sequence	function
ARE	Zea mays	381	+	6	TGGTTT	cis-acting regulatory element essential for the anaerobic induction
ARE	Zea mays	762	+	6	TGGTTT	cis-acting regulatory element essential for the anaerobic induction
ARE	Zea mays	443	+	6	TGGTTT	cis-acting regulatory element essential for the anaerobic induction

+ Box-W1

Site Name	Organism	Position	Strand	Matrix score.	sequence	function
Box-W1	Petroselinum crispum	496	+	6	TTGACC	fungal elicitor responsive element

+ CAT-box

Site Name	Organism	Position	Strand	Matrix score.	sequence	function
CAT-box	Arabidopsis thaliana	839	+	6	GCCACT	cis-acting regulatory element related to meristem expression

+ CGTCA-motif

Site Name	Organism	Position	Strand	Matrix score.	sequence	function
CGTCA-motif	Hordeum vulgare	467	-	5	CGTCA	cis-acting regulatory element involved in the MeJA-responsiveness
CGTCA-motif	Hordeum vulgare	1086	+	5	CGTCA	cis-acting regulatory element involved in the MeJA-responsiveness

+ GARE-motif

Site Name	Organism	Position	Strand	Matrix	sequence	function

				score.		
GARE-motif	Brassica oleracea	881	-	7	AAACAGA	gibberellin-responsive element

+ HSE

Site Name	Organism	Position	Strand	Matrix score.	sequence	function
HSE	Brassica oleracea	176	-	9	AAAAAATTTC	cis-acting element involved in heat stress responsiveness

+ MBS

Site Name	Organism	Position	Strand	Matrix score.	sequence	function
MBS	Arabidopsis thaliana	213	-	6	CAACTG	MYB binding site involved in drought-inducibility

+ Skn-1_motif

Site Name	Organism	Position	Strand	Matrix score.	sequence	function
Skn-1_motif	Oryza sativa	174	-	5	GTCAT	cis-acting regulatory element required for endosperm expression
Skn-1_motif	Oryza sativa	1271	-	5	GTCAT	cis-acting regulatory element required for endosperm expression
Skn-1_motif	Oryza sativa	947	+	5	GTCAT	cis-acting regulatory element required for endosperm expression
Skn-1_motif	Oryza sativa	547	+	5	GTCAT	cis-acting regulatory element required for endosperm expression
Skn-1_motif	Oryza sativa	1235	-	5	GTCAT	cis-acting regulatory element required for endosperm expression

+ TC-rich repeats

Site Name	Organism	Position	Strand	Matrix score.	sequence	function
TC-rich repeats	Nicotiana tabacum	959	-	9	GTTTTCTTAC	cis-acting element involved in defense and stress responsiveness
TC-rich repeats	Nicotiana tabacum	1389	+	9	ATTTTCTTCA	cis-acting element involved in defense and stress responsiveness

+ TGA-element

Site Name	Organism	Position	Strand	Matrix score.	sequence	function
TGA-element	Brassica oleracea	991	-	6	AACGAC	auxin-responsive element

+ TGACG-motif

Site Name	Organism	Position	Strand	Matrix	sequence	function

				score.		
TGACG-motif	Hordeum vulgare	467	+	5	TGACG	cis-acting regulatory element involved in the MeJA-responsiveness
TGACG-motif	Hordeum vulgare	1086	-	5	TGACG	cis-acting regulatory element involved in the MeJA-responsiveness

+ WUN-motif

Site Name	Organism	Position	Strand	Matrix score.	sequence	function
WUN-motif	Brassica oleracea	430	+	9	TCATTACGAA	wound-responsive element

Sobic.001G428400 (*Sorghum bicolor*)

+ ABRE

Site Name	Organism	Position	Strand	Matrix score.	sequence	function
ABRE	Oryza sativa	446	-	9	GCCGCGTGGC	cis-acting element involved in the abscisic acid responsiveness

+ Box-W1

Site Name	Organism	Position	Strand	Matrix score.	sequence	function
Box-W1	Petroselinum crispum	630	+	6	TTGACC	fungal elicitor responsive element

+ CCGTCC-box

Site Name	Organism	Position	Strand	Matrix score.	sequence	function
CCGTCC-box	Arabidopsis thaliana	110	+	6	CCGTCC	cis-acting regulatory element related to meristem specific activation

+ CGTCA-motif

Site Name	Organism	Position	Strand	Matrix score.	sequence	function
CGTCA-motif	Hordeum vulgare	489	-	5	CGTCA	cis-acting regulatory element involved in the MeJA-responsiveness
CGTCA-motif	Hordeum vulgare	894	+	5	CGTCA	cis-acting regulatory element involved in the MeJA-responsiveness

+ GC-motif

Site Name	Organism	Position	Strand	Matrix score.	sequence	function
GC-motif	Zea mays	586	-	7	GCCCCGG	enhancer-like element involved in anoxic specific inducibility

+ HSE

Site Name	Organism	Position	Strand	Matrix score.	sequence	function
HSE	Brassica oleracea	1211	-	9	AAAAAATTTC	cis-acting element involved in heat stress responsiveness

+ Skn-1_motif

Site Name	Organism	Position	Strand	Matrix score.	sequence	function
Skn-1_motif	Oryza sativa	1166	+	5	GTCAT	cis-acting regulatory element required for endosperm expression
Skn-1_motif	Oryza sativa	1279	-	5	GTCAT	cis-acting regulatory element required for endosperm expression

+ TATC-box

Site Name	Organism	Position	Strand	Matrix score.	sequence	function
TATC-box	Oryza sativa	1016	-	7	TATCCCA	cis-acting element involved in gibberellin-responsiveness

+ TC-rich repeats

Site Name	Organism	Position	Strand	Matrix score.	sequence	function
TC-rich repeats	Nicotiana tabacum	1006	-	9	ATTCTCTAAC	cis-acting element involved in defense and stress responsiveness
TC-rich repeats	Nicotiana tabacum	1258	+	9	ATTTTCTCCA	cis-acting element involved in defense and stress responsiveness
TC-rich repeats	Nicotiana tabacum	1077	-	9	ATTCTCTAAC	cis-acting element involved in defense and stress responsiveness
TC-rich repeats	Nicotiana tabacum	1271	-	9	ATTTTCTCCA	cis-acting element involved in defense and stress responsiveness

+ TCA-element

Site Name	Organism	Position	Strand	Matrix score.	sequence	function
TCA-element	Brassica oleracea	1322	+	10	CAGAAAAGGA	cis-acting element involved in salicylic acid responsiveness

+ TGACG-motif

Site Name	Organism	Position	Strand	Matrix score.	sequence	function
TGACG-motif	Hordeum vulgare	489	+	5	TGACG	cis-acting regulatory element involved in the MeJA-responsiveness
TGACG-motif	Hordeum vulgare	894	-	5	TGACG	cis-acting regulatory element involved in the MeJA-responsiveness

+ as-2-box

Site Name	Organism	Position	Strand	Matrix score.	sequence	function
as-2-box	Nicotiana tabacum	295	+	9	GATAatGATG	involved in shoot-specific expression and light responsiveness

Pavir.9KG504400 (*Panicum virgatum*)

+ ABRE

Site Name	Organism	Position	Strand	Matrix score.	sequence	function
ABRE	Oryza sativa	695	-	9	GCCGCGTGGC	cis-acting element involved in the abscisic acid responsiveness
ABRE	Arabidopsis thaliana	1251	-	6	CACGTG	cis-acting element involved in the abscisic acid responsiveness
ABRE	Arabidopsis thaliana	790	-	6	CACGTG	cis-acting element involved in the abscisic acid responsiveness
ABRE	Hordeum vulgare	1262	+	9	CGTACGTGCA	cis-acting element involved in the abscisic acid responsiveness
ABRE	Arabidopsis thaliana	773	-	6	CACGTG	cis-acting element involved in the abscisic acid responsiveness
ABRE	Oryza sativa	1252	-	10	GACACGTACGT	cis-acting element involved in the abscisic acid responsiveness
ABRE	Hordeum vulgare	1249	-	9	CGCACGTGTC	cis-acting element involved in the abscisic acid responsiveness
ABRE	Arabidopsis thaliana	1264	-	6	CACGTG	cis-acting element involved in the abscisic acid responsiveness

+ ARE

Site Name	Organism	Position	Strand	Matrix score.	sequence	function
ARE	Zea mays	1329	+	6	TGGTTT	cis-acting regulatory element essential for the anaerobic induction

+ Box-W1

Site Name	Organism	Position	Strand	Matrix score.	sequence	function
Box-W1	Petroselinum crispum	110	+	6	TTGACC	fungal elicitor responsive element

+ CGTCA-motif

Site Name	Organism	Position	Strand	Matrix score.	sequence	function
CGTCA-motif	Hordeum vulgare	167	+	5	CGTCA	cis-acting regulatory element involved in the MeJA-responsiveness
CGTCA-motif	Hordeum vulgare	1031	+	5	CGTCA	cis-acting regulatory element involved in the MeJA-responsiveness
CGTCA-motif	Hordeum vulgare	906	-	5	CGTCA	cis-acting regulatory element involved in the MeJA-responsiveness

+ LTR

Site Name	Organism	Position	Strand	Matrix score.	sequence	function
LTR	Hordeum vulgare	413	-	6	CCGAAA	cis-acting element involved in low-temperature responsiveness

+ MBS

Site Name	Organism	Position	Strand	Matrix score.	sequence	function
MBS	Arabidopsis thaliana	796	+	6	CAACTG	MYB binding site involved in drought-inducibility

+ Skn-1_motif

Site Name	Organism	Position	Strand	Matrix score.	sequence	function
Skn-1_motif	Oryza sativa	905	-	5	GTCAT	cis-acting regulatory element required for endosperm expression
Skn-1_motif	Oryza sativa	1032	+	5	GTCAT	cis-acting regulatory element required for endosperm expression

+ TC-rich repeats

Site Name	Organism	Position	Strand	Matrix score.	sequence	function
TC-rich repeats	Nicotiana tabacum	348	+	9	ATTCTCTAAC	cis-acting element involved in defense and stress responsiveness

+ TCA-element

Site Name	Organism	Position	Strand	Matrix score.	sequence	function
TCA-element	Brassica oleracea	473	+	9	CAGAAAAGGA	cis-acting element involved in salicylic acid responsiveness
TCA-element	Nicotiana tabacum	1123	+	9	CCATCTTTTT	cis-acting element involved in salicylic acid responsiveness

+ TGA-element

Site Name	Organism	Position	Strand	Matrix score.	sequence	function
TGA-element	Brassica oleracea	1103	+	6	AACGAC	auxin-responsive element

+ TGACG-motif

Site Name	Organism	Position	Strand	Matrix score.	sequence	function
TGACG-motif	Hordeum vulgare	167	-	5	TGACG	cis-acting regulatory element involved in the MeJA-responsiveness
TGACG-motif	Hordeum vulgare	1031	-	5	TGACG	cis-acting regulatory element involved in the MeJA-responsiveness

TGACG-motif	Hordeum vulgare	906	+	5	TGACG	cis-acting regulatory element involved in the MeJA-responsiveness

Pavir.9NG665400 (*Panicum virgatum*)

+ ABRE

Site Name	Organism	Position	Strand	Matrix score.	sequence	function
ABRE	Oryza sativa	296	+	9	GCCGCGTGGC	cis-acting element involved in the abscisic acid responsiveness
ABRE	Oryza sativa	1032	-	9	GCCGCGTGGC	cis-acting element involved in the abscisic acid responsiveness
ABRE	Oryza sativa	960	-	9	GCCGCGTGGC	cis-acting element involved in the abscisic acid responsiveness

+ Box-W1

Site Name	Organism	Position	Strand	Matrix score.	sequence	function
Box-W1	Petroselinum crispum	631	+	6	TTGACC	fungal elicitor responsive element

+ CAT-box

Site Name	Organism	Position	Strand	Matrix score.	sequence	function
CAT-box	Arabidopsis thaliana	479	-	6	GCCACT	cis-acting regulatory element related to meristem expression

+ CCGTCC-box

Site Name	Organism	Position	Strand	Matrix score.	sequence	function
CCGTCC-box	Arabidopsis thaliana	979	-	6	CCGTCC	cis-acting regulatory element related to meristem specific activation

+ CGTCA-motif

Site Name	Organism	Position	Strand	Matrix score.	sequence	function
CGTCA-motif	Hordeum vulgare	1003	-	5	CGTCA	cis-acting regulatory element involved in the MeJA-responsiveness

+ GARE-motif

Site Name	Organism	Position	Strand	Matrix score.	sequence	function
GARE-motif	Brassica oleracea	677	+	7	AAACAGA	gibberellin-responsive element

+ GC-motif

Site Name	Organism	Position	Strand	Matrix score.	sequence	function
GC-motif	Zea mays	1100	-	7	GCCCCGG	enhancer-like element involved in anoxic specific inducibility

+ GCN4_motif

Site Name	Organism	Position	Strand	Matrix score.	sequence	function
GCN4_motif	Oryza sativa	101	-	7	TGTGTCA	cis-regulatory element involved in endosperm expression

+ MBS

Site Name	Organism	Position	Strand	Matrix score.	sequence	function
MBS	Arabidopsis thaliana	628	-	6	CAACTG	MYB binding site involved in drought-inducibility

+ Skn-1_motif

Site Name	Organism	Position	Strand	Matrix score.	sequence	function
Skn-1_motif	Oryza sativa	1	+	5	GTCAT	cis-acting regulatory element required for endosperm expression
Skn-1_motif	Oryza sativa	1113	+	5	GTCAT	cis-acting regulatory element required for endosperm expression
Skn-1_motif	Oryza sativa	68	-	5	GTCAT	cis-acting regulatory element required for endosperm expression

+ TGA-element

Site Name	Organism	Position	Strand	Matrix score.	sequence	function
TGA-element	Brassica oleracea	168	+	6	AACGAC	auxin-responsive element
TGA-element	Brassica oleracea	352	+	6	AACGAC	auxin-responsive element

+ TGACG-motif

Site Name	Organism	Position	Strand	Matrix score.	sequence	function
TGACG-motif	Hordeum vulgare	1003	+	5	TGACG	cis-acting regulatory element involved in the MeJA-responsiveness

Pavir.9KG512300 (*Panicum virgatum*)

+ ABRE

Site Name	Organism	Position	Strand	Matrix score.	sequence	function
ABRE	Hordeum vulgare	279	-	9	GCCACGTACA	cis-acting element involved in the abscisic acid responsiveness
ABRE	Oryza sativa	1008	-	10	GCCGCGTGGC	cis-acting element involved in the abscisic acid responsiveness
ABRE	Arabidopsis thaliana	282	+	7	ACGTGGC	cis-acting element involved in the abscisic acid responsiveness
ABRE	Oryza sativa	1080	-	9	GCCGCGTGGC	cis-acting element involved in the abscisic acid responsiveness
ABRE	Arabidopsis thaliana	281	+	6	TACGTG	cis-acting element involved in the abscisic acid responsiveness
ABRE	Oryza sativa	327	+	9	GCCGCGTGGC	cis-acting element involved in the abscisic acid responsiveness

+ ARE

Site Name	Organism	Position	Strand	Matrix score.	sequence	function
ARE	Zea mays	461	-	6	TGGTTT	cis-acting regulatory element essential for the anaerobic induction

+ C-repeat/DRE

Site Name	Organism	Position	Strand	Matrix score.	sequence	function
C-repeat/DRE	Arabidopsis thaliana	285	+	8	TGGCCGAC	regulatory element involved in cold- and dehydration-responsiveness

+ CAT-box

Site Name	Organism	Position	Strand	Matrix score.	sequence	function
CAT-box	Arabidopsis thaliana	530	-	6	GCCACT	cis-acting regulatory element related to meristem expression

+ CCGTCC-box

Site Name	Organism	Position	Strand	Matrix score.	sequence	function
CCGTCC-box	Arabidopsis thaliana	886	-	6	CCGTCC	cis-acting regulatory element related to meristem specific activation
CCGTCC-box	Arabidopsis thaliana	1027	-	6	CCGTCC	cis-acting regulatory element related to meristem specific activation

+ CGTCA-motif

Site Name	Organism	Position	Strand	Matrix	sequence	function

				score.		
CGTCA-motif	Hordeum vulgare	67	+	5	CGTCA	cis-acting regulatory element involved in the MeJA-responsiveness
CGTCA-motif	Hordeum vulgare	1051	-	5	CGTCA	cis-acting regulatory element involved in the MeJA-responsiveness

+ GC-motif

Site Name	Organism	Position	Strand	Matrix score.	sequence	function
GC-motif	Zea mays	1148	-	7	GCCCCGG	enhancer-like element involved in anoxic specific inducibility

+ GCN4_motif

Site Name	Organism	Position	Strand	Matrix score.	sequence	function
GCN4_motif	Oryza sativa	132	-	7	TGTGTCA	cis-regulatory element involved in endosperm expression

+ LTR

Site Name	Organism	Position	Strand	Matrix score.	sequence	function
LTR	Hordeum vulgare	1462	+	6	CCGAAA	cis-acting element involved in low-temperature responsiveness

+ MBS

Site Name	Organism	Position	Strand	Matrix score.	sequence	function
MBS	Arabidopsis thaliana	687	+	6	CAACTG	MYB binding site involved in drought-inducibility

+ Skn-1_motif

Site Name	Organism	Position	Strand	Matrix score.	sequence	function
Skn-1_motif	Oryza sativa	68	+	5	GTCAT	cis-acting regulatory element required for endosperm expression
Skn-1_motif	Oryza sativa	1161	+	5	GTCAT	cis-acting regulatory element required for endosperm expression
Skn-1_motif	Oryza sativa	610	-	5	GTCAT	cis-acting regulatory element required for endosperm expression
Skn-1_motif	Oryza sativa	1399	+	5	GTCAT	cis-acting regulatory element required for endosperm expression

+ TCA-element

Site Name	Organism	Position	Strand	Matrix score.	sequence	function
TCA-element	Brassica	509	-	9	CAGAAAAGGA	cis-acting element involved in salicylic

	oleracea					acid responsiveness

+ TGA-element

Site Name	Organism	Position	Strand	Matrix score.	sequence	function
TGA-element	Brassica oleracea	199	+	6	AACGAC	auxin-responsive element
TGA-element	Brassica oleracea	383	+	6	AACGAC	auxin-responsive element

+ TGACG-motif

Site Name	Organism	Position	Strand	Matrix score.	sequence	function
TGACG-motif	Hordeum vulgare	67	-	5	TGACG	cis-acting regulatory element involved in the MeJA-responsiveness
TGACG-motif	Hordeum vulgare	1051	+	5	TGACG	cis-acting regulatory element involved in the MeJA-responsiveness

Pahal.H02490 (*Panicum hallii*)

+ ABRE

Site Name	Organism	Position	Strand	Matrix score.	sequence	function
ABRE	Oryza sativa	283	+	9	GCCGCGTGGC	cis-acting element involved in the abscisic acid responsiveness
ABRE	Oryza sativa	964	-	9	GCCGCGTGGC	cis-acting element involved in the abscisic acid responsiveness

+ ARE

Site Name	Organism	Position	Strand	Matrix score.	sequence	function
ARE	Zea mays	417	-	6	TGGTTT	cis-acting regulatory element essential for the anaerobic induction

+ CAT-box

Site Name	Organism	Position	Strand	Matrix score.	sequence	function
CAT-box	Arabidopsis thaliana	487	-	6	GCCACT	cis-acting regulatory element related to meristem expression

+ CCGTCC-box

Site Name	Organism	Position	Strand	Matrix score.	sequence	function
CCGTCC-box	Arabidopsis thaliana	983	-	6	CCGTCC	cis-acting regulatory element related to meristem specific activation

+ CGTCA-motif

Site Name	Organism	Position	Strand	Matrix score.	sequence	function
CGTCA-motif	Hordeum vulgare	1007	-	5	CGTCA	cis-acting regulatory element involved in the MeJA-responsiveness
CGTCA-motif	Hordeum vulgare	1035	+	5	CGTCA	cis-acting regulatory element involved in the MeJA-responsiveness

+ GC-motif

Site Name	Organism	Position	Strand	Matrix score.	sequence	function
GC-motif	Zea mays	1104	-	7	GCCCCGG	enhancer-like element involved in anoxic specific inducibility

+ HSE

Site Name	Organism	Position	Strand	Matrix score.	sequence	function
HSE	Brassica oleracea	1342	+	9	AAAAAATTTC	cis-acting element involved in heat stress responsiveness

+ Skn-1_motif

Site Name	Organism	Position	Strand	Matrix score.	sequence	function
Skn-1_motif	Oryza sativa	1117	+	5	GTCAT	cis-acting regulatory element required for endosperm expression

+ TC-rich repeats

Site Name	Organism	Position	Strand	Matrix score.	sequence	function
TC-rich repeats	Nicotiana tabacum	1339	-	9	ATTTTCTTCA	cis-acting element involved in defense and stress responsiveness

+ TGA-element

Site Name	Organism	Position	Strand	Matrix score.	sequence	function
TGA-element	Brassica oleracea	155	+	6	AACGAC	auxin-responsive element
TGA-element	Brassica oleracea	1293	-	6	AACGAC	auxin-responsive element
TGA-element	Brassica oleracea	339	+	6	AACGAC	auxin-responsive element

+ TGACG-motif

Site Name	Organism	Position	Strand	Matrix score.	sequence	function
TGACG-motif	Hordeum vulgare	1007	+	5	TGACG	cis-acting regulatory element involved in the MeJA-responsiveness
TGACG-motif	Hordeum vulgare	1035	-	5	TGACG	cis-acting regulatory element involved in the MeJA-responsiveness

Traes_4BL_3EA2CD6B0 (*Triticum aestivum*)

+ ARE

Site Name	Organism	Position	Strand	Matrix score.	sequence	function
ARE	Zea mays	188	+	6	TGGTTT	cis-acting regulatory element essential for the anaerobic induction

+ CAT-box

Site Name	Organism	Position	Strand	Matrix score.	sequence	function
CAT-box	Arabidopsis thaliana	222	+	6	GCCACT	cis-acting regulatory element related to meristem expression

+ CGTCA-motif

Site Name	Organism	Position	Strand	Matrix score.	sequence	function
CGTCA-motif	Hordeum vulgare	366	-	5	CGTCA	cis-acting regulatory element involved in the MeJA-responsiveness
CGTCA-motif	Hordeum vulgare	1329	+	5	CGTCA	cis-acting regulatory element involved in the MeJA-responsiveness
CGTCA-motif	Hordeum vulgare	1189	-	5	CGTCA	cis-acting regulatory element involved in the MeJA-responsiveness

+ ERE

Site Name	Organism	Position	Strand	Matrix score.	sequence	function
ERE	Dianthus caryophyllus	1424	+	8	ATTTCAAA	ethylene-responsive element

+ GARE-motif

Site Name	Organism	Position	Strand	Matrix score.	sequence	function
GARE-motif	Brassica oleracea	1026	+	7	AAACAGA	gibberellin-responsive element

+ HSE

Site Name	Organism	Position	Strand	Matrix score.	sequence	function
HSE	Brassica oleracea	1168	-	9	AAAAAATTTC	cis-acting element involved in heat stress responsiveness

+ LTR

Site Name	Organism	Position	Strand	Matrix score.	sequence	function
LTR	Hordeum vulgare	1000	+	6	CCGAAA	cis-acting element involved in low-temperature responsiveness

+ Skn-1_motif

Site Name	Organism	Position	Strand	Matrix score.	sequence	function
Skn-1_motif	Oryza sativa	362	+	5	GTCAT	cis-acting regulatory element required for endosperm expression
Skn-1_motif	Oryza sativa	1047	-	5	GTCAT	cis-acting regulatory element required for endosperm expression
Skn-1_motif	Oryza sativa	365	-	5	GTCAT	cis-acting regulatory element required for endosperm expression
Skn-1_motif	Oryza sativa	1125	-	5	GTCAT	cis-acting regulatory element required for endosperm expression

+ TCA-element

Site Name	Organism	Position	Strand	Matrix score.	sequence	function
TCA-element	Nicotiana tabacum	1435	-	9	CCATCTTTTT	cis-acting element involved in salicylic acid responsiveness

+ TGACG-motif

Site Name	Organism	Position	Strand	Matrix score.	sequence	function
TGACG-motif	Hordeum vulgare	366	+	5	TGACG	cis-acting regulatory element involved in the MeJA-responsiveness
TGACG-motif	Hordeum vulgare	1329	-	5	TGACG	cis-acting regulatory element involved in the MeJA-responsiveness
TGACG-motif	Hordeum vulgare	1189	+	5	TGACG	cis-acting regulatory element involved in the MeJA-responsiveness

Traes_4AS_74660DF99 (*Triticum aestivum*)

+ ABRE

Site Name	Organism	Position	Strand	Matrix score.	sequence	function
ABRE	Hordeum vulgare	364	+	9	CCTACGTGGC	cis-acting element involved in the abscisic acid responsiveness
ABRE	Arabidopsis thaliana	367	+	7	ACGTGGC	cis-acting element involved in the abscisic acid responsiveness

+ ARE

Site Name	Organism	Position	Strand	Matrix score.	sequence	function
ARE	Zea mays	408	+	6	TGGTTT	cis-acting regulatory element essential for the anaerobic induction

+ CAT-box

Site Name	Organism	Position	Strand	Matrix score.	sequence	function
CAT-box	Arabidopsis thaliana	1067	-	6	GCCACT	cis-acting regulatory element related to meristem expression
CAT-box	Arabidopsis thaliana	1091	-	6	GCCACT	cis-acting regulatory element related to meristem expression

+ ERE

Site Name	Organism	Position	Strand	Matrix score.	sequence	function
ERE	Dianthus caryophyllus	1232	+	8	ATTTCAAA	ethylene-responsive element

+ GARE-motif

Site Name	Organism	Position	Strand	Matrix score.	sequence	function
GARE-motif	Brassica oleracea	1240	+	7	AAACAGA	gibberellin-responsive element

+ HSE

Site Name	Organism	Position	Strand	Matrix score.	sequence	function
HSE	Brassica oleracea	1315	+	9	AAAAAATTTC	cis-acting element involved in heat stress responsiveness

+ MBS

Site Name	Organism	Position	Strand	Matrix score.	sequence	function
MBS	Arabidopsis thaliana	868	-	6	TAACTG	MYB binding site involved in drought-inducibility

MBS	Arabidopsis thaliana	646	+	6	CAACTG	MYB binding site involved in drought-inducibility

+ TC-rich repeats

Site Name	Organism	Position	Strand	Matrix score.	sequence	function
TC-rich repeats	Nicotiana tabacum	281	+	9	ATTTTCTCCA	cis-acting element involved in defense and stress responsiveness

+ as-2-box

Site Name	Organism	Position	Strand	Matrix score.	sequence	function
as-2-box	Nicotiana tabacum	754	-	9	GATAatGATG	involved in shoot-specific expression and light responsiveness

Bradi1g67320 (*Brachypodium distachyon*)

\+ ABRE

Site Name	Organism	Position	Strand	Matrix score.	sequence	function
ABRE	Arabidopsis thaliana	1276	-	6	TACGTG	cis-acting element involved in the abscisic acid responsiveness

\+ ARE

Site Name	Organism	Position	Strand	Matrix score.	sequence	function
ARE	Zea mays	865	+	6	TGGTTT	cis-acting regulatory element essential for the anaerobic induction
ARE	Zea mays	1048	-	6	TGGTTT	cis-acting regulatory element essential for the anaerobic induction

\+ Box-W1

Site Name	Organism	Position	Strand	Matrix score.	sequence	function
Box-W1	Petroselinum crispum	668	-	6	TTGACC	fungal elicitor responsive element

\+ CGTCA-motif

Site Name	Organism	Position	Strand	Matrix score.	sequence	function
CGTCA-motif	Hordeum vulgare	985	-	5	CGTCA	cis-acting regulatory element involved in the MeJA-responsiveness

\+ DRE

Site Name	Organism	Position	Strand	Matrix score.	sequence	function
DRE	Arabidopsis thaliana	1445	-	9	TACCGACAT	"cis-acting element involved in dehydration, low-temp, salt stresses

\+ GARE-motif

Site Name	Organism	Position	Strand	Matrix score.	sequence	function
GARE-motif	Brassica oleracea	509	-	7	AAACAGA	gibberellin-responsive element

\+ GC-motif

Site Name	Organism	Position	Strand	Matrix score.	sequence	function
GC-motif	Zea mays	315	-	7	GCCCCGG	enhancer-like element involved in anoxic specific inducibility

\+ HSE

Site Name	Organism	Position	Strand	Matrix score.	sequence	function
HSE	Brassica oleracea	1105	-	9	AAAAAATTTC	cis-acting element involved in heat stress responsiveness

+ Skn-1_motif

Site Name	Organism	Position	Strand	Matrix score.	sequence	function
Skn-1_motif	Oryza sativa	530	-	5	GTCAT	cis-acting regulatory element required for endosperm expression
Skn-1_motif	Oryza sativa	984	-	5	GTCAT	cis-acting regulatory element required for endosperm expression
Skn-1_motif	Oryza sativa	787	-	5	GTCAT	cis-acting regulatory element required for endosperm expression
Skn-1_motif	Oryza sativa	1185	-	5	GTCAT	cis-acting regulatory element required for endosperm expression

+ TCA-element

Site Name	Organism	Position	Strand	Matrix score.	sequence	function
TCA-element	Brassica oleracea	712	+	9	GAGAAGAATA	cis-acting element involved in salicylic acid responsiveness
TCA-element	Nicotiana tabacum	1020	+	9	CCATCTTTTT	cis-acting element involved in salicylic acid responsiveness
TCA-element	Brassica oleracea	715	+	9	CAGAAAAGGA	cis-acting element involved in salicylic acid responsiveness
TCA-element	Brassica oleracea	1069	-	9	GAGAAGAATA	cis-acting element involved in salicylic acid responsiveness

+ TGACG-motif

Site Name	Organism	Position	Strand	Matrix score.	sequence	function
TGACG-motif	Hordeum vulgare	985	+	5	TGACG	cis-acting regulatory element involved in the MeJA-responsiveness

+ as-2-box

Site Name	Organism	Position	Strand	Matrix score.	sequence	function
as-2-box	Nicotiana tabacum	24	+	9	GATAatGATG	involved in shoot-specific expression and light responsiveness

Brast02G124800 (*Brachypodium stacei*)

+ ABRE

Site Name	Organism	Position	Strand	Matrix score.	sequence	function
ABRE	Hordeum vulgare	43	+	9	CGTACGTGCA	cis-acting element involved in the abscisic acid responsiveness
ABRE	Oryza sativa	1048	+	9	GCCGCGTGGC	cis-acting element involved in the abscisic acid responsiveness
ABRE	Arabidopsis thaliana	45	+	6	CACGTG	cis-acting element involved in the abscisic acid responsiveness

+ ARE

Site Name	Organism	Position	Strand	Matrix score.	sequence	function
ARE	Zea mays	345	+	6	TGGTTT	cis-acting regulatory element essential for the anaerobic induction

+ CAT-box

Site Name	Organism	Position	Strand	Matrix score.	sequence	function
CAT-box	Arabidopsis thaliana	733	+	6	GCCACT	cis-acting regulatory element related to meristem expression
CAT-box	Arabidopsis thaliana	782	+	6	GCCACT	cis-acting regulatory element related to meristem expression

+ CGTCA-motif

Site Name	Organism	Position	Strand	Matrix score.	sequence	function
CGTCA-motif	Hordeum vulgare	865	-	5	CGTCA	cis-acting regulatory element involved in the MeJA-responsiveness

+ DRE

Site Name	Organism	Position	Strand	Matrix score.	sequence	function
DRE	Arabidopsis thaliana	1448	-	9	TACCGACAT	"cis-acting element involved in dehydration, low-temp, salt stresses

+ GARE-motif

Site Name	Organism	Position	Strand	Matrix score.	sequence	function
GARE-motif	Brassica oleracea	1488	+	7	AAACAGA	gibberellin-responsive element

+ GC-motif

Site Name	Organism	Position	Strand	Matrix score.	sequence	function

GC-motif	Zea mays	1049	-	8	CCACGGGG	enhancer-like element involved in anoxic specific inducibility

+ HSE

Site Name	Organism	Position	Strand	Matrix score.	sequence	function
HSE	Brassica oleracea	590	+	9	AAAAAATTTC	cis-acting element involved in heat stress responsiveness
HSE	Brassica oleracea	1184	+	9	AGAAAATTCG	cis-acting element involved in heat stress responsiveness
HSE	Brassica oleracea	700	+	9	AAAAAATTTC	cis-acting element involved in heat stress responsiveness

+ MBS

Site Name	Organism	Position	Strand	Matrix score.	sequence	function
MBS	Arabidopsis thaliana	298	+	6	CAACTG	MYB binding site involved in drought-inducibility

+ P-box

Site Name	Organism	Position	Strand	Matrix score.	sequence	function
P-box	Oryza sativa	1236	-	7	CCTTTTG	gibberellin-responsive element

+ Skn-1_motif

Site Name	Organism	Position	Strand	Matrix score.	sequence	function
Skn-1_motif	Oryza sativa	864	-	5	GTCAT	cis-acting regulatory element required for endosperm expression

+ TC-rich repeats

Site Name	Organism	Position	Strand	Matrix score.	sequence	function
TC-rich repeats	Nicotiana tabacum	1387	-	9	ATTTTCTCCA	cis-acting element involved in defense and stress responsiveness

+ TCA-element

Site Name	Organism	Position	Strand	Matrix score.	sequence	function
TCA-element	Nicotiana tabacum	802	-	10	CCATCTTTTT	cis-acting element involved in salicylic acid responsiveness

+ TGACG-motif

Site Name	Organism	Position	Strand	Matrix score.	sequence	function
TGACG-motif	Hordeum vulgare	865	+	5	TGACG	cis-acting regulatory element involved in the MeJA-responsiveness

LOC_Os03g15700 (*Oryza sativa*)

+ ARE

Site Name	Organism	Position	Strand	Matrix score.	sequence	function
ARE	Zea mays	25	+	6	TGGTTT	cis-acting regulatory element essential for the anaerobic induction
ARE	Zea mays	1441	-	6	TGGTTT	cis-acting regulatory element essential for the anaerobic induction

+ CAT-box

Site Name	Organism	Position	Strand	Matrix score.	sequence	function
CAT-box	Arabidopsis thaliana	1087	+	6	GCCACT	cis-acting regulatory element related to meristem expression

+ CGTCA-motif

Site Name	Organism	Position	Strand	Matrix score.	sequence	function
CGTCA-motif	Hordeum vulgare	210	-	5	CGTCA	cis-acting regulatory element involved in the MeJA-responsiveness
CGTCA-motif	Hordeum vulgare	979	+	5	CGTCA	cis-acting regulatory element involved in the MeJA-responsiveness

+ GCN4_motif

Site Name	Organism	Position	Strand	Matrix score.	sequence	function
GCN4_motif	Oryza sativa	739	-	7	TGAGTCA	cis-regulatory element involved in endosperm expression
GCN4_motif	Oryza sativa	877	-	7	TGAGTCA	cis-regulatory element involved in endosperm expression

+ LTR

Site Name	Organism	Position	Strand	Matrix score.	sequence	function
LTR	Hordeum vulgare	277	-	6	CCGAAA	cis-acting element involved in low-temperature responsiveness

+ RY-element

Site Name	Organism	Position	Strand	Matrix score.	sequence	function
RY-element	Helianthus annuus	180	+	8	CATGCATG	cis-acting regulatory element involved in seed-specific regulation

+ Skn-1_motif

Site Name	Organism	Position	Strand	Matrix score.	sequence	function

Skn-1_motif	Oryza sativa	209	-	5	GTCAT	cis-acting regulatory element required for endosperm expression
Skn-1_motif	Oryza sativa	704	-	5	GTCAT	cis-acting regulatory element required for endosperm expression
Skn-1_motif	Oryza sativa	417	+	5	GTCAT	cis-acting regulatory element required for endosperm expression
Skn-1_motif	Oryza sativa	738	-	5	GTCAT	cis-acting regulatory element required for endosperm expression

+ TGACG-motif

Site Name	Organism	Position	Strand	Matrix score.	sequence	function
TGACG-motif	Hordeum vulgare	210	+	5	TGACG	cis-acting regulatory element involved in the MeJA-responsiveness
TGACG-motif	Hordeum vulgare	979	-	5	TGACG	cis-acting regulatory element involved in the MeJA-responsiveness

+ as-2-box

Site Name	Organism	Position	Strand	Matrix score.	sequence	function
as-2-box	Nicotiana tabacum	764	+	9	GATAatGATG	involved in shoot-specific expression and light responsiveness

Oropetium_20150105_00778 (*Oropetium thomaeum*)

+ ABRE

Site Name	Organism	Position	Strand	Matrix score.	sequence	function
ABRE	Oryza sativa	223	+	9	GCCGCGTGGC	cis-acting element involved in the abscisic acid responsiveness

+ ARE

Site Name	Organism	Position	Strand	Matrix score.	sequence	function
ARE	Zea mays	1329	+	6	TGGTTT	cis-acting regulatory element essential for the anaerobic induction

+ CCGTCC-box

Site Name	Organism	Position	Strand	Matrix score.	sequence	function
CCGTCC-box	Arabidopsis thaliana	928	+	6	CCGTCC	cis-acting regulatory element related to meristem specific activation
CCGTCC-box	Arabidopsis thaliana	1072	+	6	CCGTCC	cis-acting regulatory element related to meristem specific activation

+ CGTCA-motif

Site Name	Organism	Position	Strand	Matrix score.	sequence	function
CGTCA-motif	Hordeum vulgare	662	-	5	CGTCA	cis-acting regulatory element involved in the MeJA-responsiveness
CGTCA-motif	Hordeum vulgare	1259	+	5	CGTCA	cis-acting regulatory element involved in the MeJA-responsiveness
CGTCA-motif	Hordeum vulgare	883	-	5	CGTCA	cis-acting regulatory element involved in the MeJA-responsiveness
CGTCA-motif	Hordeum vulgare	672	+	5	CGTCA	cis-acting regulatory element involved in the MeJA-responsiveness
CGTCA-motif	Hordeum vulgare	898	-	5	CGTCA	cis-acting regulatory element involved in the MeJA-responsiveness

+ ELI-box3

Site Name	Organism	Position	Strand	Matrix score.	sequence	function
ELI-box3	Brassica oleracea	1326	-	9	AAACCAATT	elicitor-responsive element

+ GC-motif

Site Name	Organism	Position	Strand	Matrix score.	sequence	function
GC-motif	Zea mays	981	-	6	CCCCCG	enhancer-like element involved in anoxic

specific inducibility

+ MBS

Site Name	Organism	Position	Strand	Matrix score.	sequence	function
MBS	Arabidopsis thaliana	1220	-	6	TAACTG	MYB binding site involved in drought-inducibility

+ Skn-1_motif

Site Name	Organism	Position	Strand	Matrix score.	sequence	function
Skn-1_motif	Oryza sativa	491	-	5	GTCAT	cis-acting regulatory element required for endosperm expression
Skn-1_motif	Oryza sativa	882	-	5	GTCAT	cis-acting regulatory element required for endosperm expression
Skn-1_motif	Oryza sativa	646	+	5	GTCAT	cis-acting regulatory element required for endosperm expression
Skn-1_motif	Oryza sativa	897	-	5	GTCAT	cis-acting regulatory element required for endosperm expression

+ TC-rich repeats

Site Name	Organism	Position	Strand	Matrix score.	sequence	function
TC-rich repeats	Nicotiana tabacum	1212	+	9	ATTTTCTTCA	cis-acting element involved in defense and stress responsiveness

+ TGA-element

Site Name	Organism	Position	Strand	Matrix score.	sequence	function
TGA-element	Brassica oleracea	207	-	6	AACGAC	auxin-responsive element
TGA-element	Brassica oleracea	279	+	6	AACGAC	auxin-responsive element

+ TGACG-motif

Site Name	Organism	Position	Strand	Matrix score.	sequence	function
TGACG-motif	Hordeum vulgare	662	+	5	TGACG	cis-acting regulatory element involved in the MeJA-responsiveness
TGACG-motif	Hordeum vulgare	1259	-	5	TGACG	cis-acting regulatory element involved in the MeJA-responsiveness
TGACG-motif	Hordeum vulgare	883	+	5	TGACG	cis-acting regulatory element involved in the MeJA-responsiveness
TGACG-motif	Hordeum vulgare	672	-	5	TGACG	cis-acting regulatory element involved in the MeJA-responsiveness

TGACG-motif	Hordeum vulgare	898	+	5	TGACG	cis-acting regulatory element involved in the MeJA-responsiveness

+ motif IIb

Site Name	Organism	Position	Strand	Matrix score.	sequence	function
motif IIb	Oryza sativa	217	-	9	CCGCCGCGCT	abscisic acid responsive element

Seita.9G455700 (*Setaria italica*)

+ ABRE

Site Name	Organism	Position	Strand	Matrix score.	sequence	function
ABRE	Oryza sativa	257	+	9	GCCGCGTGGC	cis-acting element involved in the abscisic acid responsiveness
ABRE	Oryza sativa	907	-	9	GCCGCGTGGC	cis-acting element involved in the abscisic acid responsiveness

+ Box-W1

Site Name	Organism	Position	Strand	Matrix score.	sequence	function
Box-W1	Petroselinum crispum	726	-	6	TTGACC	fungal elicitor responsive element

+ CCGTCC-box

Site Name	Organism	Position	Strand	Matrix score.	sequence	function
CCGTCC-box	Arabidopsis thaliana	926	-	6	CCGTCC	cis-acting regulatory element related to meristem specific activation
CCGTCC-box	Arabidopsis thaliana	1139	+	6	CCGTCC	cis-acting regulatory element related to meristem specific activation

+ CGTCA-motif

Site Name	Organism	Position	Strand	Matrix score.	sequence	function
CGTCA-motif	Hordeum vulgare	950	-	5	CGTCA	cis-acting regulatory element involved in the MeJA-responsiveness

+ GC-motif

Site Name	Organism	Position	Strand	Matrix score.	sequence	function
GC-motif	Zea mays	1047	-	7	GCCCCGG	enhancer-like element involved in anoxic specific inducibility

+ GCN4_motif

Site Name	Organism	Position	Strand	Matrix score.	sequence	function
GCN4_motif	Oryza sativa	61	-	7	TGTGTCA	cis-regulatory element involved in endosperm expression
GCN4_motif	Oryza sativa	702	+	7	CAAGCCA	cis-regulatory element involved in endosperm expression

+ MBS

Site Name	Organism	Position	Strand	Matrix score.	sequence	function

MBS	Arabidopsis thaliana	1385	-	6	TAACTG	MYB binding site involved in drought-inducibility

\+ Skn-1_motif

Site Name	Organism	Position	Strand	Matrix score.	sequence	function
Skn-1_motif	Oryza sativa	537	-	5	GTCAT	cis-acting regulatory element required for endosperm expression
Skn-1_motif	Oryza sativa	1235	+	5	GTCAT	cis-acting regulatory element required for endosperm expression

\+ TGA-element

Site Name	Organism	Position	Strand	Matrix score.	sequence	function
TGA-element	Brassica oleracea	129	+	6	AACGAC	auxin-responsive element
TGA-element	Brassica oleracea	563	-	6	AACGAC	auxin-responsive element
TGA-element	Brassica oleracea	313	+	6	AACGAC	auxin-responsive element

\+ TGACG-motif

Site Name	Organism	Position	Strand	Matrix score.	sequence	function
TGACG-motif	Hordeum vulgare	950	+	5	TGACG	cis-acting regulatory element involved in the MeJA-responsiveness

Sevir.9G459400 (*Setaria viridis*)

+ ABRE

Site Name	Organism	Position	Strand	Matrix score.	sequence	function
ABRE	Oryza sativa	157	+	9	GCCGCGTGGC	cis-acting element involved in the abscisic acid responsiveness
ABRE	Oryza sativa	807	-	9	GCCGCGTGGC	cis-acting element involved in the abscisic acid responsiveness

+ Box-W1

Site Name	Organism	Position	Strand	Matrix score.	sequence	function
Box-W1	Petroselinum crispum	626	-	6	TTGACC	fungal elicitor responsive element

+ CCGTCC-box

Site Name	Organism	Position	Strand	Matrix score.	sequence	function
CCGTCC-box	Arabidopsis thaliana	826	-	6	CCGTCC	cis-acting regulatory element related to meristem specific activation
CCGTCC-box	Arabidopsis thaliana	1039	+	6	CCGTCC	cis-acting regulatory element related to meristem specific activation

+ CGTCA-motif

Site Name	Organism	Position	Strand	Matrix score.	sequence	function
CGTCA-motif	Hordeum vulgare	850	-	5	CGTCA	cis-acting regulatory element involved in the MeJA-responsiveness
CGTCA-motif	Hordeum vulgare	1122	+	5	CGTCA	cis-acting regulatory element involved in the MeJA-responsiveness

+ GC-motif

Site Name	Organism	Position	Strand	Matrix score.	sequence	function
GC-motif	Zea mays	947	-	7	GCCCCGG	enhancer-like element involved in anoxic specific inducibility

+ GCN4_motif

Site Name	Organism	Position	Strand	Matrix score.	sequence	function
GCN4_motif	Oryza sativa	602	+	7	CAAGCCA	cis-regulatory element involved in endosperm expression

+ MBS

Site Name	Organism	Position	Strand	Matrix score.	sequence	function

MBS	Arabidopsis thaliana	1395	-	6	TAACTG	MYB binding site involved in drought-inducibility

+ Skn-1_motif

Site Name	Organism	Position	Strand	Matrix score.	sequence	function
Skn-1_motif	Oryza sativa	437	-	5	GTCAT	cis-acting regulatory element required for endosperm expression
Skn-1_motif	Oryza sativa	1245	+	5	GTCAT	cis-acting regulatory element required for endosperm expression
Skn-1_motif	Oryza sativa	1123	+	5	GTCAT	cis-acting regulatory element required for endosperm expression

+ TGA-element

Site Name	Organism	Position	Strand	Matrix score.	sequence	function
TGA-element	Brassica oleracea	29	+	6	AACGAC	auxin-responsive element
TGA-element	Brassica oleracea	463	-	6	AACGAC	auxin-responsive element
TGA-element	Brassica oleracea	213	+	6	AACGAC	auxin-responsive element

+ TGACG-motif

Site Name	Organism	Position	Strand	Matrix score.	sequence	function
TGACG-motif	Hordeum vulgare	850	+	5	TGACG	cis-acting regulatory element involved in the MeJA-responsiveness
TGACG-motif	Hordeum vulgare	1122	-	5	TGACG	cis-acting regulatory element involved in the MeJA-responsiveness

Aco024234 (*Ananas comosus*)

+ ARE

Site Name	Organism	Position	Strand	Matrix score.	sequence	function
ARE	Zea mays	244	+	6	TGGTTT	cis-acting regulatory element essential for the anaerobic induction
ARE	Zea mays	775	-	6	TGGTTT	cis-acting regulatory element essential for the anaerobic induction
ARE	Zea mays	418	+	6	TGGTTT	cis-acting regulatory element essential for the anaerobic induction
ARE	Zea mays	1114	-	6	TGGTTT	cis-acting regulatory element essential for the anaerobic induction
ARE	Zea mays	411	+	6	TGGTTT	cis-acting regulatory element essential for the anaerobic induction
ARE	Zea mays	452	-	6	TGGTTT	cis-acting regulatory element essential for the anaerobic induction

+ Box-W1

Site Name	Organism	Position	Strand	Matrix score.	sequence	function
Box-W1	Petroselinum crispum	639	-	6	TTGACC	fungal elicitor responsive element
Box-W1	Petroselinum crispum	1487	+	6	TTGACC	fungal elicitor responsive element

+ CGTCA-motif

Site Name	Organism	Position	Strand	Matrix score.	sequence	function
CGTCA-motif	Hordeum vulgare	1398	-	5	CGTCA	cis-acting regulatory element involved in the MeJA-responsiveness

+ ERE

Site Name	Organism	Position	Strand	Matrix score.	sequence	function
ERE	Dianthus caryophyllus	845	+	8	ATTTCAAA	ethylene-responsive element

+ HSE

Site Name	Organism	Position	Strand	Matrix score.	sequence	function
HSE	Brassica oleracea	373	+	9	AAAAAATTTC	cis-acting element involved in heat stress responsiveness
HSE	Brassica oleracea	1374	-	9	AAAAAATTTC	cis-acting element involved in heat stress responsiveness

HSE	Brassica oleracea	1181	-	9	AAAAAATTTC	cis-acting element involved in heat stress responsiveness
HSE	Brassica oleracea	714	-	9	AAAAAATTTC	cis-acting element involved in heat stress responsiveness
HSE	Brassica oleracea	1280	-	9	AAAAAATTTC	cis-acting element involved in heat stress responsiveness

+ LTR

Site Name	Organism	Position	Strand	Matrix score.	sequence	function
LTR	Hordeum vulgare	1143	+	6	CCGAAA	cis-acting element involved in low-temperature responsiveness
LTR	Hordeum vulgare	1437	-	6	CCGAAA	cis-acting element involved in low-temperature responsiveness
LTR	Hordeum vulgare	1155	-	6	CCGAAA	cis-acting element involved in low-temperature responsiveness

+ SARE

Site Name	Organism	Position	Strand	Matrix score.	sequence	function
SARE	Nicotiana tabacum	591	+	11	TTCGACCATCTT	cis-acting element involved in salicylic acid responsiveness

+ Skn-1_motif

Site Name	Organism	Position	Strand	Matrix score.	sequence	function
Skn-1_motif	Oryza sativa	304	-	5	GTCAT	cis-acting regulatory element required for endosperm expression

+ TCA-element

Site Name	Organism	Position	Strand	Matrix score.	sequence	function
TCA-element	Nicotiana tabacum	596	+	9	CCATCTTTTT	cis-acting element involved in salicylic acid responsiveness

+ TGACG-motif

Site Name	Organism	Position	Strand	Matrix score.	sequence	function
TGACG-motif	Hordeum vulgare	1398	+	5	TGACG	cis-acting regulatory element involved in the MeJA-responsiveness

180432 (*Selaginella moellendorffii*)

+ ABRE

Site Name	Organism	Position	Strand	Matrix score.	sequence	function
ABRE	Arabidopsis thaliana	866	-	6	CACGTG	cis-acting element involved in the abscisic acid responsiveness

+ Box-W1

Site Name	Organism	Position	Strand	Matrix score.	sequence	function
Box-W1	Petroselinum crispum	475	+	6	TTGACC	fungal elicitor responsive element

+ CAT-box

Site Name	Organism	Position	Strand	Matrix score.	sequence	function
CAT-box	Arabidopsis thaliana	1027	+	6	GCCACT	cis-acting regulatory element related to meristem expression

+ CCGTCC-box

Site Name	Organism	Position	Strand	Matrix score.	sequence	function
CCGTCC-box	Arabidopsis thaliana	818	-	6	CCGTCC	cis-acting regulatory element related to meristem specific activation
CCGTCC-box	Arabidopsis thaliana	1391	-	6	CCGTCC	cis-acting regulatory element related to meristem specific activation
CCGTCC-box	Arabidopsis thaliana	1338	-	6	CCGTCC	cis-acting regulatory element related to meristem specific activation
CCGTCC-box	Arabidopsis thaliana	1416	-	6	CCGTCC	cis-acting regulatory element related to meristem specific activation

+ CGTCA-motif

Site Name	Organism	Position	Strand	Matrix score.	sequence	function
CGTCA-motif	Hordeum vulgare	1473	+	5	CGTCA	cis-acting regulatory element involved in the MeJA-responsiveness

+ ERE

Site Name	Organism	Position	Strand	Matrix score.	sequence	function
ERE	Dianthus caryophyllus	1407	-	8	ATTTCAAA	ethylene-responsive element

+ HSE

Site Name	Organism	Position	Strand	Matrix score.	sequence	function

HSE	Brassica oleracea	425	-	9	AAAAAATTTC	cis-acting element involved in heat stress responsiveness

+ Skn-1_motif

Site Name	Organism	Position	Strand	Matrix score.	sequence	function
Skn-1_motif	Oryza sativa	195	+	5	GTCAT	cis-acting regulatory element required for endosperm expression
Skn-1_motif	Oryza sativa	850	+	5	GTCAT	cis-acting regulatory element required for endosperm expression
Skn-1_motif	Oryza sativa	645	+	5	GTCAT	cis-acting regulatory element required for endosperm expression
Skn-1_motif	Oryza sativa	853	-	5	GTCAT	cis-acting regulatory element required for endosperm expression

+ TC-rich repeats

Site Name	Organism	Position	Strand	Matrix score.	sequence	function
TC-rich repeats	Nicotiana tabacum	997	-	9	GTTTTCTTAC	cis-acting element involved in defense and stress responsiveness

+ TCA-element

Site Name	Organism	Position	Strand	Matrix score.	sequence	function
TCA-element	Brassica oleracea	322	-	9	TCAGAAGAGG	cis-acting element involved in salicylic acid responsiveness
TCA-element	Brassica oleracea	410	+	9	GAGAAGAATA	cis-acting element involved in salicylic acid responsiveness

+ TGA-element

Site Name	Organism	Position	Strand	Matrix score.	sequence	function
TGA-element	Brassica oleracea	272	-	6	AACGAC	auxin-responsive element

+ TGACG-motif

Site Name	Organism	Position	Strand	Matrix score.	sequence	function
TGACG-motif	Hordeum vulgare	1473	-	5	TGACG	cis-acting regulatory element involved in the MeJA-responsiveness

Pp3c9_13030 (*Physcomitrella patens*)

+ ARE

Site Name	Organism	Position	Strand	Matrix score.	sequence	function
ARE	Zea mays	391	-	6	TGGTTT	cis-acting regulatory element essential for the anaerobic induction
ARE	Zea mays	1388	-	6	TGGTTT	cis-acting regulatory element essential for the anaerobic induction

+ Box-W1

Site Name	Organism	Position	Strand	Matrix score.	sequence	function
Box-W1	Petroselinum crispum	615	+	6	TTGACC	fungal elicitor responsive element
Box-W1	Petroselinum crispum	680	+	6	TTGACC	fungal elicitor responsive element

+ CGTCA-motif

Site Name	Organism	Position	Strand	Matrix score.	sequence	function
CGTCA-motif	Hordeum vulgare	1142	-	5	CGTCA	cis-acting regulatory element involved in the MeJA-responsiveness
CGTCA-motif	Hordeum vulgare	1414	-	5	CGTCA	cis-acting regulatory element involved in the MeJA-responsiveness

+ GARE-motif

Site Name	Organism	Position	Strand	Matrix score.	sequence	function
GARE-motif	Brassica oleracea	1392	-	7	TCTGTTG	gibberellin-responsive element

+ GC-motif

Site Name	Organism	Position	Strand	Matrix score.	sequence	function
GC-motif	Zea mays	1473	+	6	CCCCCG	enhancer-like element involved in anoxic specific inducibility

+ MBS

Site Name	Organism	Position	Strand	Matrix score.	sequence	function
MBS	Arabidopsis thaliana	1269	-	6	CAACTG	MYB binding site involved in drought-inducibility

+ P-box

Site Name	Organism	Position	Strand	Matrix score.	sequence	function

P-box	Oryza sativa	676	+	7	CCTTTTG	gibberellin-responsive element

+ Skn-1_motif

Site Name	Organism	Position	Strand	Matrix score.	sequence	function
Skn-1_motif	Oryza sativa	583	-	5	GTCAT	cis-acting regulatory element required for endosperm expression
Skn-1_motif	Oryza sativa	1413	-	5	GTCAT	cis-acting regulatory element required for endosperm expression

+ TCA-element

Site Name	Organism	Position	Strand	Matrix score.	sequence	function
TCA-element	Nicotiana tabacum	446	-	9	CCATCTTTTT	cis-acting element involved in salicylic acid responsiveness

+ TGA-element

Site Name	Organism	Position	Strand	Matrix score.	sequence	function
TGA-element	Brassica oleracea	333	+	6	AACGAC	auxin-responsive element
TGA-element	Brassica oleracea	1401	+	6	AACGAC	auxin-responsive element

+ TGACG-motif

Site Name	Organism	Position	Strand	Matrix score.	sequence	function
TGACG-motif	Hordeum vulgare	1142	+	5	TGACG	cis-acting regulatory element involved in the MeJA-responsiveness
TGACG-motif	Hordeum vulgare	1414	+	5	TGACG	cis-acting regulatory element involved in the MeJA-responsiveness

Pp3c15_8160 (*Physcomitrella patens*)

+ ARE

Site Name	Organism	Position	Strand	Matrix score.	sequence	function
ARE	Zea mays	606	+	6	TGGTTT	cis-acting regulatory element essential for the anaerobic induction
ARE	Zea mays	913	+	6	TGGTTT	cis-acting regulatory element essential for the anaerobic induction

+ Box-W1

Site Name	Organism	Position	Strand	Matrix score.	sequence	function
Box-W1	Petroselinum crispum	665	+	6	TTGACC	fungal elicitor responsive element

+ CGTCA-motif

Site Name	Organism	Position	Strand	Matrix score.	sequence	function
CGTCA-motif	Hordeum vulgare	307	-	5	CGTCA	cis-acting regulatory element involved in the MeJA-responsiveness

+ GCN4_motif

Site Name	Organism	Position	Strand	Matrix score.	sequence	function
GCN4_motif	Oryza sativa	1381	-	7	TGTGTCA	cis-regulatory element involved in endosperm expression

+ HSE

Site Name	Organism	Position	Strand	Matrix score.	sequence	function
HSE	Brassica oleracea	230	+	9	AGAAAATTCG	cis-acting element involved in heat stress responsiveness
HSE	Brassica oleracea	1146	-	9	AAAAAATTTC	cis-acting element involved in heat stress responsiveness

+ LTR

Site Name	Organism	Position	Strand	Matrix score.	sequence	function
LTR	Hordeum vulgare	38	-	6	CCGAAA	cis-acting element involved in low-temperature responsiveness

+ MBS

Site Name	Organism	Position	Strand	Matrix score.	sequence	function
MBS	Arabidopsis thaliana	899	-	6	TAACTG	MYB binding site involved in drought-inducibility

+ Skn-1_motif

Site Name	Organism	Position	Strand	Matrix score.	sequence	function
Skn-1_motif	Oryza sativa	306	-	5	GTCAT	cis-acting regulatory element required for endosperm expression
Skn-1_motif	Oryza sativa	1380	-	5	GTCAT	cis-acting regulatory element required for endosperm expression

+ TC-rich repeats

Site Name	Organism	Position	Strand	Matrix score.	sequence	function
TC-rich repeats	Nicotiana tabacum	613	+	9	ATTTTCTCCA	cis-acting element involved in defense and stress responsiveness
TC-rich repeats	Nicotiana tabacum	1292	+	9	ATTTTCTTCA	cis-acting element involved in defense and stress responsiveness

+ TGACG-motif

Site Name	Organism	Position	Strand	Matrix score.	sequence	function
TGACG-motif	Hordeum vulgare	307	+	5	TGACG	cis-acting regulatory element involved in the MeJA-responsiveness

Sphfalx0006s0301 (*Sphagnum fallax*)

+ ARE

Site Name	Organism	Position	Strand	Matrix score.	sequence	function
ARE	Zea mays	352	+	6	TGGTTT	cis-acting regulatory element essential for the anaerobic induction

+ AuxRR-core

Site Name	Organism	Position	Strand	Matrix score.	sequence	function
AuxRR-core	Nicotiana tabacum	1003	+	7	GGTCCAT	cis-acting regulatory element involved in auxin responsiveness

+ Box-W1

Site Name	Organism	Position	Strand	Matrix score.	sequence	function
Box-W1	Petroselinum crispum	139	-	6	TTGACC	fungal elicitor responsive element
Box-W1	Petroselinum crispum	221	-	6	TTGACC	fungal elicitor responsive element
Box-W1	Petroselinum crispum	178	-	6	TTGACC	fungal elicitor responsive element
Box-W1	Petroselinum crispum	1222	-	6	TTGACC	fungal elicitor responsive element

+ CCGTCC-box

Site Name	Organism	Position	Strand	Matrix score.	sequence	function
CCGTCC-box	Arabidopsis thaliana	345	-	6	CCGTCC	cis-acting regulatory element related to meristem specific activation

+ CGTCA-motif

Site Name	Organism	Position	Strand	Matrix score.	sequence	function
CGTCA-motif	Hordeum vulgare	85	-	5	CGTCA	cis-acting regulatory element involved in the MeJA-responsiveness
CGTCA-motif	Hordeum vulgare	913	+	5	CGTCA	cis-acting regulatory element involved in the MeJA-responsiveness
CGTCA-motif	Hordeum vulgare	673	-	5	CGTCA	cis-acting regulatory element involved in the MeJA-responsiveness
CGTCA-motif	Hordeum vulgare	943	-	5	CGTCA	cis-acting regulatory element involved in the MeJA-responsiveness

+ GARE-motif

Site Name	Organism	Position	Strand	Matrix	sequence	function

				score.		
GARE-motif	Brassica oleracea	917	+	7	AAACAGA	gibberellin-responsive element

+ GCN4_motif

Site Name	Organism	Position	Strand	Matrix score.	sequence	function
GCN4_motif	Oryza sativa	1404	+	7	TGTGTCA	cis-regulatory element involved in endosperm expression

+ MBS

Site Name	Organism	Position	Strand	Matrix score.	sequence	function
MBS	Arabidopsis thaliana	387	-	6	TAACTG	MYB binding site involved in drought-inducibility

+ P-box

Site Name	Organism	Position	Strand	Matrix score.	sequence	function
P-box	Pisum sativum	87	-	11	GACCAAACTCGT	gibberellin-responsive element

+ Skn-1_motif

Site Name	Organism	Position	Strand	Matrix score.	sequence	function
Skn-1_motif	Oryza sativa	84	-	5	GTCAT	cis-acting regulatory element required for endosperm expression
Skn-1_motif	Oryza sativa	672	-	5	GTCAT	cis-acting regulatory element required for endosperm expression
Skn-1_motif	Oryza sativa	96	+	5	GTCAT	cis-acting regulatory element required for endosperm expression
Skn-1_motif	Oryza sativa	747	-	5	GTCAT	cis-acting regulatory element required for endosperm expression

+ TC-rich repeats

Site Name	Organism	Position	Strand	Matrix score.	sequence	function
TC-rich repeats	Nicotiana tabacum	76	-	9	ATTTTCTCCA	cis-acting element involved in defense and stress responsiveness
TC-rich repeats	Nicotiana tabacum	230	+	9	ATTTTCTTCA	cis-acting element involved in defense and stress responsiveness

+ TCA-element

Site Name	Organism	Position	Strand	Matrix score.	sequence	function
TCA-element	Brassica oleracea	653	+	9	CAGAAAAGGA	cis-acting element involved in salicylic acid responsiveness

+ TGACG-motif

Site Name	Organism	Position	Strand	Matrix score.	sequence	function
TGACG-motif	Hordeum vulgare	85	+	5	TGACG	cis-acting regulatory element involved in the MeJA-responsiveness
TGACG-motif	Hordeum vulgare	913	-	5	TGACG	cis-acting regulatory element involved in the MeJA-responsiveness
TGACG-motif	Hordeum vulgare	673	+	5	TGACG	cis-acting regulatory element involved in the MeJA-responsiveness
TGACG-motif	Hordeum vulgare	943	+	5	TGACG	cis-acting regulatory element involved in the MeJA-responsiveness

+ dOCT

Site Name	Organism	Position	Strand	Matrix score.	sequence	function
dOCT	Arabidopsis thaliana	580	-	8	CaCGGATC	cis-acting regulatory element related to meristem specific activation

Sphfalx0014s0238 (*Sphagnum fallax*)

+ ARE

Site Name	Organism	Position	Strand	Matrix score.	sequence	function
ARE	Zea mays	127	+	6	TGGTTT	cis-acting regulatory element essential for the anaerobic induction

+ Box-W1

Site Name	Organism	Position	Strand	Matrix score.	sequence	function
Box-W1	Petroselinum crispum	518	-	6	TTGACC	fungal elicitor responsive element
Box-W1	Petroselinum crispum	972	+	6	TTGACC	fungal elicitor responsive element

+ CCGTCC-box

Site Name	Organism	Position	Strand	Matrix score.	sequence	function
CCGTCC-box	Arabidopsis thaliana	1264	+	6	CCGTCC	cis-acting regulatory element related to meristem specific activation

+ CGTCA-motif

Site Name	Organism	Position	Strand	Matrix score.	sequence	function
CGTCA-motif	Hordeum vulgare	862	-	5	CGTCA	cis-acting regulatory element involved in the MeJA-responsiveness

+ GARE-motif

Site Name	Organism	Position	Strand	Matrix score.	sequence	function
GARE-motif	Brassica oleracea	1221	+	7	AAACAGA	gibberellin-responsive element

+ HSE

Site Name	Organism	Position	Strand	Matrix score.	sequence	function
HSE	Lycopersicon esculentum	611	+	8	CNNGAANNTTCNNG	cis-acting element involved in heat stress responsiveness

+ MBS

Site Name	Organism	Position	Strand	Matrix score.	sequence	function
MBS	Arabidopsis thaliana	162	-	6	TAACTG	MYB binding site involved in drought-inducibility
MBS	Arabidopsis thaliana	793	+	6	CAACTG	MYB binding site involved in drought-inducibility

+ Skn-1_motif

Site Name	Organism	Position	Strand	Matrix score.	sequence	function
Skn-1_motif	Oryza sativa	36	-	5	GTCAT	cis-acting regulatory element required for endosperm expression
Skn-1_motif	Oryza sativa	1049	+	5	GTCAT	cis-acting regulatory element required for endosperm expression
Skn-1_motif	Oryza sativa	861	-	5	GTCAT	cis-acting regulatory element required for endosperm expression

+ TGA-element

Site Name	Organism	Position	Strand	Matrix score.	sequence	function
TGA-element	Brassica oleracea	228	+	6	AACGAC	auxin-responsive element

+ TGACG-motif

Site Name	Organism	Position	Strand	Matrix score.	sequence	function
TGACG-motif	Hordeum vulgare	862	+	5	TGACG	cis-acting regulatory element involved in the MeJA-responsiveness

+ dOCT

Site Name	Organism	Position	Strand	Matrix score.	sequence	function
dOCT	Arabidopsis thaliana	355	-	8	CaCGGATC	cis-acting regulatory element related to meristem specific activation

Mapoly0069s0004 (*Marchantia polymorpha*)

+ ABRE

Site Name	Organism	Position	Strand	Matrix score.	sequence	function
ABRE	Oryza sativa	136	+	9	GCCGCGTGGC	cis-acting element involved in the abscisic acid responsiveness
ABRE	Arabidopsis thaliana	774	-	6	TACGTG	cis-acting element involved in the abscisic acid responsiveness
ABRE	Hordeum vulgare	772	-	9	CGTACGTGCA	cis-acting element involved in the abscisic acid responsiveness

+ ARE

Site Name	Organism	Position	Strand	Matrix score.	sequence	function
ARE	Zea mays	168	-	6	TGGTTT	cis-acting regulatory element essential for the anaerobic induction
ARE	Zea mays	852	+	6	TGGTTT	cis-acting regulatory element essential for the anaerobic induction

+ Box-W1

Site Name	Organism	Position	Strand	Matrix score.	sequence	function
Box-W1	Petroselinum crispum	1162	-	6	TTGACC	fungal elicitor responsive element

+ CE3

Site Name	Organism	Position	Strand	Matrix score.	sequence	function
CE3	Oryza sativa	1018	-	9	GACGCGTGTC	cis-acting element involved in ABA and VP1 responsiveness

+ CGTCA-motif

Site Name	Organism	Position	Strand	Matrix score.	sequence	function
CGTCA-motif	Hordeum vulgare	1047	-	5	CGTCA	cis-acting regulatory element involved in the MeJA-responsiveness

+ ERE

Site Name	Organism	Position	Strand	Matrix score.	sequence	function
ERE	Dianthus caryophyllus	124	-	8	ATTTCAAA	ethylene-responsive element
ERE	Dianthus caryophyllus	1120	-	8	ATTTCAAA	ethylene-responsive element

+ GCN4_motif

Site Name	Organism	Position	Strand	Matrix score.	sequence	function
GCN4_motif	Oryza sativa	1088	+	7	TGTGTCA	cis-regulatory element involved in endosperm expression

+ HSE

Site Name	Organism	Position	Strand	Matrix score.	sequence	function
HSE	Brassica oleracea	967	+	9	AGAAAATTCG	cis-acting element involved in heat stress responsiveness

+ LTR

Site Name	Organism	Position	Strand	Matrix score.	sequence	function
LTR	Hordeum vulgare	1236	-	6	CCGAAA	cis-acting element involved in low-temperature responsiveness

+ MBS

Site Name	Organism	Position	Strand	Matrix score.	sequence	function
MBS	Arabidopsis thaliana	1335	-	6	TAACTG	MYB binding site involved in drought-inducibility

+ Skn-1_motif

Site Name	Organism	Position	Strand	Matrix score.	sequence	function
Skn-1_motif	Oryza sativa	528	-	5	GTCAT	cis-acting regulatory element required for endosperm expression
Skn-1_motif	Oryza sativa	1112	+	5	GTCAT	cis-acting regulatory element required for endosperm expression
Skn-1_motif	Oryza sativa	847	+	5	GTCAT	cis-acting regulatory element required for endosperm expression
Skn-1_motif	Oryza sativa	1449	-	5	GTCAT	cis-acting regulatory element required for endosperm expression
Skn-1_motif	Oryza sativa	611	-	5	GTCAT	cis-acting regulatory element required for endosperm expression
Skn-1_motif	Oryza sativa	1279	+	5	GTCAT	cis-acting regulatory element required for endosperm expression
Skn-1_motif	Oryza sativa	1046	-	5	GTCAT	cis-acting regulatory element required for endosperm expression

+ TATC-box

Site Name	Organism	Position	Strand	Matrix score.	sequence	function
TATC-box	Oryza sativa	1144	+	7	TATCCCA	cis-acting element involved in

gibberellin-responsiveness

+ TGACG-motif

Site Name	Organism	Position	Strand	Matrix score.	sequence	function
TGACG-motif	Hordeum vulgare	1047	+	5	TGACG	cis-acting regulatory element involved in the MeJA-responsiveness

44451 (*Micromonas pusillaCCMP1545*)

+ ABRE

Site Name	Organism	Position	Strand	Matrix score.	sequence	function
ABRE	Hordeum vulgare	721	+	9	CCGCGTAGGC	cis-acting element involved in the abscisic acid responsiveness
ABRE	Hordeum vulgare	1051	+	9	GCAACGTGTC	cis-acting element involved in the abscisic acid responsiveness
ABRE	Hordeum vulgare	872	-	9	CCGCGTAGGC	cis-acting element involved in the abscisic acid responsiveness

+ ARE

Site Name	Organism	Position	Strand	Matrix score.	sequence	function
ARE	Zea mays	1226	+	6	TGGTTT	cis-acting regulatory element essential for the anaerobic induction

+ Box-W1

Site Name	Organism	Position	Strand	Matrix score.	sequence	function
Box-W1	Petroselinum crispum	1194	-	6	TTGACC	fungal elicitor responsive element
Box-W1	Petroselinum crispum	1201	+	6	TTGACC	fungal elicitor responsive element

+ CCGTCC-box

Site Name	Organism	Position	Strand	Matrix score.	sequence	function
CCGTCC-box	Arabidopsis thaliana	669	+	6	CCGTCC	cis-acting regulatory element related to meristem specific activation
CCGTCC-box	Arabidopsis thaliana	1024	+	6	CCGTCC	cis-acting regulatory element related to meristem specific activation
CCGTCC-box	Arabidopsis thaliana	678	+	6	CCGTCC	cis-acting regulatory element related to meristem specific activation

+ CGTCA-motif

Site Name	Organism	Position	Strand	Matrix score.	sequence	function
CGTCA-motif	Hordeum vulgare	100	-	5	CGTCA	cis-acting regulatory element involved in the MeJA-responsiveness
CGTCA-motif	Hordeum vulgare	979	-	5	CGTCA	cis-acting regulatory element involved in the MeJA-responsiveness
CGTCA-motif	Hordeum vulgare	329	+	5	CGTCA	cis-acting regulatory element involved in the MeJA-responsiveness

CGTCA-motif	Hordeum vulgare	1456	+	5	CGTCA	cis-acting regulatory element involved in the MeJA-responsiveness

+ EIRE

Site Name	Organism	Position	Strand	Matrix score.	sequence	function
EIRE	Nicotiana tabacum	411	+	7	TTCGACC	elicitor-responsive element

+ GC-motif

Site Name	Organism	Position	Strand	Matrix score.	sequence	function
GC-motif	Zea mays	323	+	6	CCCCCG	enhancer-like element involved in anoxic specific inducibility
GC-motif	Zea mays	1306	+	7	GCCCCGG	enhancer-like element involved in anoxic specific inducibility

+ MBS

Site Name	Organism	Position	Strand	Matrix score.	sequence	function
MBS	Arabidopsis thaliana	1177	-	6	CAACTG	MYB binding site involved in drought-inducibility

+ OCT

Site Name	Organism	Position	Strand	Matrix score.	sequence	function
OCT	Arabidopsis thaliana	825	-	8	CGCGGATC	cis-acting regulatory element related to meristem specific activation

+ Skn-1_motif

Site Name	Organism	Position	Strand	Matrix score.	sequence	function
Skn-1_motif	Oryza sativa	27	-	5	GTCAT	cis-acting regulatory element required for endosperm expression
Skn-1_motif	Oryza sativa	1457	+	5	GTCAT	cis-acting regulatory element required for endosperm expression

+ TGA-element

Site Name	Organism	Position	Strand	Matrix score.	sequence	function
TGA-element	Brassica oleracea	897	+	6	AACGAC	auxin-responsive element

+ TGACG-motif

Site Name	Organism	Position	Strand	Matrix score.	sequence	function
TGACG-motif	Hordeum	100	+	5	TGACG	cis-acting regulatory element involved in the

	vulgare					MeJA-responsiveness
TGACG-motif	Hordeum vulgare	979	+	5	TGACG	cis-acting regulatory element involved in the MeJA-responsiveness
TGACG-motif	Hordeum vulgare	329	-	5	TGACG	cis-acting regulatory element involved in the MeJA-responsiveness
TGACG-motif	Hordeum vulgare	1456	-	5	TGACG	cis-acting regulatory element involved in the MeJA-responsiveness

+ as-2-box

Site Name	Organism	Position	Strand	Matrix score.	sequence	function
as-2-box	Nicotiana tabacum	164	-	9	GATAatGATG	involved in shoot-specific expression and light responsiveness

63373 (*Micromonas spRCC299*)

+ ABRE

Site Name	Organism	Position	Strand	Matrix score.	sequence	function
ABRE	Arabidopsis thaliana	719	-	6	TACGTG	cis-acting element involved in the abscisic acid responsiveness
ABRE	Hordeum vulgare	1158	-	9	CCGCGTAGGC	cis-acting element involved in the abscisic acid responsiveness

+ CAT-box

Site Name	Organism	Position	Strand	Matrix score.	sequence	function
CAT-box	Arabidopsis thaliana	1467	+	6	GCCACT	cis-acting regulatory element related to meristem expression

+ CCGTCC-box

Site Name	Organism	Position	Strand	Matrix score.	sequence	function
CCGTCC-box	Arabidopsis thaliana	246	+	6	CCGTCC	cis-acting regulatory element related to meristem specific activation
CCGTCC-box	Arabidopsis thaliana	1102	-	6	CCGTCC	cis-acting regulatory element related to meristem specific activation

+ CGTCA-motif

Site Name	Organism	Position	Strand	Matrix score.	sequence	function
CGTCA-motif	Hordeum vulgare	1	+	5	CGTCA	cis-acting regulatory element involved in the MeJA-responsiveness

+ GC-motif

Site Name	Organism	Position	Strand	Matrix score.	sequence	function
GC-motif	Zea mays	943	-	6	CCCCCG	enhancer-like element involved in anoxic specific inducibility
GC-motif	Zea mays	1226	-	6	CCCCCG	enhancer-like element involved in anoxic specific inducibility
GC-motif	Zea mays	1144	-	6	CCCCCG	enhancer-like element involved in anoxic specific inducibility
GC-motif	Zea mays	1255	-	6	CCCCCG	enhancer-like element involved in anoxic specific inducibility

+ LTR

Site Name	Organism	Position	Strand	Matrix score.	sequence	function
LTR	Hordeum	453	-	6	CCGAAA	cis-acting element involved in low-temperature

	vulgare					responsiveness
LTR	Hordeum vulgare	1267	+	6	CCGAAA	cis-acting element involved in low-temperature responsiveness

+ OCT

Site Name	Organism	Position	Strand	Matrix score.	sequence	function
OCT	Arabidopsis thaliana	1232	+	8	CGCGGATC	cis-acting regulatory element related to meristem specific activation

+ Skn-1_motif

Site Name	Organism	Position	Strand	Matrix score.	sequence	function
Skn-1_motif	Oryza sativa	1089	+	5	GTCAT	cis-acting regulatory element required for endosperm expression

+ TGACG-motif

Site Name	Organism	Position	Strand	Matrix score.	sequence	function
TGACG-motif	Hordeum vulgare	1	-	5	TGACG	cis-acting regulatory element involved in the MeJA-responsiveness

13459 (*Ostreococcus lucimarinus*)

+ ABRE

Site Name	Organism	Position	Strand	Matrix score.	sequence	function
ABRE	Arabidopsis thaliana	110	-	7	ACGTGGC	cis-acting element involved in the abscisic acid responsiveness

+ CCGTCC-box

Site Name	Organism	Position	Strand	Matrix score.	sequence	function
CCGTCC-box	Arabidopsis thaliana	700	+	6	CCGTCC	cis-acting regulatory element related to meristem specific activation
CCGTCC-box	Arabidopsis thaliana	963	+	6	CCGTCC	cis-acting regulatory element related to meristem specific activation
CCGTCC-box	Arabidopsis thaliana	945	+	6	CCGTCC	cis-acting regulatory element related to meristem specific activation
CCGTCC-box	Arabidopsis thaliana	969	+	6	CCGTCC	cis-acting regulatory element related to meristem specific activation

+ CGTCA-motif

Site Name	Organism	Position	Strand	Matrix score.	sequence	function
CGTCA-motif	Hordeum vulgare	4	-	5	CGTCA	cis-acting regulatory element involved in the MeJA-responsiveness
CGTCA-motif	Hordeum vulgare	914	+	5	CGTCA	cis-acting regulatory element involved in the MeJA-responsiveness
CGTCA-motif	Hordeum vulgare	392	+	5	CGTCA	cis-acting regulatory element involved in the MeJA-responsiveness

+ NON-box

Site Name	Organism	Position	Strand	Matrix score.	sequence	function
NON-box	Arabidopsis thaliana	364	-	9	AGATCGACG	cis-acting regulatory element related to meristem specific activation

+ Skn-1_motif

Site Name	Organism	Position	Strand	Matrix score.	sequence	function
Skn-1_motif	Oryza sativa	3	-	5	GTCAT	cis-acting regulatory element required for endosperm expression
Skn-1_motif	Oryza sativa	411	+	5	GTCAT	cis-acting regulatory element required for endosperm expression

+ TC-rich repeats

Site Name	Organism	Position	Strand	Matrix	sequence	function

				score.		
TC-rich repeats	Nicotiana tabacum	462	+	9	ATTTTCTTCA	cis-acting element involved in defense and stress responsiveness

+ TGACG-motif

Site Name	Organism	Position	Strand	Matrix score.	sequence	function
TGACG-motif	Hordeum vulgare	4	+	5	TGACG	cis-acting regulatory element involved in the MeJA-responsiveness
TGACG-motif	Hordeum vulgare	914	-	5	TGACG	cis-acting regulatory element involved in the MeJA-responsiveness
TGACG-motif	Hordeum vulgare	392	-	5	TGACG	cis-acting regulatory element involved in the MeJA-responsiveness

+ dOCT

Site Name	Organism	Position	Strand	Matrix score.	sequence	function
dOCT	Arabidopsis thaliana	167	+	8	CaCGGATC	cis-acting regulatory element related to meristem specific activation

53193 (*Coccomyxa subellipsoidea*)

+ ABRE

Site Name	Organism	Position	Strand	Matrix score.	sequence	function
ABRE	Arabidopsis thaliana	268	-	7	ACGTGGC	cis-acting element involved in the abscisic acid responsiveness
ABRE	Arabidopsis thaliana	1068	-	6	CACGTG	cis-acting element involved in the abscisic acid responsiveness
ABRE	Arabidopsis thaliana	270	-	6	TACGTG	cis-acting element involved in the abscisic acid responsiveness
ABRE	Arabidopsis thaliana	1177	-	6	CACGTG	cis-acting element involved in the abscisic acid responsiveness

+ AuxRE

Site Name	Organism	Position	Strand	Matrix score.	sequence	function
AuxRE	Glycine max	1424	+	11	TGTCTCAATAAG	part of an auxin-responsive element

+ AuxRR-core

Site Name	Organism	Position	Strand	Matrix score.	sequence	function
AuxRR-core	Nicotiana tabacum	99	-	7	GGTCCAT	cis-acting regulatory element involved in auxin responsiveness
AuxRR-core	Nicotiana tabacum	1213	-	7	GGTCCAT	cis-acting regulatory element involved in auxin responsiveness
AuxRR-core	Nicotiana tabacum	1138	+	7	GGTCCAT	cis-acting regulatory element involved in auxin responsiveness

+ CAT-box

Site Name	Organism	Position	Strand	Matrix score.	sequence	function
CAT-box	Arabidopsis thaliana	597	-	6	GCCACT	cis-acting regulatory element related to meristem expression
CAT-box	Arabidopsis thaliana	1233	+	6	GCCACT	cis-acting regulatory element related to meristem expression

+ CGTCA-motif

Site Name	Organism	Position	Strand	Matrix score.	sequence	function
CGTCA-motif	Hordeum vulgare	294	+	5	CGTCA	cis-acting regulatory element involved in the MeJA-responsiveness
CGTCA-motif	Hordeum vulgare	879	-	5	CGTCA	cis-acting regulatory element involved in the MeJA-responsiveness

+ GC-motif

Site Name	Organism	Position	Strand	Matrix score.	sequence	function
GC-motif	Zea mays	154	-	6	CCCCCG	enhancer-like element involved in anoxic specific inducibility
GC-motif	Zea mays	197	+	6	CCCCCG	enhancer-like element involved in anoxic specific inducibility
GC-motif	Zea mays	1270	-	8	CCACGGGG	enhancer-like element involved in anoxic specific inducibility

+ MBS

Site Name	Organism	Position	Strand	Matrix score.	sequence	function
MBS	Arabidopsis thaliana	7	-	6	CAACTG	MYB binding site involved in drought-inducibility
MBS	Arabidopsis thaliana	704	-	6	CAACTG	MYB binding site involved in drought-inducibility
MBS	Arabidopsis thaliana	408	-	6	CAACTG	MYB binding site involved in drought-inducibility
MBS	Arabidopsis thaliana	1291	-	6	CAACTG	MYB binding site involved in drought-inducibility

+ RY-element

Site Name	Organism	Position	Strand	Matrix score.	sequence	function
RY-element	Helianthus annuus	979	-	8	CATGCATG	cis-acting regulatory element involved in seed-specific regulation

+ Skn-1_motif

Site Name	Organism	Position	Strand	Matrix score.	sequence	function
Skn-1_motif	Oryza sativa	181	+	5	GTCAT	cis-acting regulatory element required for endosperm expression
Skn-1_motif	Oryza sativa	349	-	5	GTCAT	cis-acting regulatory element required for endosperm expression
Skn-1_motif	Oryza sativa	295	+	5	GTCAT	cis-acting regulatory element required for endosperm expression

+ TC-rich repeats

Site Name	Organism	Position	Strand	Matrix score.	sequence	function
TC-rich repeats	Nicotiana tabacum	1011	-	9	ATTTTCTTCA	cis-acting element involved in defense and stress responsiveness

+ TCA-element

Site Name	Organism	Position	Strand	Matrix	sequence	function

				score.		
TCA-element	Nicotiana tabacum	259	-	9	CCATCTTTTT	cis-acting element involved in salicylic acid responsiveness

+ TGACG-motif

Site Name	Organism	Position	Strand	Matrix score.	sequence	function
TGACG-motif	Hordeum vulgare	294	-	5	TGACG	cis-acting regulatory element involved in the MeJA-responsiveness
TGACG-motif	Hordeum vulgare	879	+	5	TGACG	cis-acting regulatory element involved in the MeJA-responsiveness

+ dOCT

Site Name	Organism	Position	Strand	Matrix score.	sequence	function
dOCT	Arabidopsis thaliana	1166	+	8	CaCGGATC	cis-acting regulatory element related to meristem specific activation

Dusal.0694s00005 (*Dunaliella salina*)

+ ARE

Site Name	Organism	Position	Strand	Matrix score.	sequence	function
ARE	Zea mays	1457	-	6	TGGTTT	cis-acting regulatory element essential for the anaerobic induction

+ ERE

Site Name	Organism	Position	Strand	Matrix score.	sequence	function
ERE	Dianthus caryophyllus	1405	+	8	ATTTCAAA	ethylene-responsive element

+ GC-motif

Site Name	Organism	Position	Strand	Matrix score.	sequence	function
GC-motif	Zea mays	151	+	6	CCCCCG	enhancer-like element involved in anoxic specific inducibility
GC-motif	Zea mays	333	+	6	CCCCCG	enhancer-like element involved in anoxic specific inducibility
GC-motif	Zea mays	242	+	6	CCCCCG	enhancer-like element involved in anoxic specific inducibility
GC-motif	Zea mays	424	+	6	CCCCCG	enhancer-like element involved in anoxic specific inducibility

+ MBS

Site Name	Organism	Position	Strand	Matrix score.	sequence	function
MBS	Arabidopsis thaliana	439	-	6	CAACTG	MYB binding site involved in drought-inducibility
MBS	Arabidopsis thaliana	1088	+	6	CAACTG	MYB binding site involved in drought-inducibility
MBS	Arabidopsis thaliana	795	+	6	CAACTG	MYB binding site involved in drought-inducibility
MBS	Arabidopsis thaliana	1479	-	6	CAACTG	MYB binding site involved in drought-inducibility

+ P-box

Site Name	Organism	Position	Strand	Matrix score.	sequence	function
P-box	Oryza sativa	121	+	7	CCTTTTG	gibberellin-responsive element

+ Skn-1_motif

Site Name	Organism	Position	Strand	Matrix score.	sequence	function

Skn-1_motif	Oryza sativa	1082	+	5	GTCAT	cis-acting regulatory element required for endosperm expression

+ TC-rich repeats

Site Name	Organism	Position	Strand	Matrix score.	sequence	function
TC-rich repeats	Nicotiana tabacum	950	+	9	ATTTTCTTCA	cis-acting element involved in defense and stress responsiveness

+ as-2-box

Site Name	Organism	Position	Strand	Matrix score.	sequence	function
as-2-box	Nicotiana tabacum	863	+	9	GATAatGATG	involved in shoot-specific expression and light responsiveness

Vocar.0038s0046 (*Volvox carteri*)

+ ARE

Site Name	Organism	Position	Strand	Matrix score.	sequence	function
ARE	Zea mays	217	-	6	TGGTTT	cis-acting regulatory element essential for the anaerobic induction

+ Box-W1

Site Name	Organism	Position	Strand	Matrix score.	sequence	function
Box-W1	Petroselinum crispum	408	+	6	TTGACC	fungal elicitor responsive element

+ CAT-box

Site Name	Organism	Position	Strand	Matrix score.	sequence	function
CAT-box	Arabidopsis thaliana	403	+	6	GCCACT	cis-acting regulatory element related to meristem expression

+ CCGTCC-box

Site Name	Organism	Position	Strand	Matrix score.	sequence	function
CCGTCC-box	Arabidopsis thaliana	146	+	6	CCGTCC	cis-acting regulatory element related to meristem specific activation

+ CGTCA-motif

Site Name	Organism	Position	Strand	Matrix score.	sequence	function
CGTCA-motif	Hordeum vulgare	11	-	5	CGTCA	cis-acting regulatory element involved in the MeJA-responsiveness
CGTCA-motif	Hordeum vulgare	1236	-	5	CGTCA	cis-acting regulatory element involved in the MeJA-responsiveness
CGTCA-motif	Hordeum vulgare	584	+	5	CGTCA	cis-acting regulatory element involved in the MeJA-responsiveness

+ GC-motif

Site Name	Organism	Position	Strand	Matrix score.	sequence	function
GC-motif	Zea mays	412	+	6	CCCCCG	enhancer-like element involved in anoxic specific inducibility
GC-motif	Zea mays	1245	+	6	CCCCCG	enhancer-like element involved in anoxic specific inducibility

+ LTR

Site Name	Organism	Position	Strand	Matrix score.	sequence	function

LTR	Hordeum vulgare	377	+	6	CCGAAA	cis-acting element involved in low-temperature responsiveness
LTR	Hordeum vulgare	801	+	6	CCGAAA	cis-acting element involved in low-temperature responsiveness

+ MBS

Site Name	Organism	Position	Strand	Matrix score.	sequence	function
MBS	Arabidopsis thaliana	249	+	6	CAACTG	MYB binding site involved in drought-inducibility

+ P-box

Site Name	Organism	Position	Strand	Matrix score.	sequence	function
P-box	Oryza sativa	1486	-	10	GCCTTTTGAGT	gibberellin-responsive element

+ RY-element

Site Name	Organism	Position	Strand	Matrix score.	sequence	function
RY-element	Helianthus annuus	870	-	8	CATGCATG	cis-acting regulatory element involved in seed-specific regulation

+ Skn-1_motif

Site Name	Organism	Position	Strand	Matrix score.	sequence	function
Skn-1_motif	Oryza sativa	140	+	5	GTCAT	cis-acting regulatory element required for endosperm expression
Skn-1_motif	Oryza sativa	588	-	5	GTCAT	cis-acting regulatory element required for endosperm expression
Skn-1_motif	Oryza sativa	585	+	5	GTCAT	cis-acting regulatory element required for endosperm expression
Skn-1_motif	Oryza sativa	1383	-	5	GTCAT	cis-acting regulatory element required for endosperm expression

+ TGA-element

Site Name	Organism	Position	Strand	Matrix score.	sequence	function
TGA-element	Brassica oleracea	104	-	6	AACGAC	auxin-responsive element
TGA-element	Brassica oleracea	1322	-	6	AACGAC	auxin-responsive element

+ TGACG-motif

Site Name	Organism	Position	Strand	Matrix score.	sequence	function
TGACG-motif	Hordeum	11	+	5	TGACG	cis-acting regulatory element involved in the

	vulgare					MeJA-responsiveness
TGACG-motif	Hordeum vulgare	1236	+	5	TGACG	cis-acting regulatory element involved in the MeJA-responsiveness
TGACG-motif	Hordeum vulgare	584	-	5	TGACG	cis-acting regulatory element involved in the MeJA-responsiveness

Cre06.g252700 (*Chlamydomonas reinhardtii*)

+ ABRE

Site Name	Organism	Position	Strand	Matrix score.	sequence	function
ABRE	Arabidopsis thaliana	1427	-	6	CACGTG	cis-acting element involved in the abscisic acid responsiveness

+ Box-W1

Site Name	Organism	Position	Strand	Matrix score.	sequence	function
Box-W1	Petroselinum crispum	1220	-	6	TTGACC	fungal elicitor responsive element

+ C-repeat/DRE

Site Name	Organism	Position	Strand	Matrix score.	sequence	function
C-repeat/DRE	Arabidopsis thaliana	511	+	8	TGGCCGAC	regulatory element involved in cold- and dehydration-responsiveness

+ CCGTCC-box

Site Name	Organism	Position	Strand	Matrix score.	sequence	function
CCGTCC-box	Arabidopsis thaliana	799	-	6	CCGTCC	cis-acting regulatory element related to meristem specific activation

+ CGTCA-motif

Site Name	Organism	Position	Strand	Matrix score.	sequence	function
CGTCA-motif	Hordeum vulgare	955	-	5	CGTCA	cis-acting regulatory element involved in the MeJA-responsiveness

+ ERE

Site Name	Organism	Position	Strand	Matrix score.	sequence	function
ERE	Dianthus caryophyllus	1492	+	8	ATTTCAAA	ethylene-responsive element

+ GC-motif

Site Name	Organism	Position	Strand	Matrix score.	sequence	function
GC-motif	Zea mays	541	-	7	GCCCCGG	enhancer-like element involved in anoxic specific inducibility

+ GCN4_motif

Site Name	Organism	Position	Strand	Matrix score.	sequence	function
GCN4_motif	Oryza sativa	1263	+	7	CAAGCCA	cis-regulatory element involved in endosperm

expression

+ Skn-1_motif

Site Name	Organism	Position	Strand	Matrix score.	sequence	function
Skn-1_motif	Oryza sativa	155	-	5	GTCAT	cis-acting regulatory element required for endosperm expression
Skn-1_motif	Oryza sativa	744	-	5	GTCAT	cis-acting regulatory element required for endosperm expression
Skn-1_motif	Oryza sativa	689	-	5	GTCAT	cis-acting regulatory element required for endosperm expression

+ TGA-element

Site Name	Organism	Position	Strand	Matrix score.	sequence	function
TGA-element	Brassica oleracea	709	-	6	AACGAC	auxin-responsive element

+ TGACG-motif

Site Name	Organism	Position	Strand	Matrix score.	sequence	function
TGACG-motif	Hordeum vulgare	955	+	5	TGACG	cis-acting regulatory element involved in the MeJA-responsiveness
